# Supplementary material for: Targeting pancreatic cancer with combined inhibition of EGFR and RAF
Source: PLoS One. 2026 Apr 24;21(4):e0347843. doi: 10.1371/journal.pone.0347843 (PMC13108728; doi:10.1371/journal.pone.0347843)
Supplement: S1 Fig — (PDF) [file pone.0347843.s001.pdf]

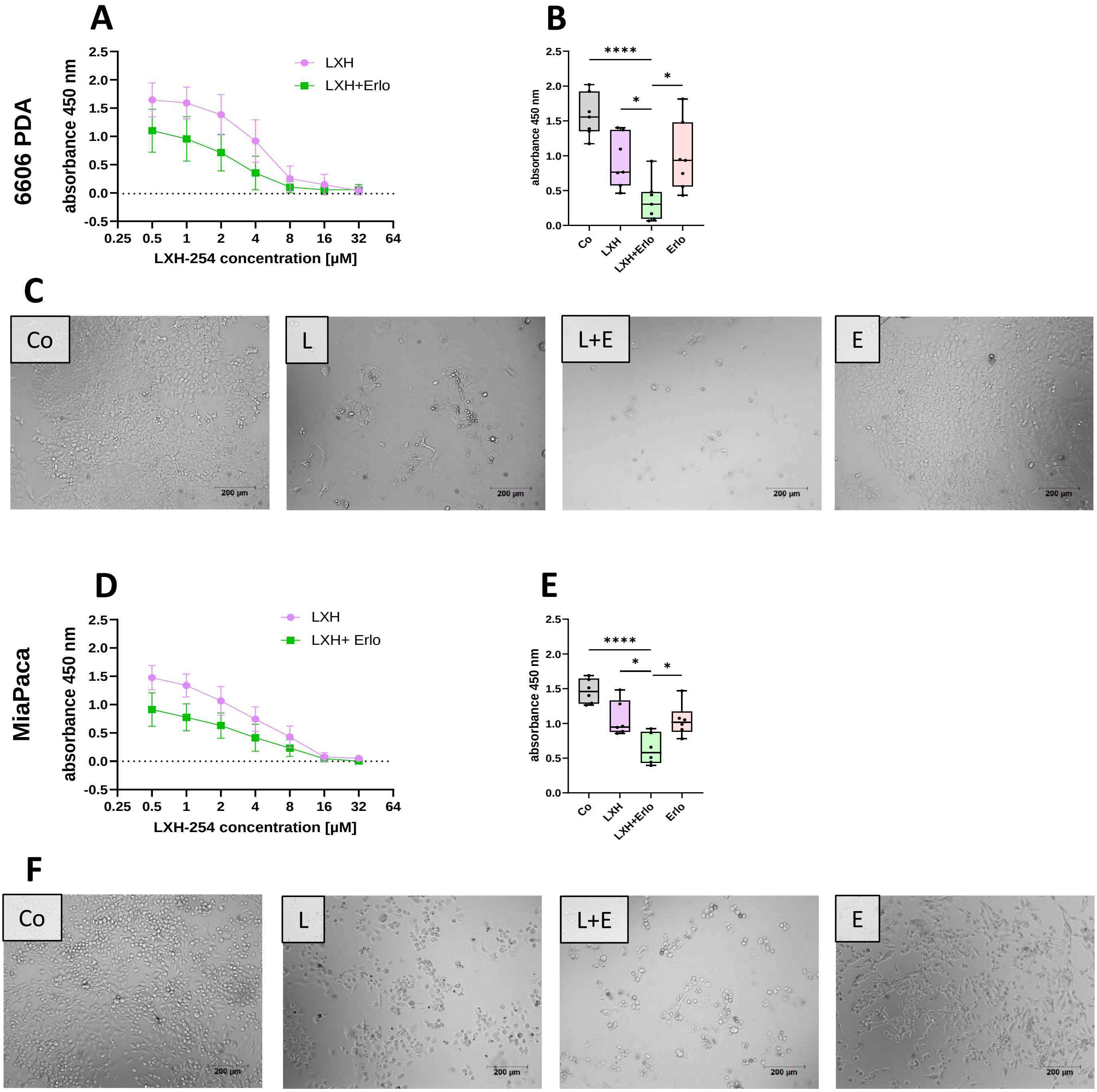

S1 Fig. Anti-cancer effects of the combination therapy on the murine and human pancreatic tumor cell lines by quantifying cell proliferation and cell death after 72 h. Dose-response curve (A) and comparison of treatment groups (B) for 4 µM LXH-254 and combinatorial treatment with 10 µM erlotinib were quantified by proliferation assays in murine 6606PDA cells. Exemplary images of 6606 PDA cells with the different treatments after 72 h incubation time (C). Dose-dependent inhibition of proliferation of human Mia PaCa-2 cells by LXH-254 and the combination with 15 µM erlotinib (D). Differences in Mia PaCa-2 cell proliferation in the vehicle (Co), 2 µM LXH-254 (LXH), 15 µM erlotinib (Erlo) and the combinatorial treatment (LXH+Erlo) (E). Images of MiaPaca cells after treatment for 72 h hours with the mentioned concentrations and therapeutics (F). Statistics were carried out using one-way ANOVA with Tukey-test for multiple comparisons. * p≤0.05, *** p≤0.001, **** p≤0.0001; A, B: n = 7; D: n = 7; E : n = 6.
