# Supplementary material for: Targeting pancreatic cancer with combined inhibition of EGFR and RAF
Source: PLoS One. 2026 Apr 24;21(4):e0347843. doi: 10.1371/journal.pone.0347843 (PMC13108728; doi:10.1371/journal.pone.0347843)
Supplement: S2 Fig — (PDF) [file pone.0347843.s002.pdf]

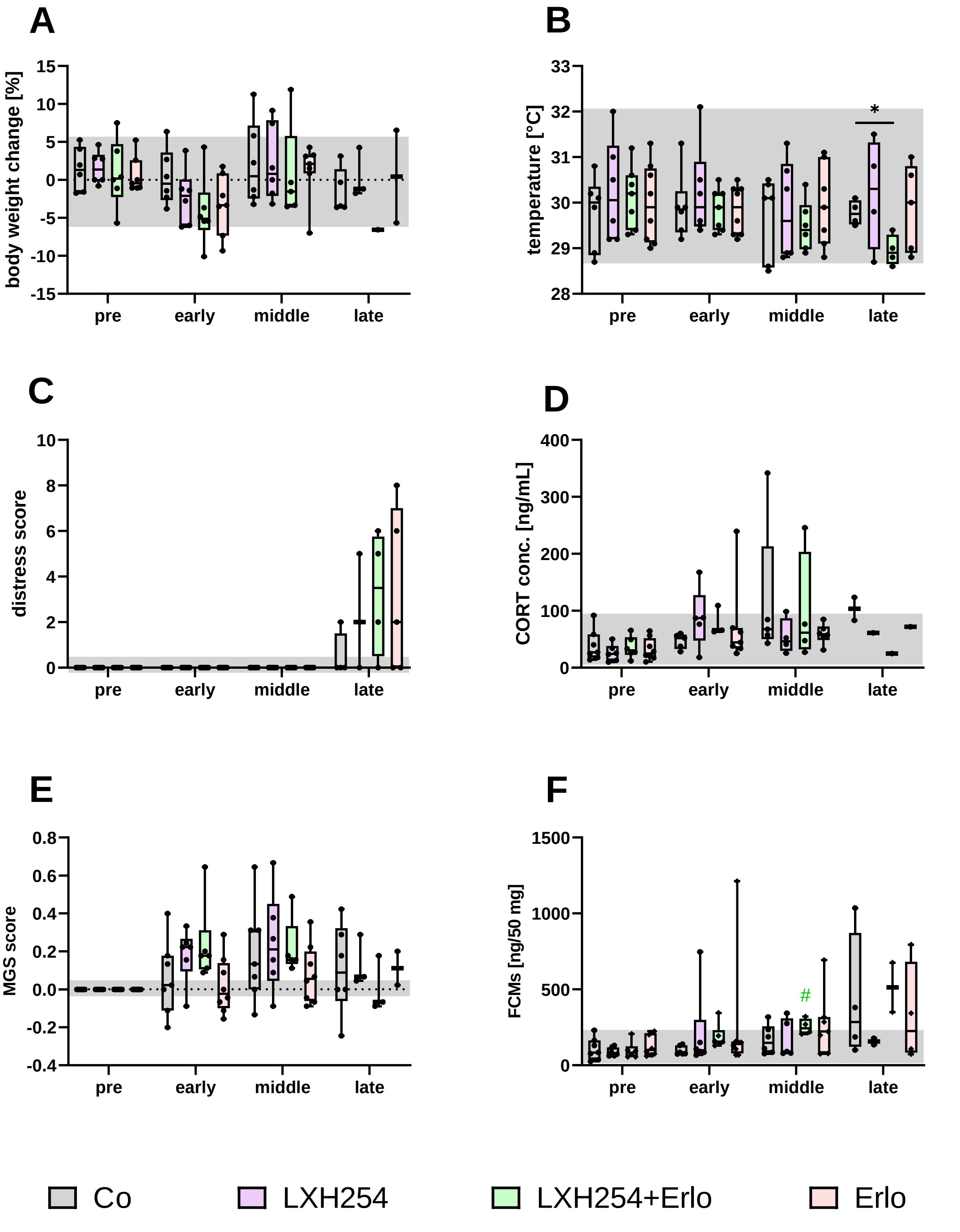

S2 Fig. Multi-parametric evaluation of distress in an orthotopic murine pancreatic model during combination therapy. Potentially occurring distress caused by therapeutic intervention was evaluated before tumor cell injection (pre) and during the early, middle, and late phase of therapeutic intervention, by assessing body weight change (A), perianal temperature (B), distress score (C), corticosterone concentration in the plasma (D), mouse grimace scale score (E) and the concentration of fecal corticosterone metabolites (F). The data were tested for differences between the groups (*p<0.05) as well as longitudinally compared to the baseline value (pre, #p<0.05). The grey area represents the base line measurements on healthy mice. Statistics were carried out using mixed-effects model; control: n = 6; LXH254: n = 6; LXH-254+Erlo: n = 6; Erlo: n = 7.
