# Supplementary material for: Targeting pancreatic cancer with combined inhibition of EGFR and RAF
Source: PLoS One. 2026 Apr 24;21(4):e0347843. doi: 10.1371/journal.pone.0347843 (PMC13108728; doi:10.1371/journal.pone.0347843)
Supplement: S3 Fig — (PDF) [file pone.0347843.s003.pdf]

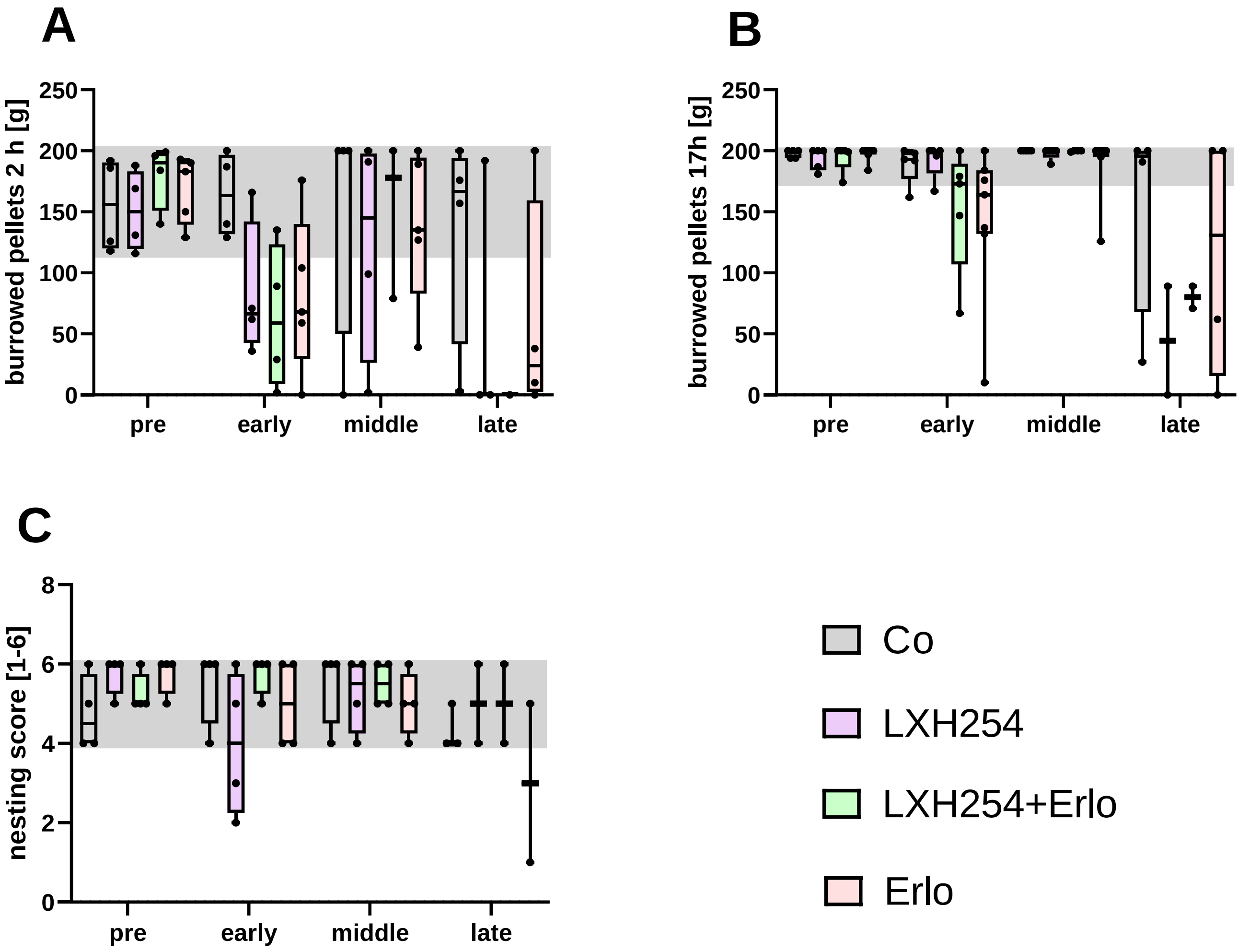

S3 Fig. Analysis of behavioral parameters for welfare assessment in an orthotopic murine pancreatic model during combination therapy. Impairment of animal welfare at the early, middle, and late phase of therapeutic intervention was evaluated by assessing the animals' burrowing performance after 2 hours (**A**), 17 hours (**B**) as well as their nesting activity (**C**). The data were tested for differences between the groups, as well as longitudinally compared to the baseline value (pre). The grey area represents the base line measurements on healthy mice. Statistics were carried out using mixed-effects model; control: n = 4-5; LXH-254: n = 4-5; LXH-254+erlotinib: n = 4-5; erlotinib: n = 4-7.
