# Supplementary material for: Targeting pancreatic cancer with combined inhibition of EGFR and RAF
Source: PLoS One. 2026 Apr 24;21(4):e0347843. doi: 10.1371/journal.pone.0347843 (PMC13108728; doi:10.1371/journal.pone.0347843)
Supplement: S4 Fig — (PDF) [file pone.0347843.s004.pdf]

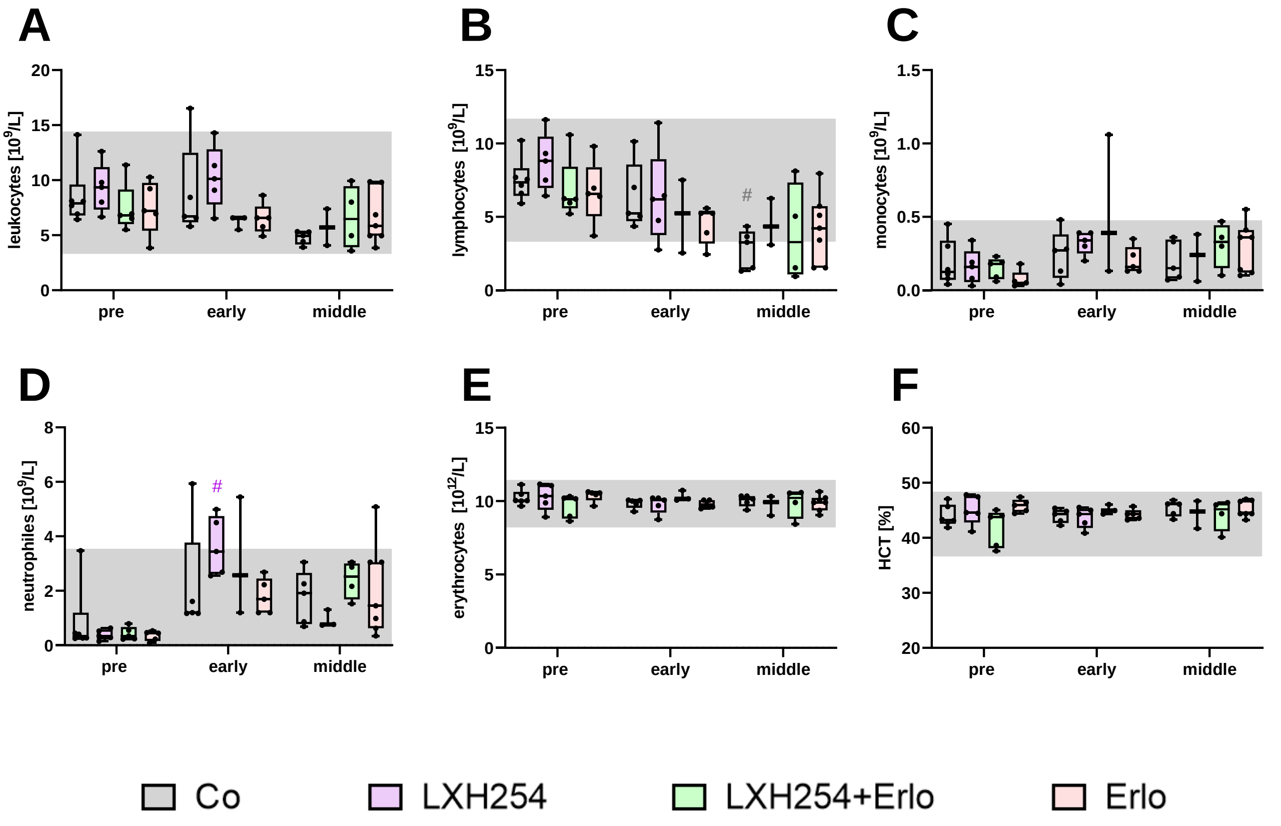

S4 Fig. Blood count before and during the therapy of all treatment groups. Counts of leukocytes (A), lymphocytes (B), monocytes (C), neutrophils (D), erythrocytes (E) and hematocrit (HCT; F) from healthy mice (pre) and during the early and middle phase of tumor progression and therapeutic intervention. Data were tested for differences between the groups (*p<0.05) as well as longitudinally compared to the baseline value (pre, #p<0.05). The grey area represents the base line measurements on healthy mice. Statistics were carried out using mixed-effects model. Control: n = 6; LXH-254: n = 6; LXH 254+erlotinib: n = 4-5; erlotinib: n = 6.
