# Supplementary material for: Targeting pancreatic cancer with combined inhibition of EGFR and RAF
Source: PLoS One. 2026 Apr 24;21(4):e0347843. doi: 10.1371/journal.pone.0347843 (PMC13108728; doi:10.1371/journal.pone.0347843)
Supplement: S5 Fig — (PDF) [file pone.0347843.s005.pdf]

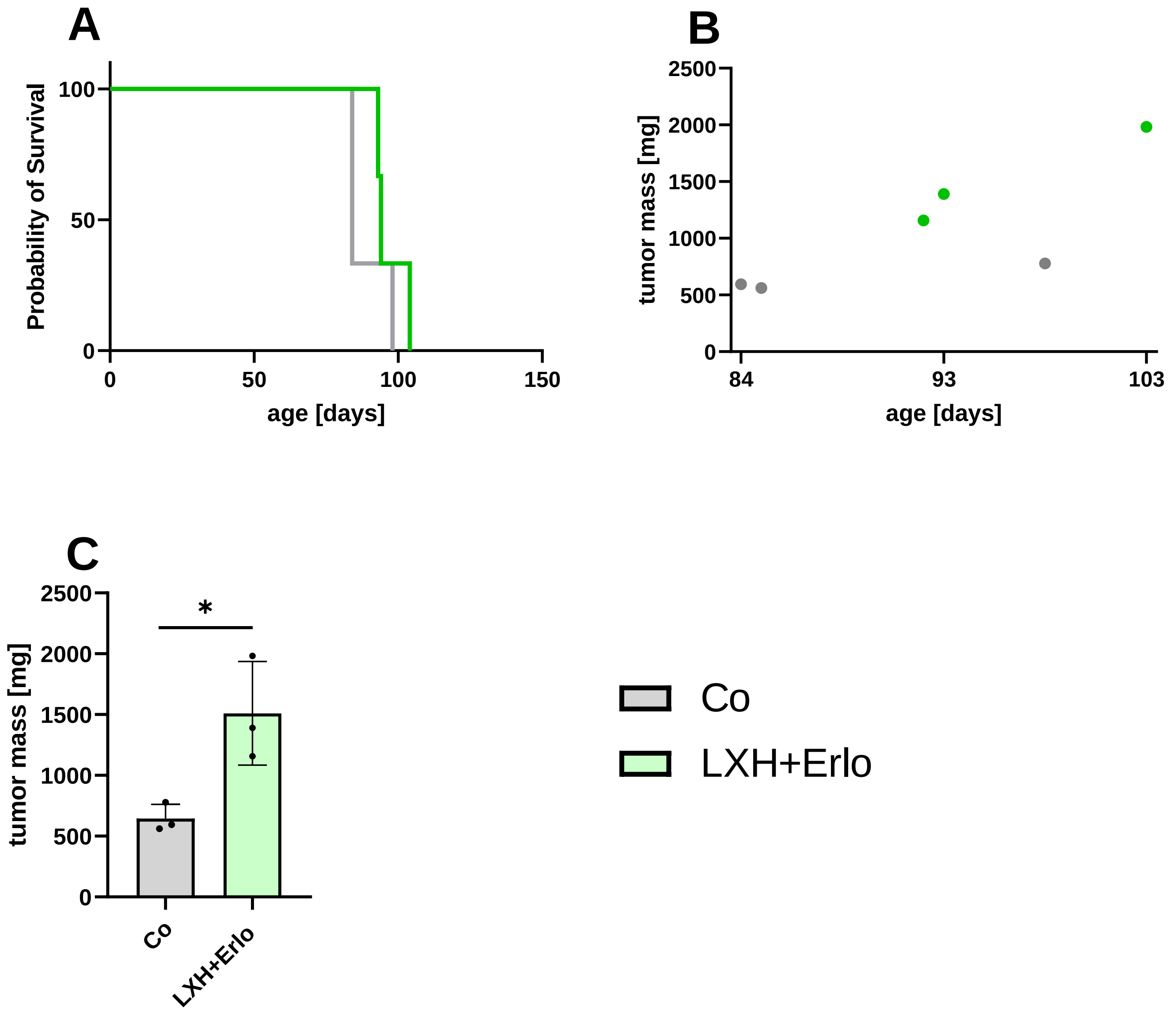

S5 Fig. Quantification of therapeutic efficacy of combinatorial treatment with LXH-254 and erlotinib in a genetic pancreatic cancer model (KPC). Overall survival was quantified in KPC mice after treatment with either LXH-254+erlotinib or vehicle (Co) as soon as a tumor was detected via *in vivo* imaging (**A**). Tumor weight was analyzed at the individual humane endpoint of each mouse (**B**). Averaged tumor mass was displayed for each group (**C**). Statistics were carried out using log-rank test for comparison of survival curves and unpaired t test for tumor weight comparison; control: n = 3; LXH-254+Erlo: n = 3.
