# Supplementary material for: Targeting pancreatic cancer with combined inhibition of EGFR and RAF
Source: PLoS One. 2026 Apr 24;21(4):e0347843. doi: 10.1371/journal.pone.0347843 (PMC13108728; doi:10.1371/journal.pone.0347843)
Supplement: S6 Fig — (PDF) [file pone.0347843.s006.pdf]

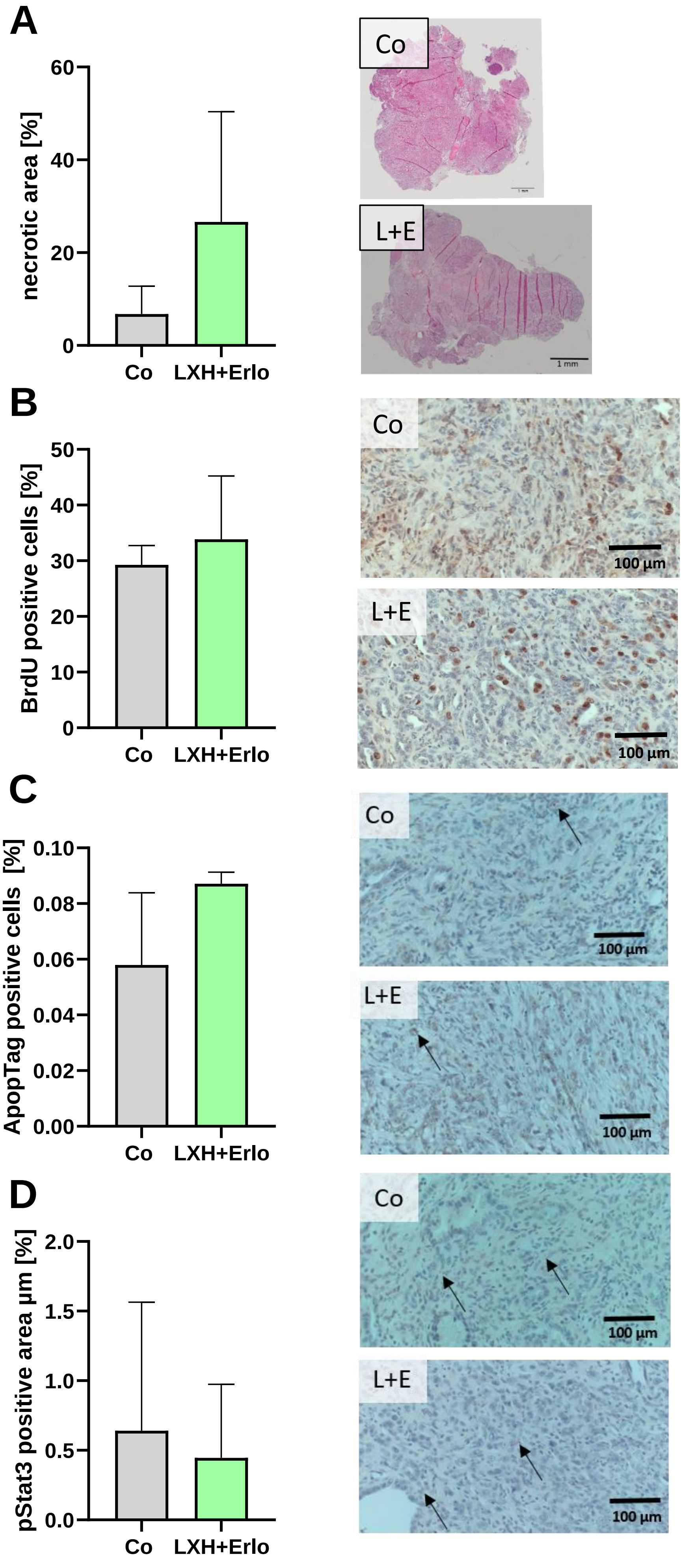

S6Fig. Immunohistological analysis of tumors from treated KPC mice. Quantification of percentages of necrotic area via H/E staining (**A**), proliferating cells by BrdU incorporation (**B**), apoptotic cells via ApopTag® Peroxidase (**C**) and pSTAT3 positive area (**D**), respectively for control or LXH-254 plus erlotinib treated KPC mice. Arrows are marking the positive cells or area. Statistics were carried out using Mann-Whitney test; Co: n=3, LXH+Erlo n=2-3.
