# Supplementary material for: Targeting pancreatic cancer with combined inhibition of EGFR and RAF
Source: PLoS One. 2026 Apr 24;21(4):e0347843. doi: 10.1371/journal.pone.0347843 (PMC13108728; doi:10.1371/journal.pone.0347843)

pEGFR (Panc02 cells + 1 µM LXH-254 06.01.2022)

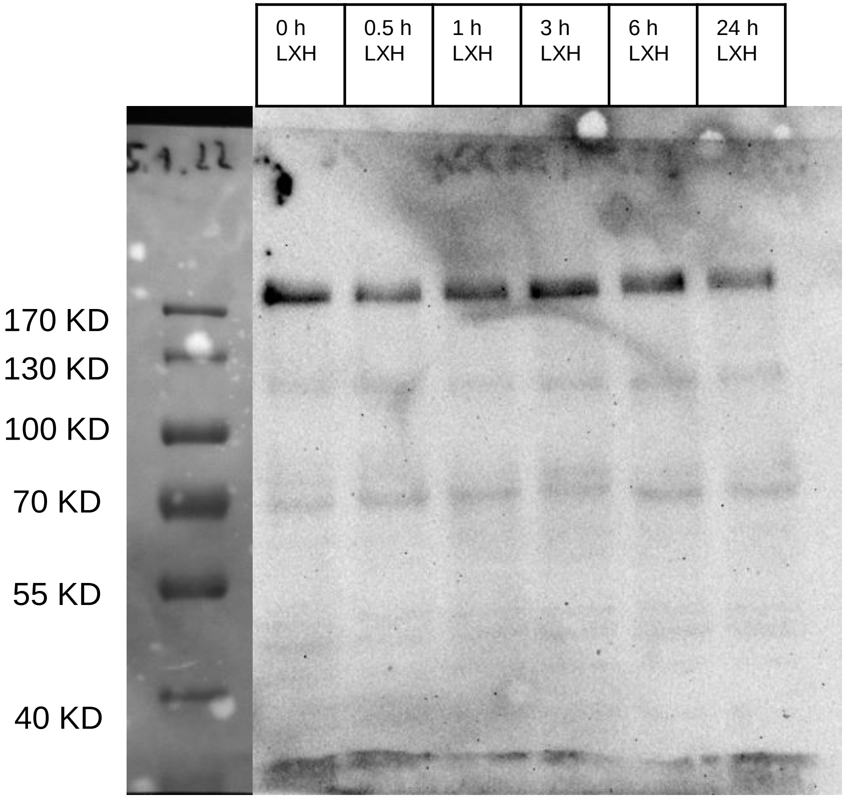

EGFR (Panc02 cells + 1 µM LXH-254 11.01.2022)

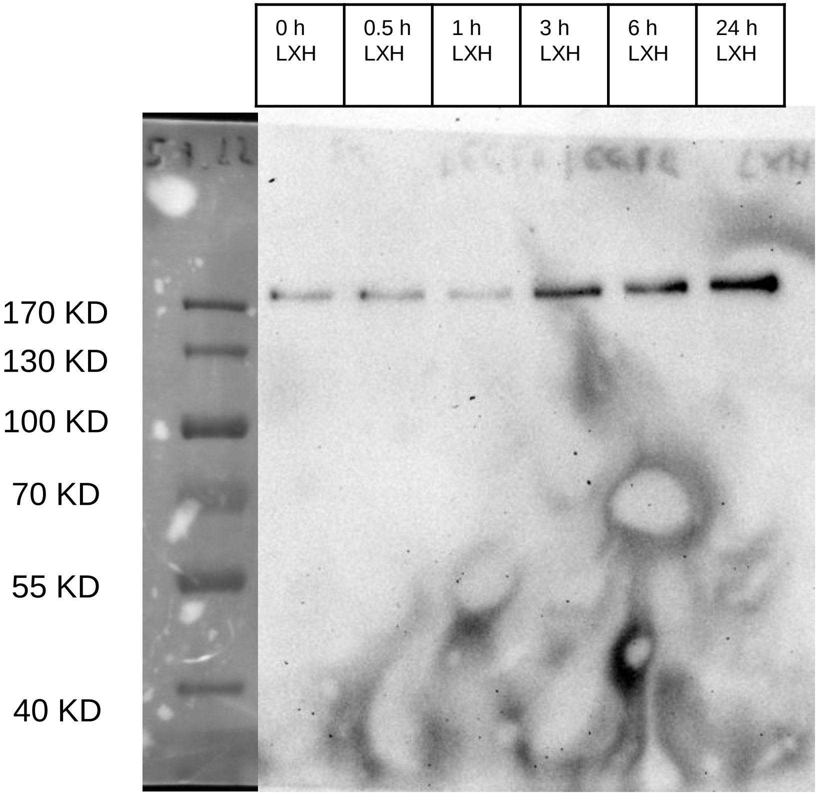

β-actin (pEGFR und EGFR_Panc02 cells + 1 µM LXH-254)
12.01.22

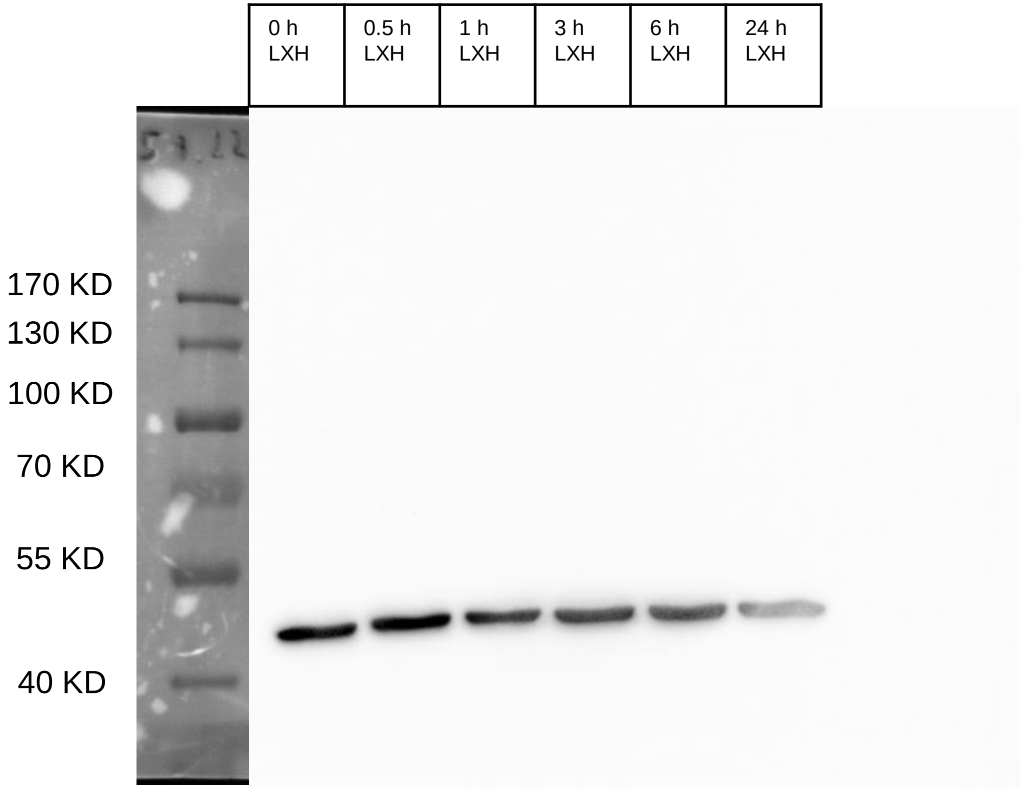

pMEK (Panc02 cells + 1 µM LXH- 254 07.01.2022)

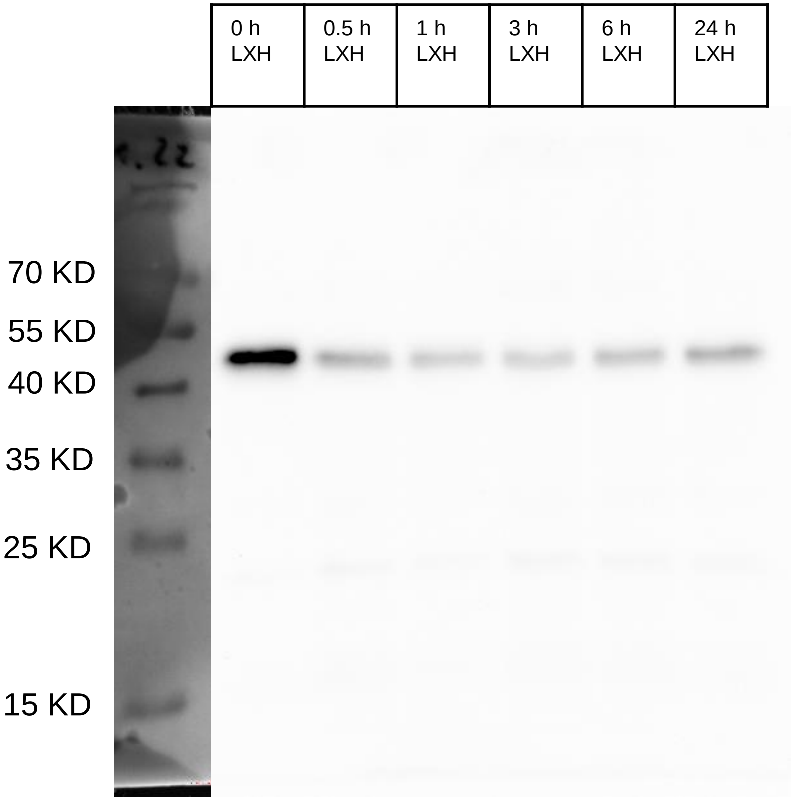

MEK (Panc02 cells + 1µM LXH-254 11.01.2022)

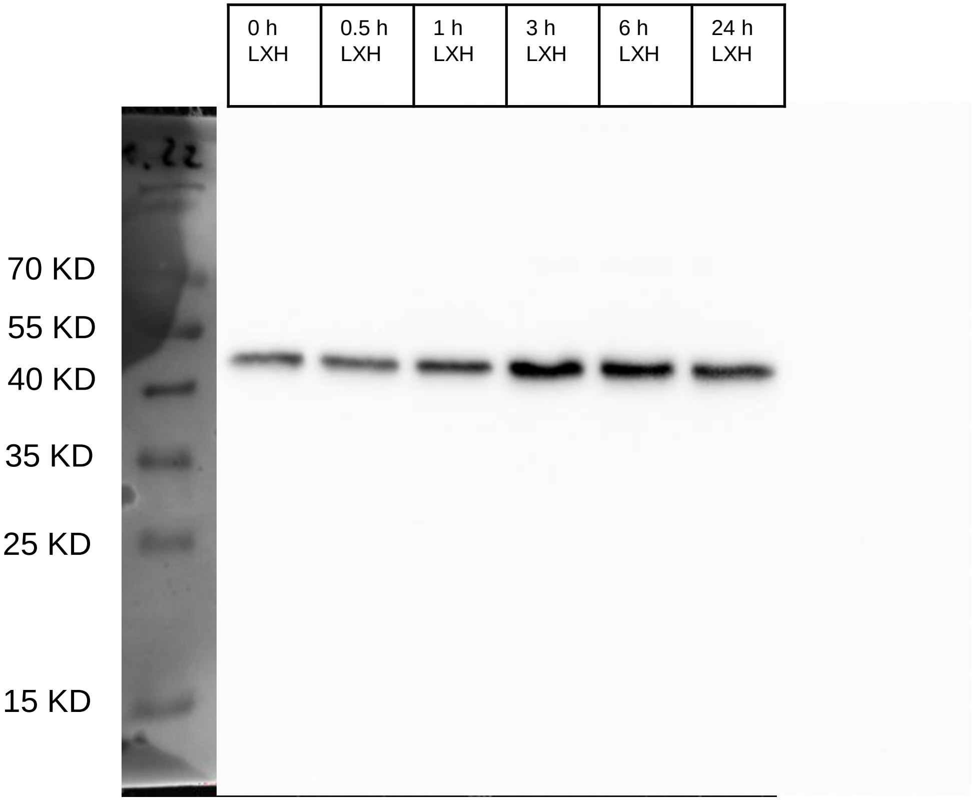

β-actin (pMEK/MEK Panc02 cells + 1µM LXH-254 12.01.2022)

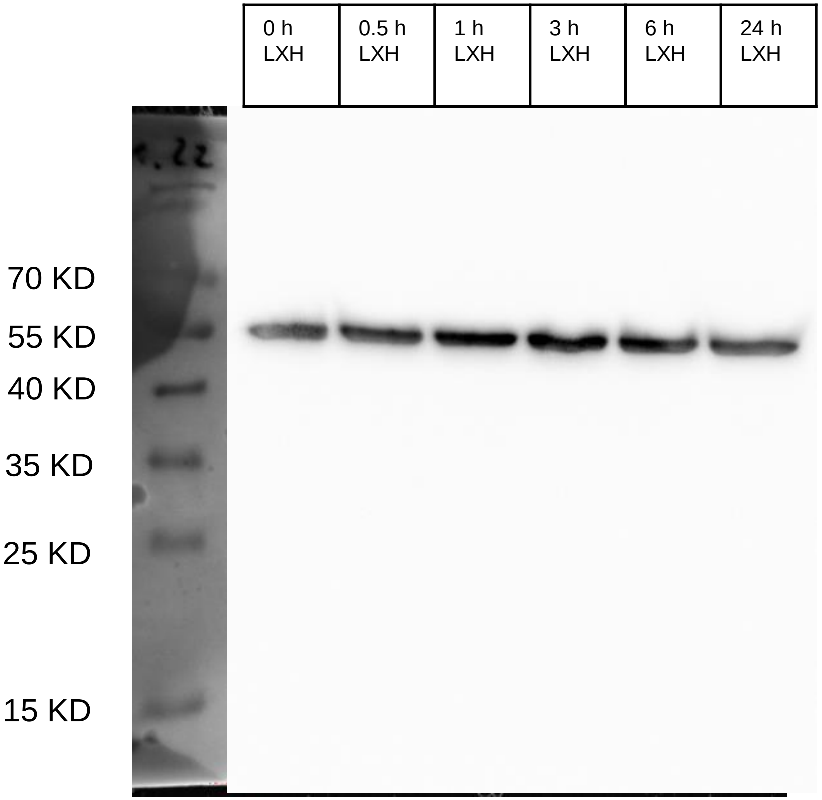

pERK (Panc02 cells + 1 µM LXH-254 12.01.2022)

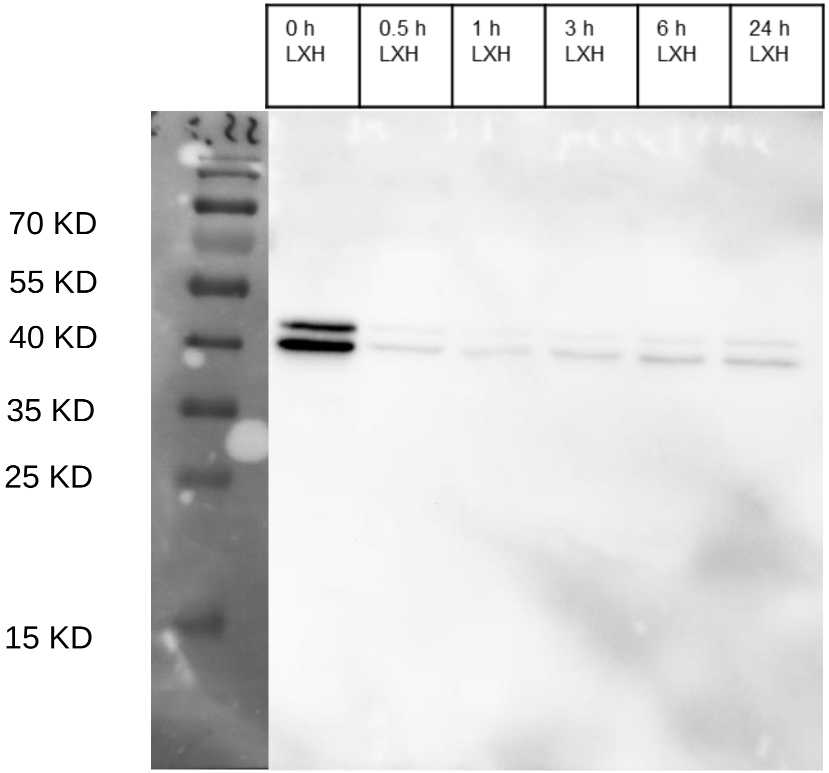

ERK (Panc02 cells + 1 µM LXH-254 18.01.2022)

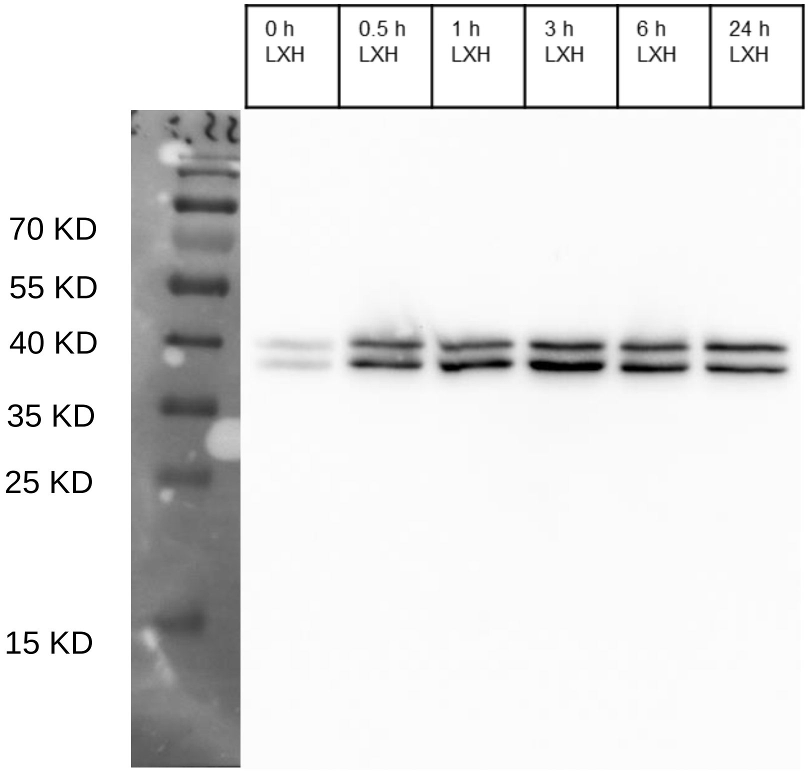

β-actin (pERK/ERK, Panc02-Zellen + 1 µM LXH-254 19.01.2022)

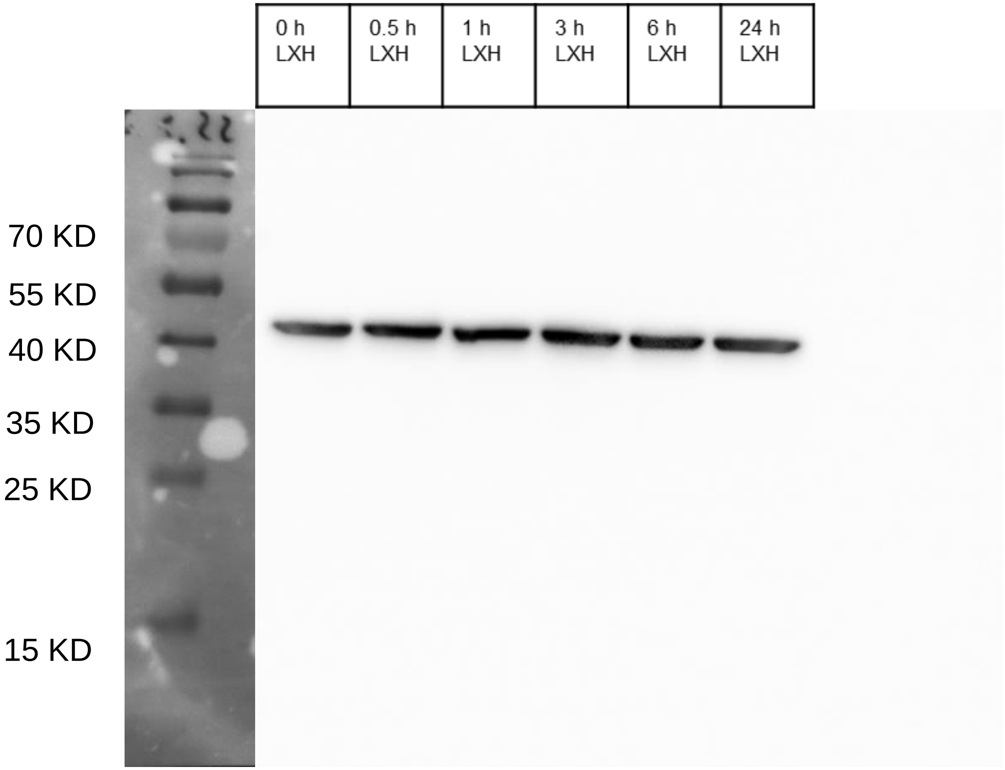

pEGFR (Panc02 cells + 1 µM LXH-254 22.03.2022)

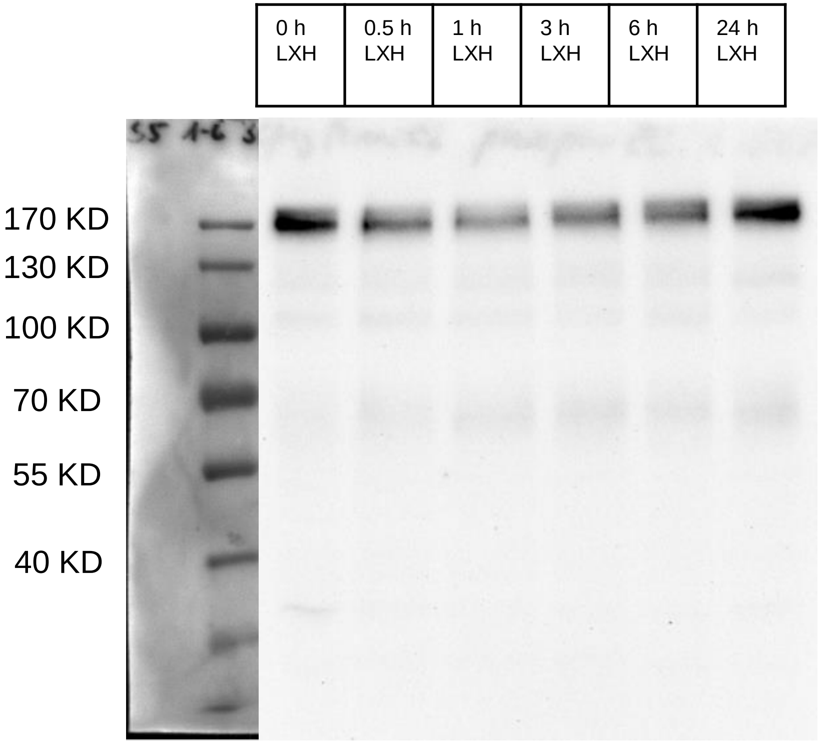

EGFR (Panc02 cells + 1 µM LXH-254 21.06.2022)

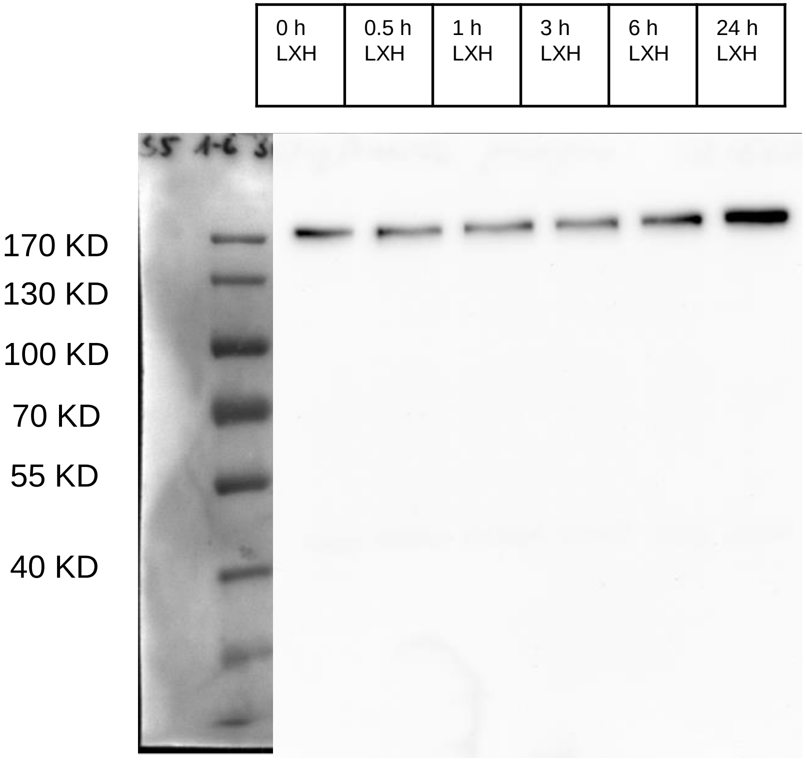

β-actin (pEGFR und EGFR_Panc02 cells + 1 µM LXH-254)
22.06.22

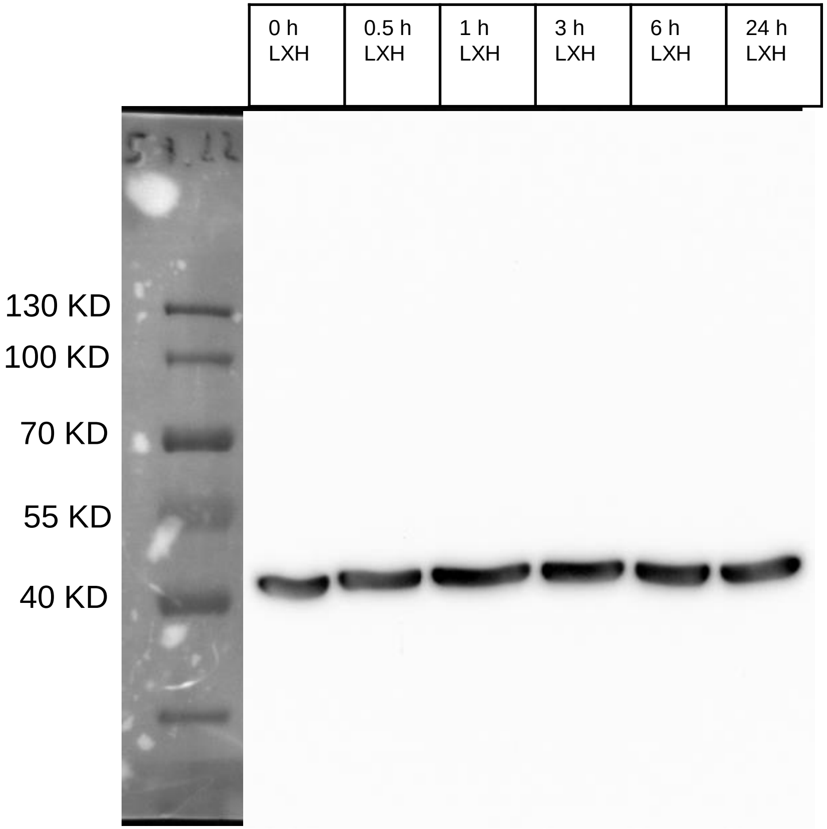

pMEK (Panc02 cells + 1 µM LXH- 254 21.06.2022)

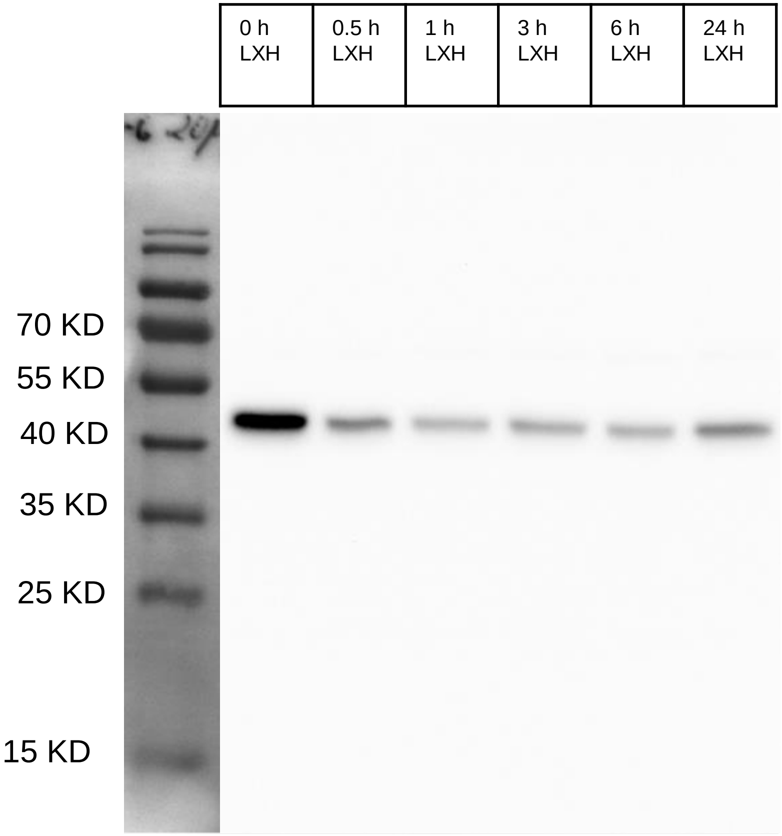

MEK (Panc02 cells + 1µM LXH-254 23.06.2022)

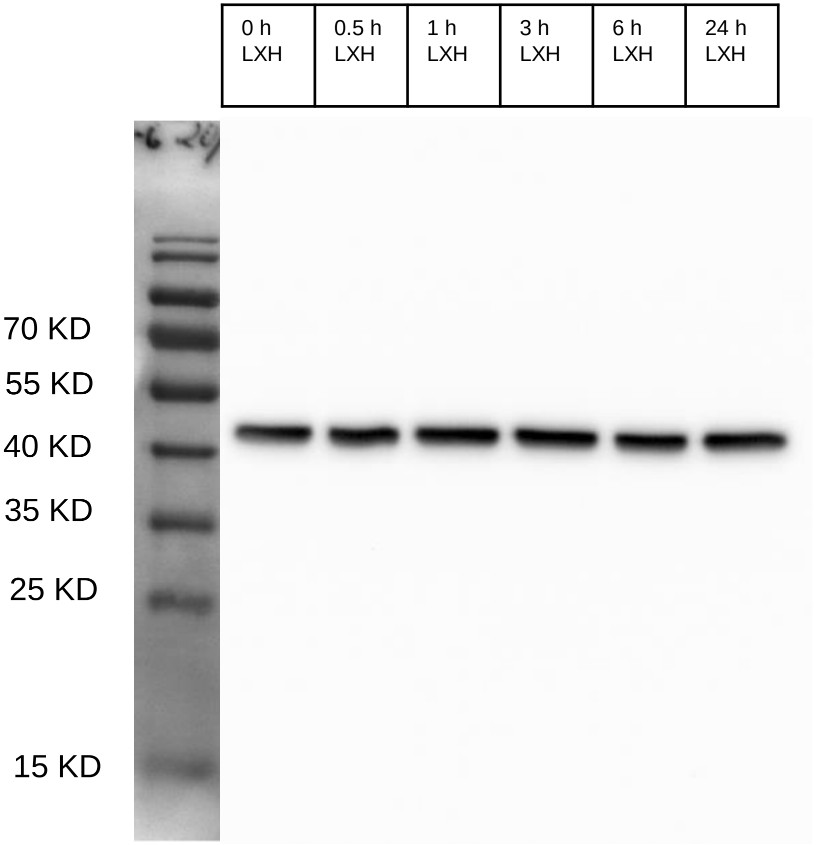

pERK (Panc02 cells + 1 µM LXH-254 21.06.2022)

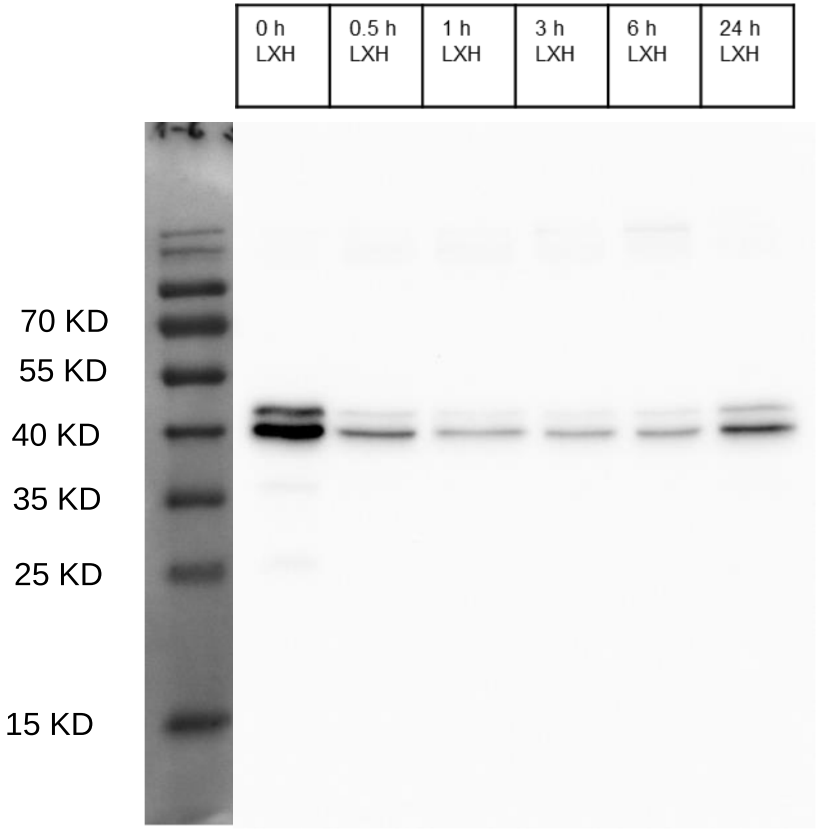

ERK (Panc02 cells + 1 µM LXH-254 23.06.2022)

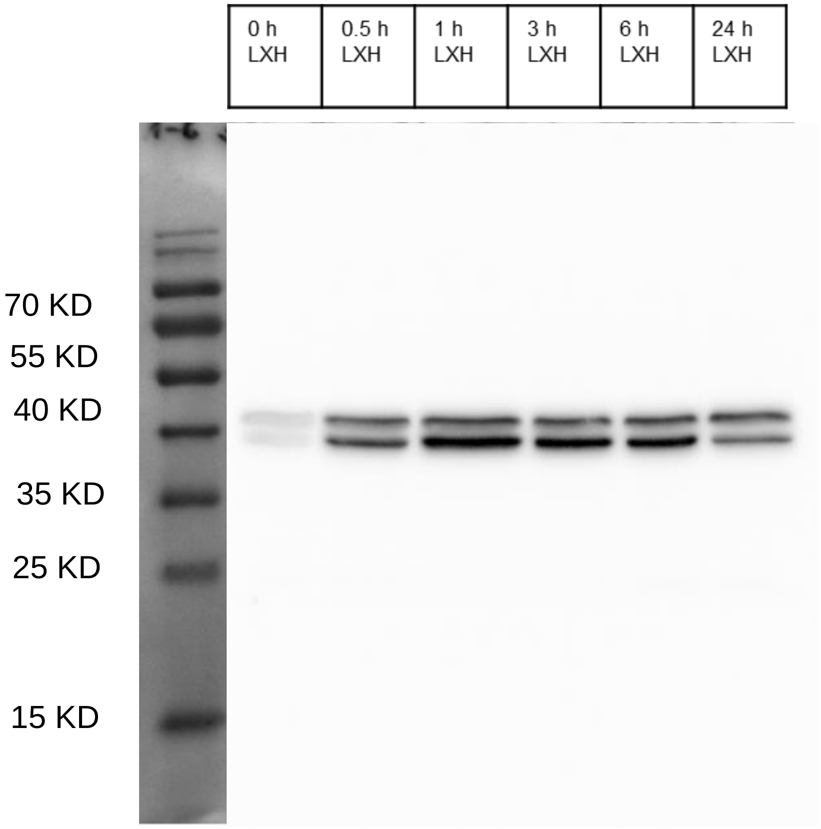

pEGFR (Panc02 cells + 1 µM LXH-254 27.07.2022)

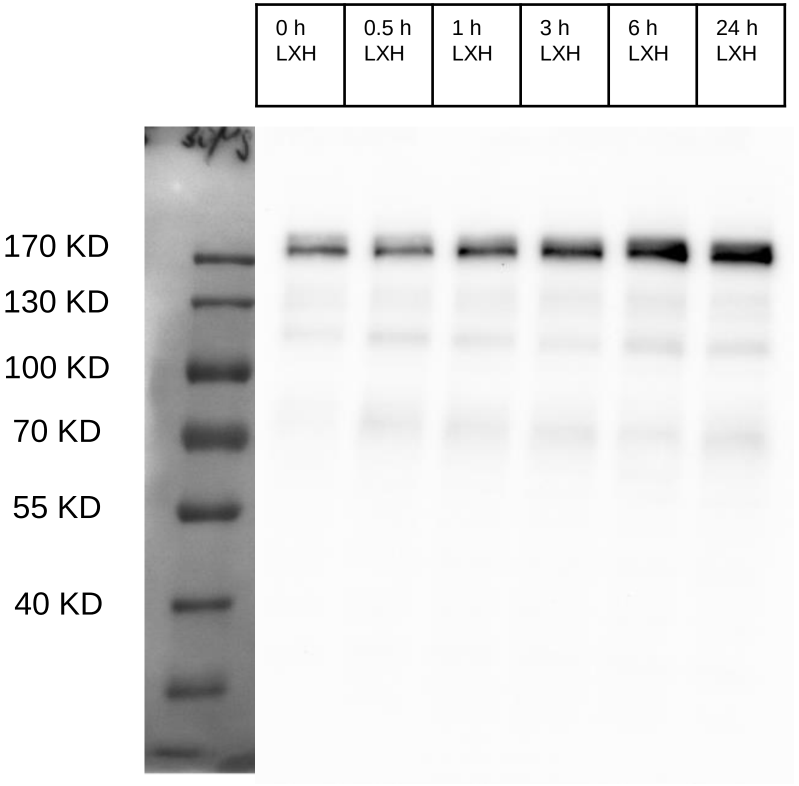

EGFR (Panc02 cells + 1 µM LXH-254 28.07.2022)

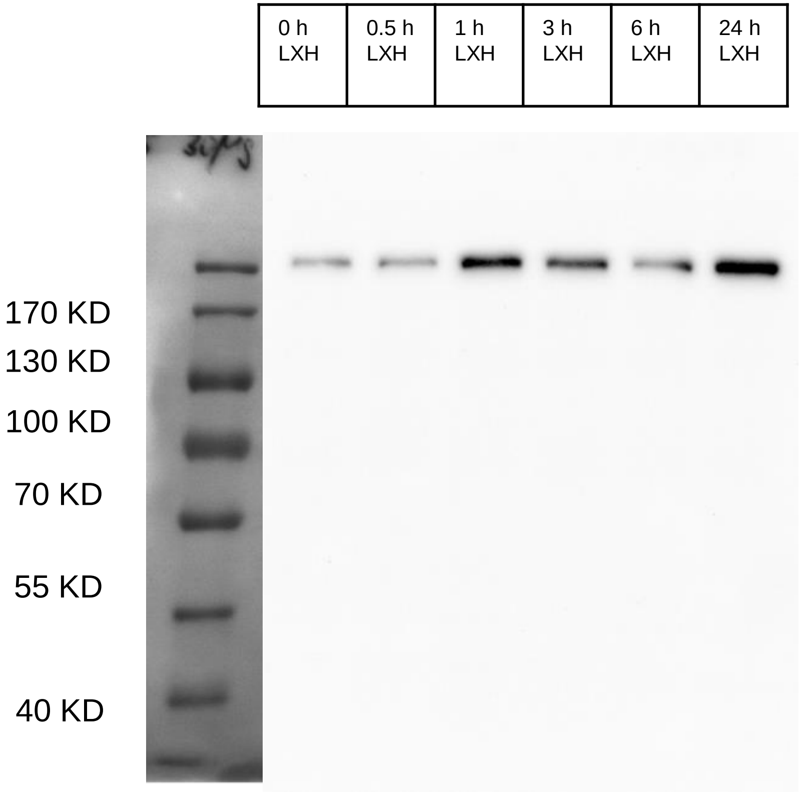

β-actin (pEGFR und EGFR_Panc02 cells + 1 µM LXH-254)
29.07.22

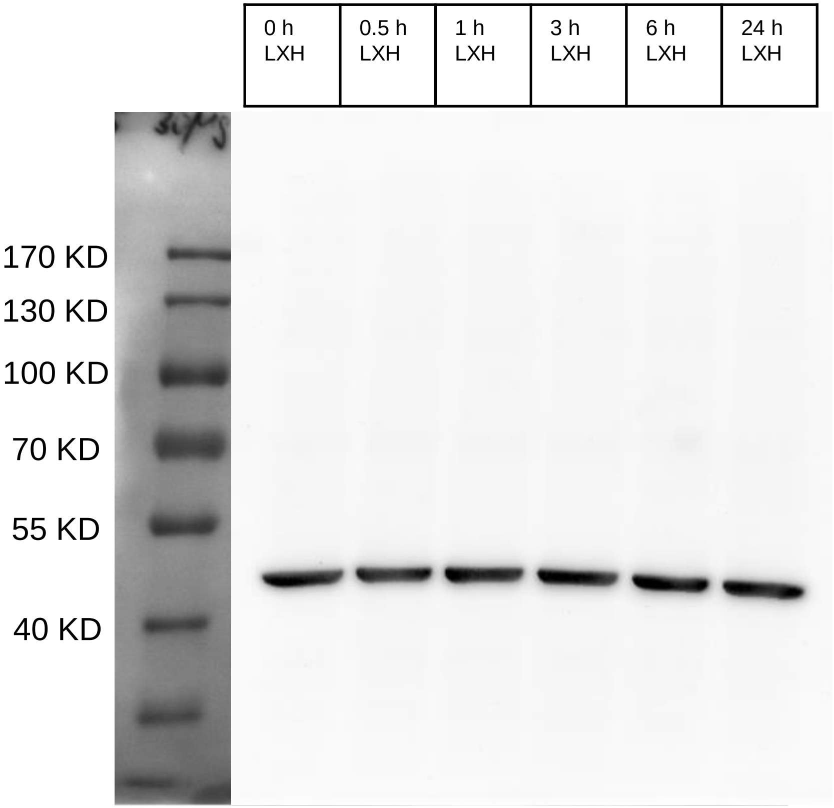

pMEK (Panc02 cells + 1 µM LXH- 254 27.07.2022)

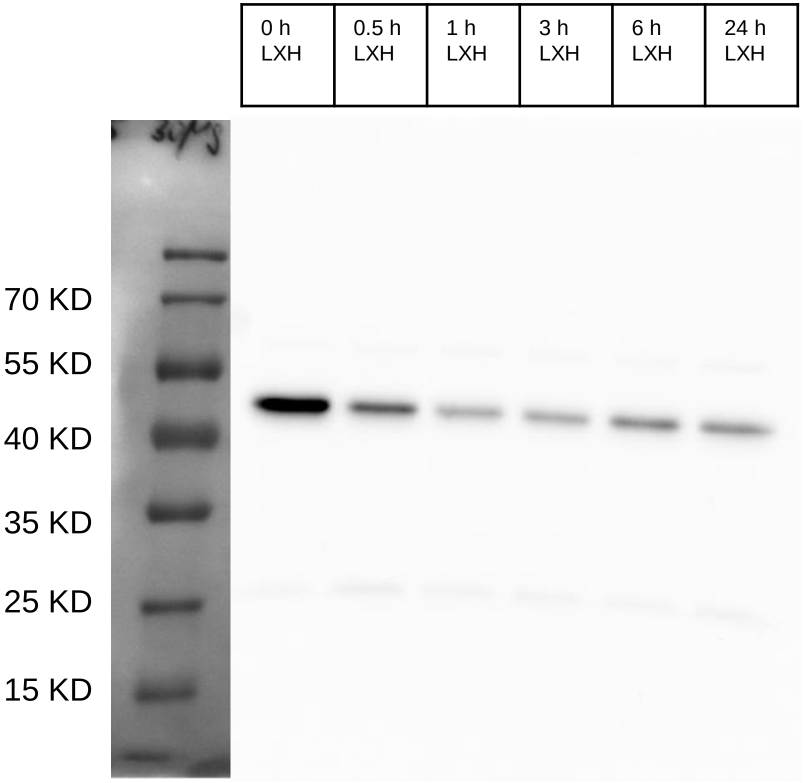

MEK (Panc02 cells + 1µM LXH-254 28.07.2022)

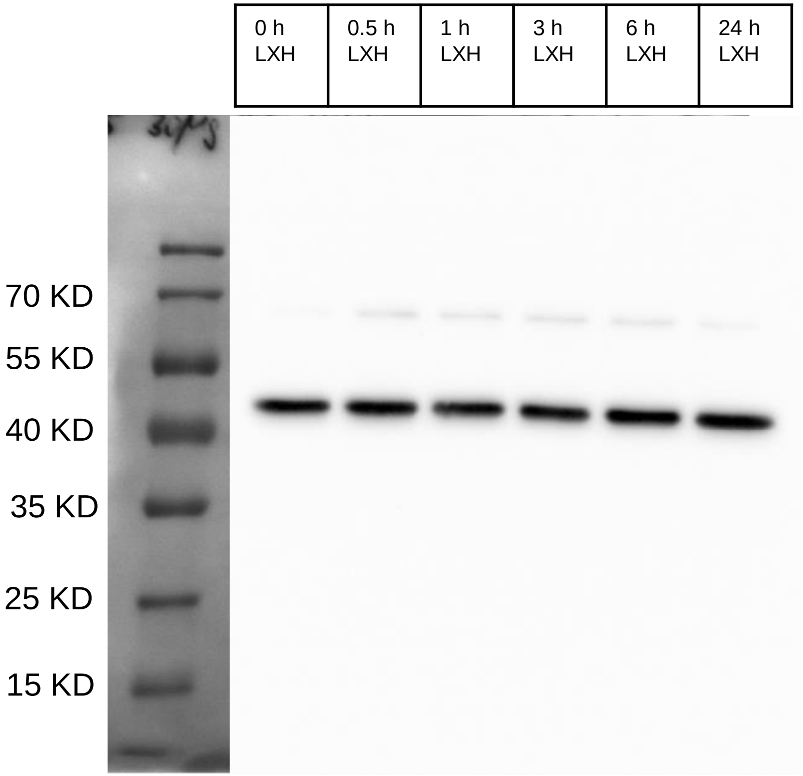

β-actin (pMEK/MEK Panc02 cells + 1µM LXH-254 29.07.2022)

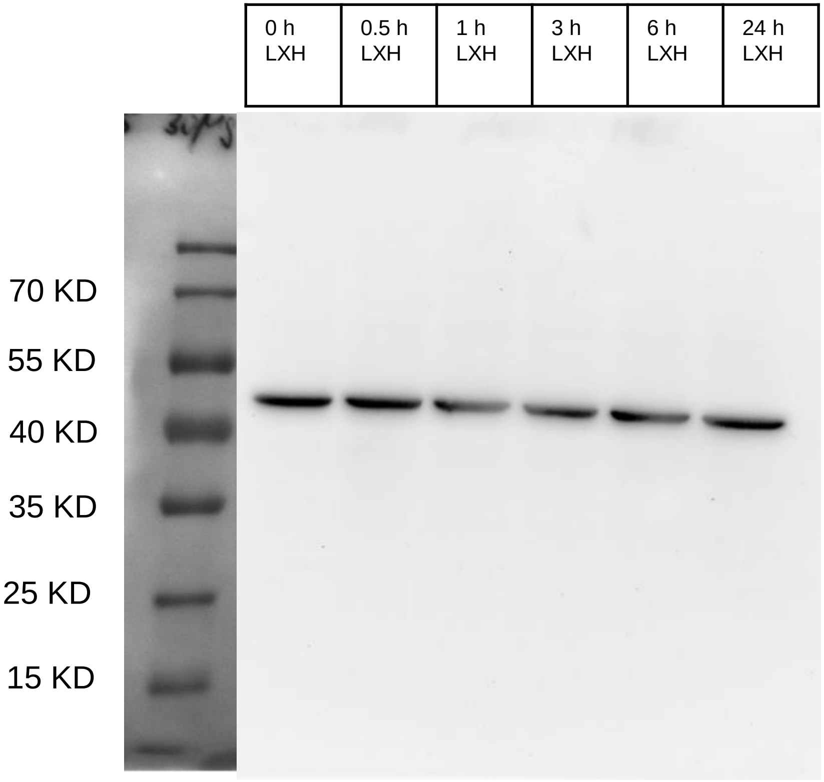

pERK (Panc02 cells + 1 µM LXH-254 27.07.2022)

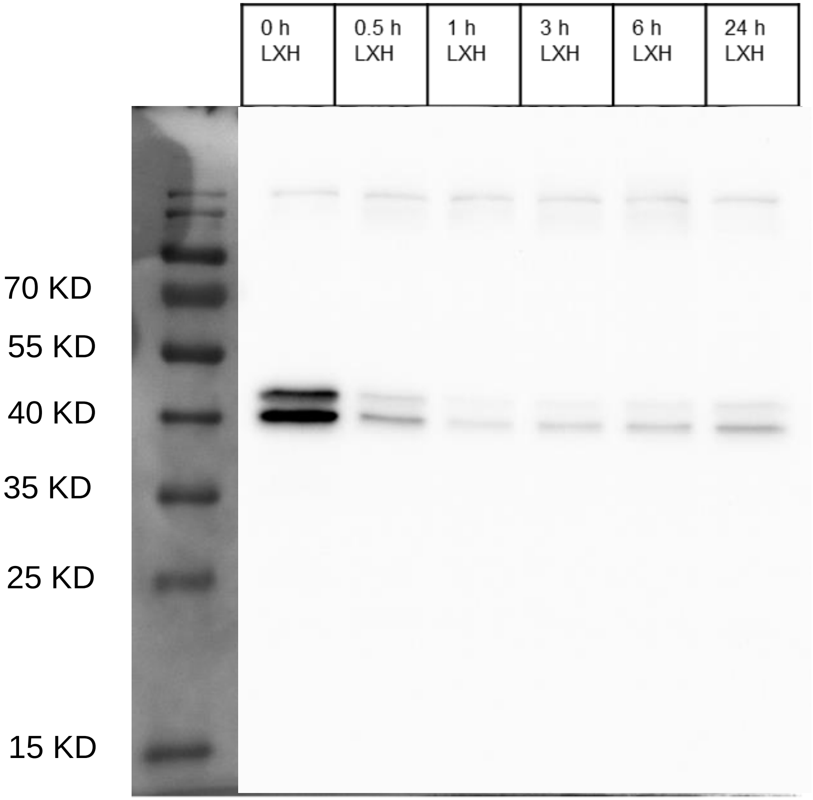

ERK (Panc02 cells + 1 µM LXH-254 28.07.2022)

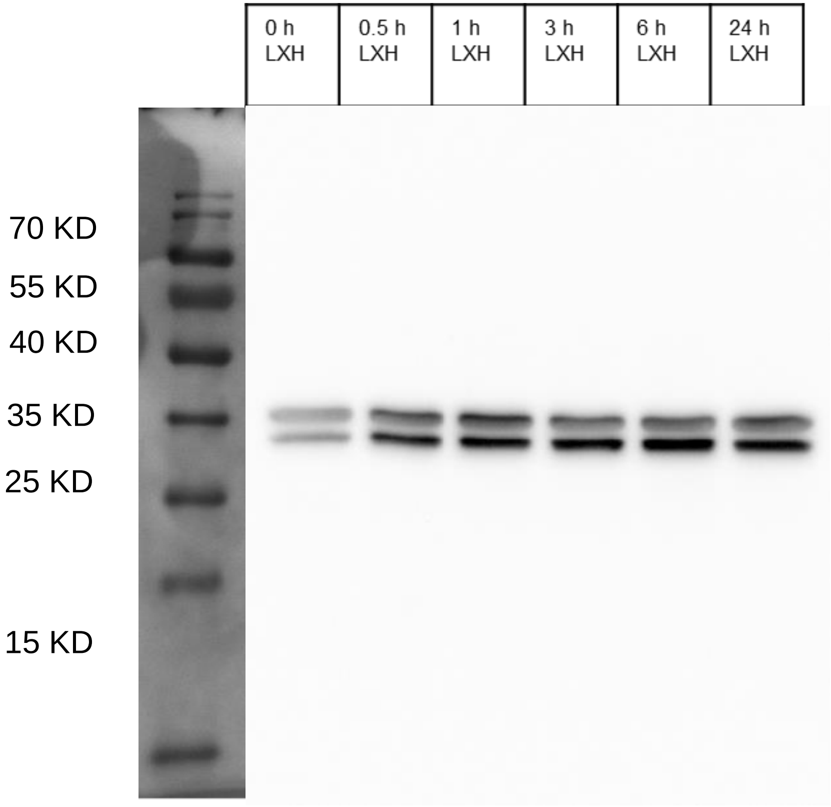

β-actin (pERK/ERK, Panc02-Zellen + 1 µM LXH-254 29.07.2022)

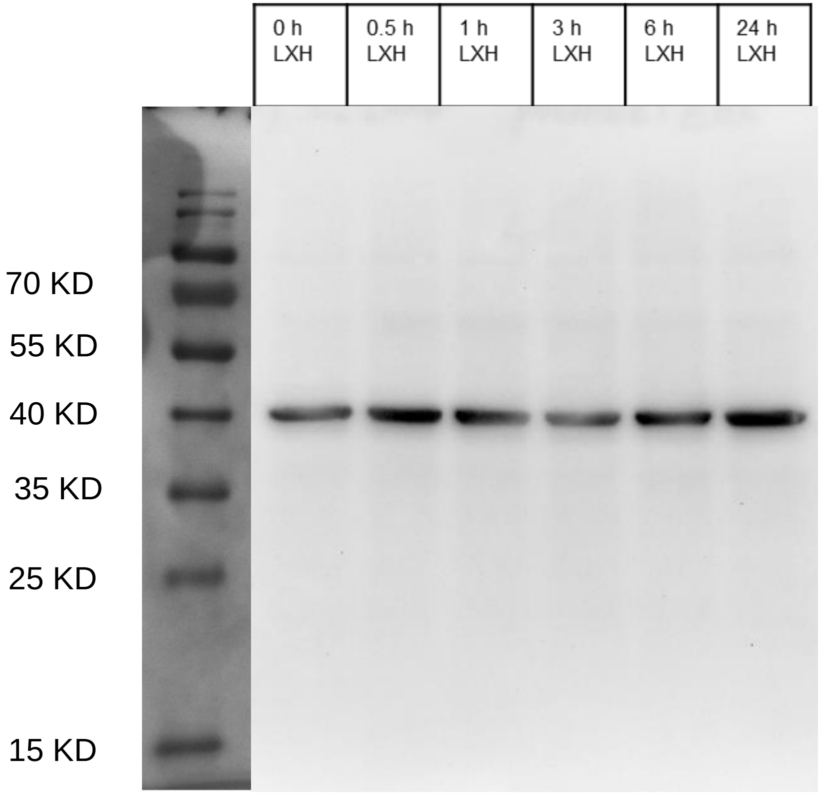

pEGFR (Panc02 cells + 1 µM LXH-254 17.08.2022)

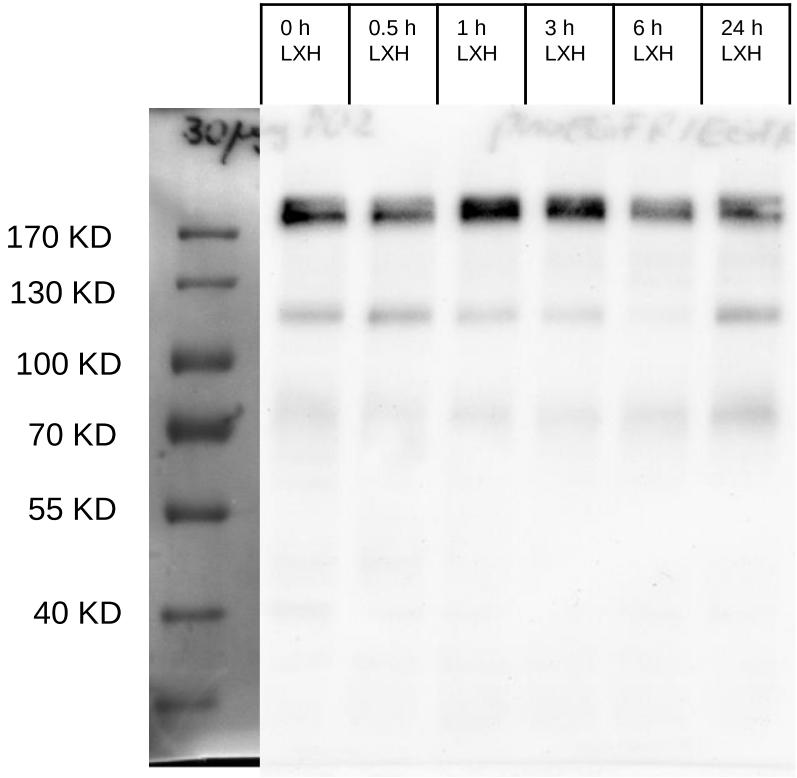

EGFR (Panc02 cells + 1 µM LXH-254 18.08.2022)

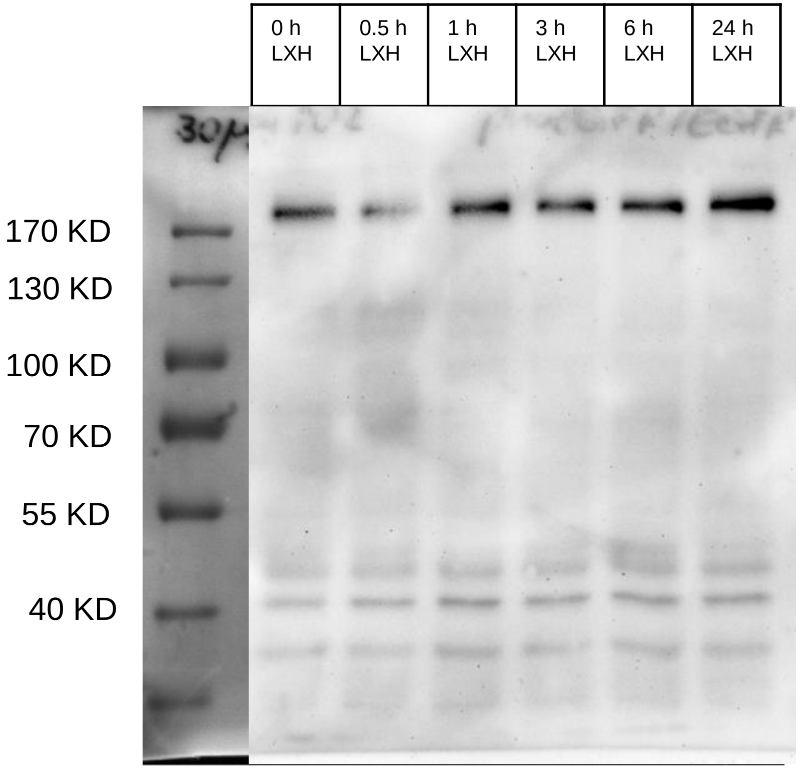

β-actin (pEGFR und EGFR_Panc02 cells + 1 µM LXH-254)
22.08.22

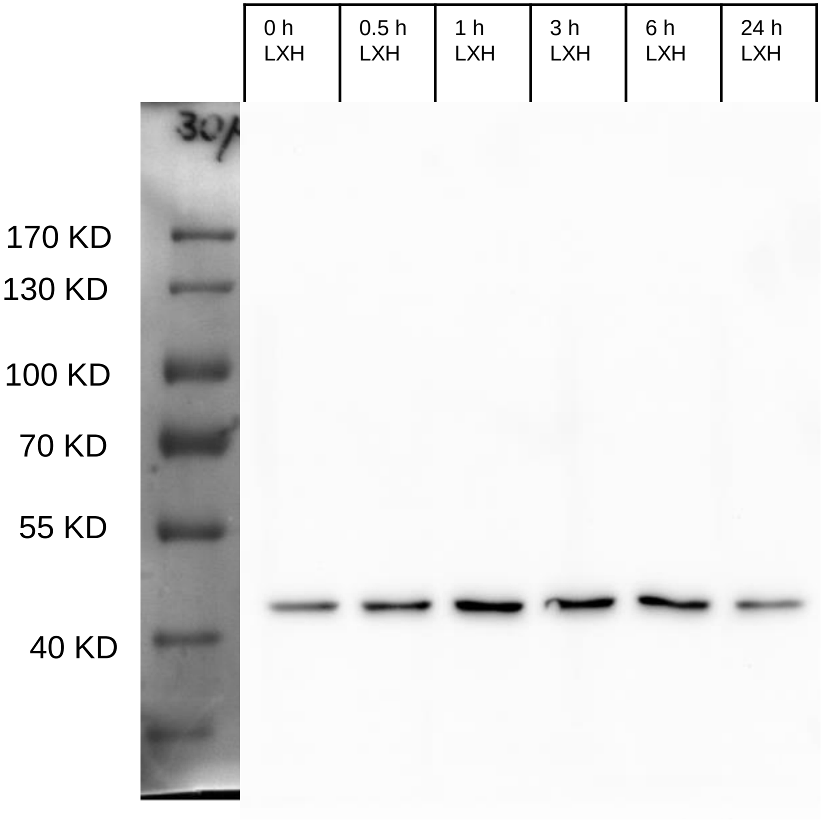

pMEK (Panc02 cells + 1 µM LXH- 254 17.08.2022)

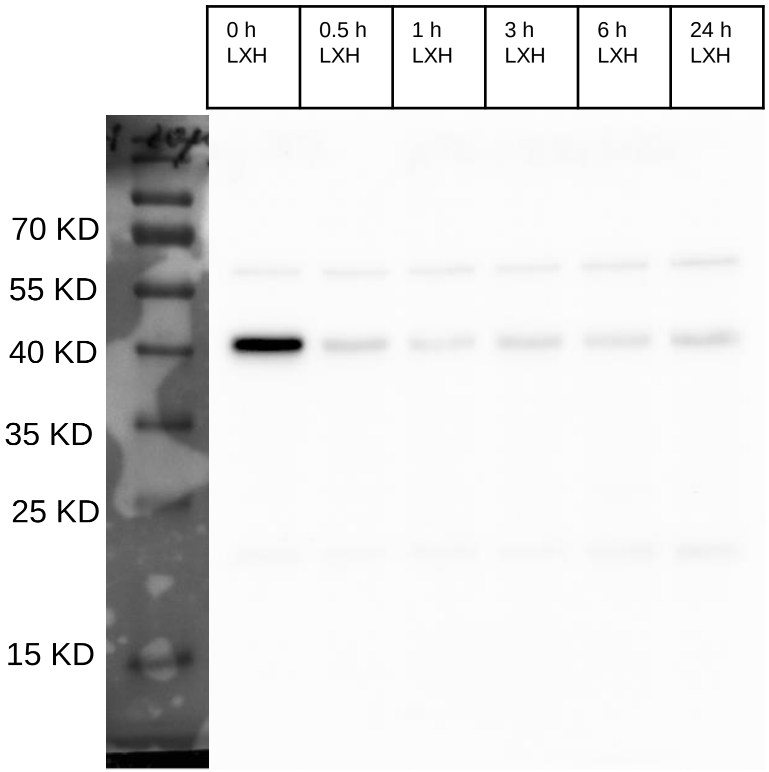

MEK (Panc02 cells + 1µM LXH-254 18.08.2022)

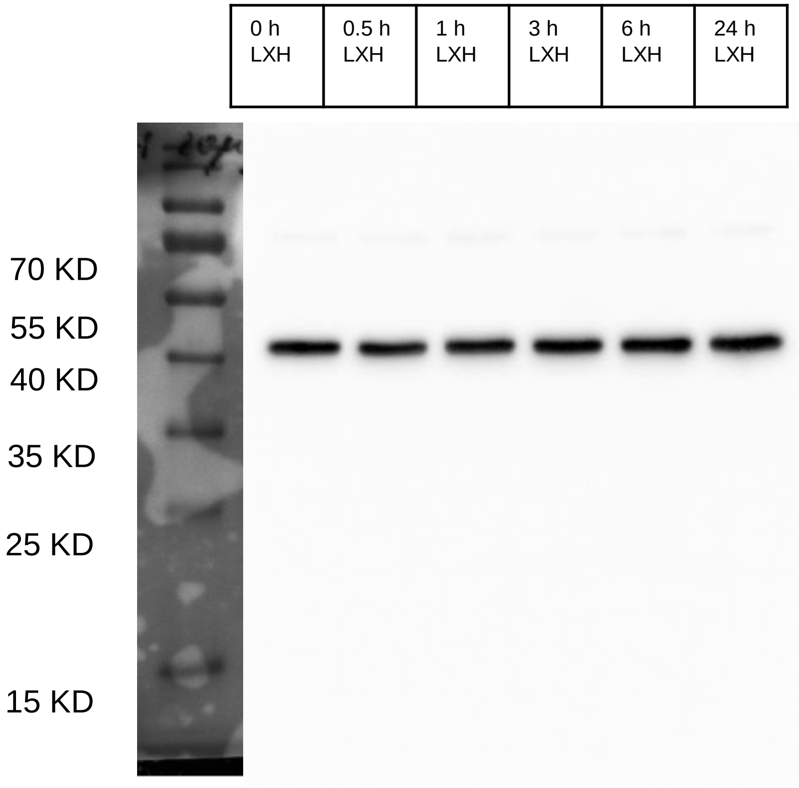

β-actin (pMEK/MEK Panc02 cells + 1µM LXH-254 22.08.2022)

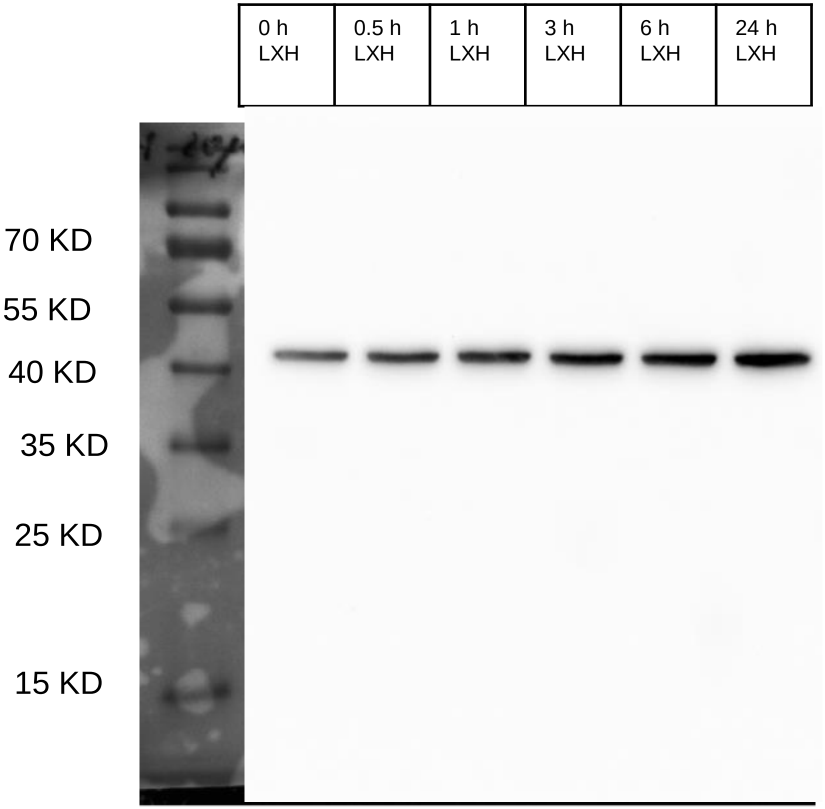

pEGFR (Panc02 cells + 1 µM Erlotinib 21.06.2022)

EGFR (Panc02 cells + 1 µM Erlotinib 23.06.2022)

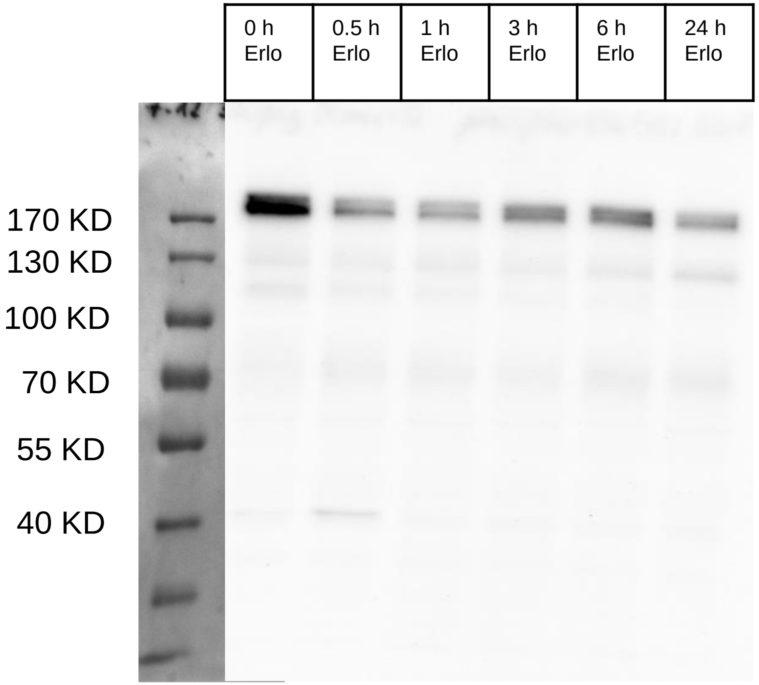

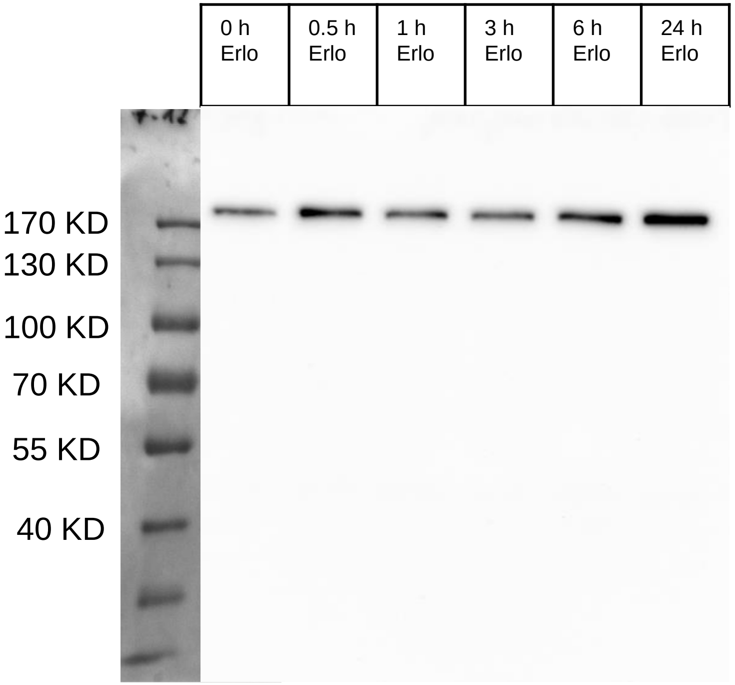

β-actin (pEGFR/ EGFR, Panc02 cells + 1 µM Erlotinib 24.06.2022)

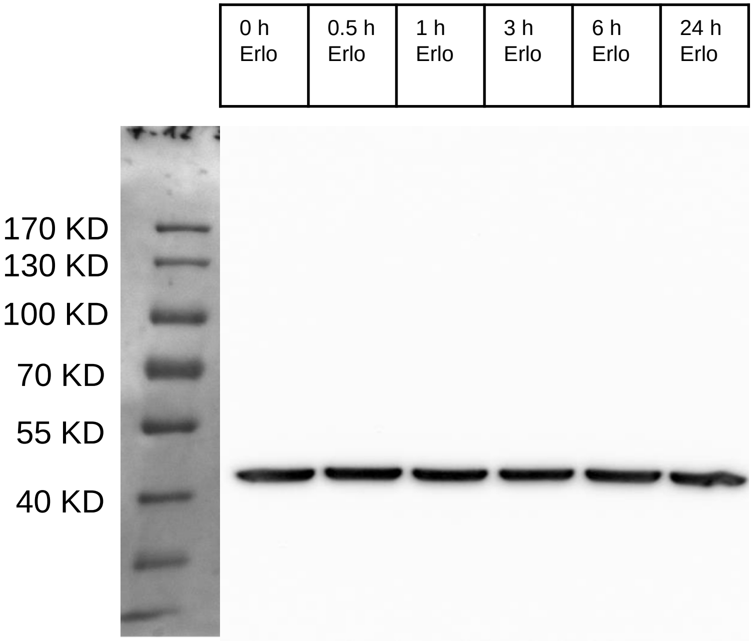

pMek (Panc02 cells + 1 µM Erlotinib 21.06.2022)

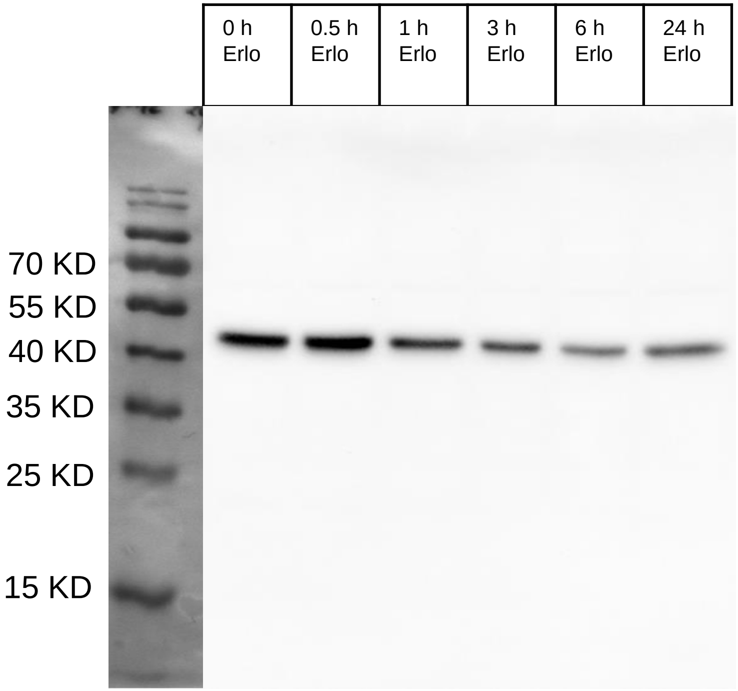

Mek (Panc02 cells + 1 µM Erlotinib 23.06.2022)

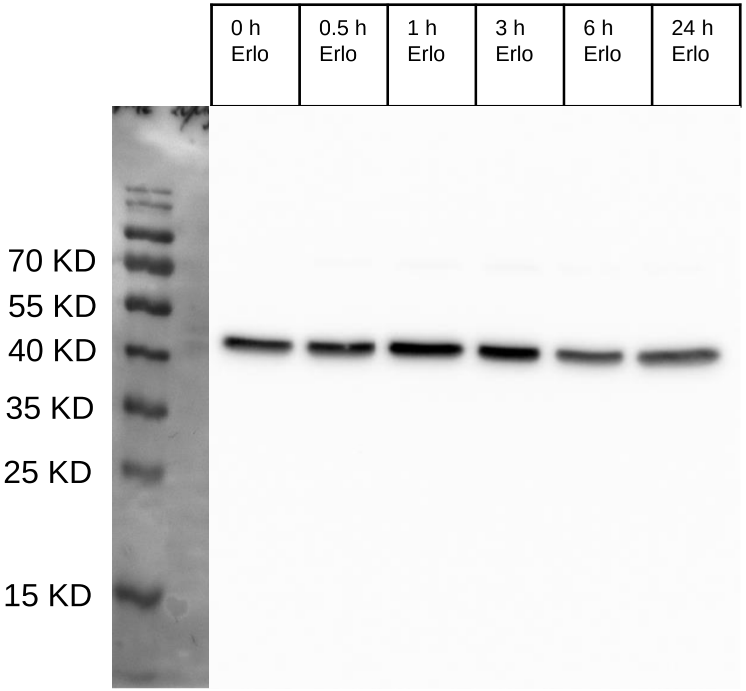

β-actin (pMEK/MEK, Panc02 cells + 1 µM Erlotinib 24.06.2022)

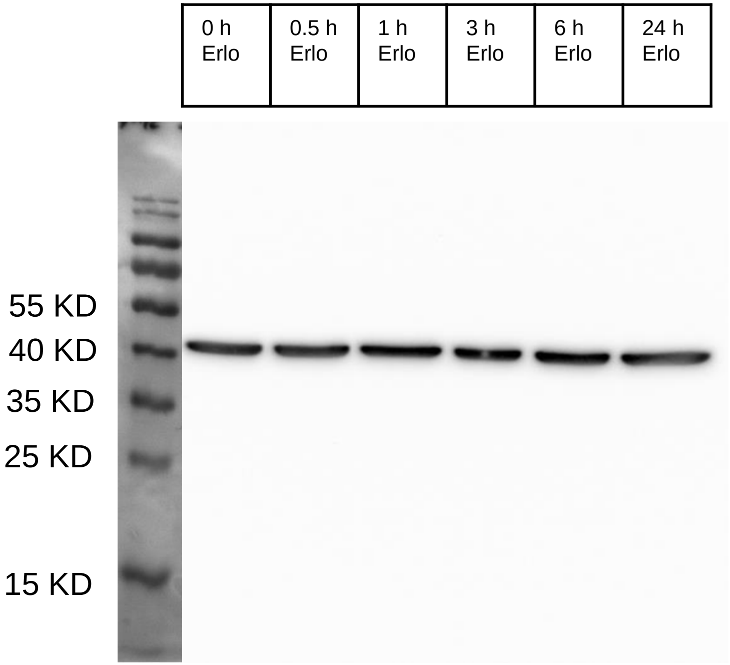

pERK (Panc02 cells + 1 µM Erlotinib 21.06.2022)

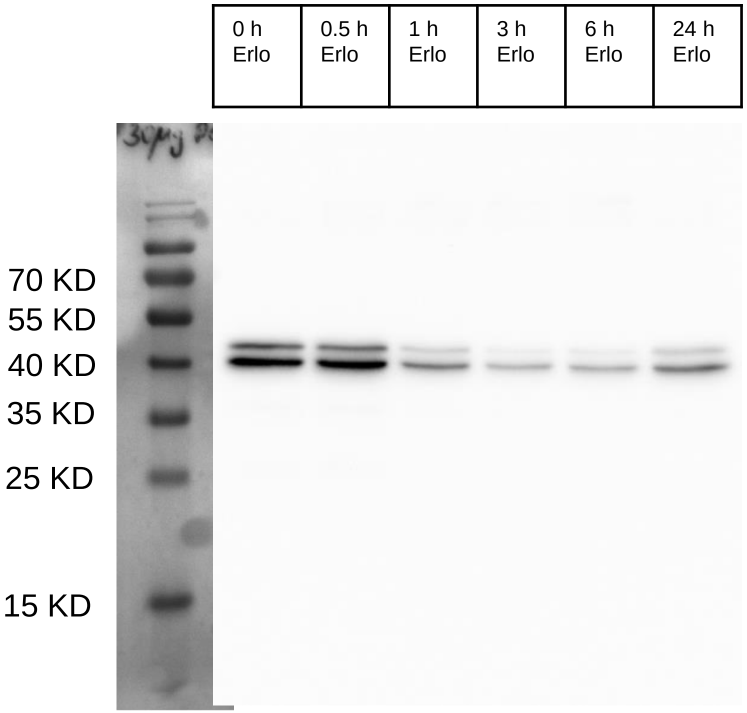

ERK (Panc02 cells + 1 µM Erlotinib 23.06.2022)

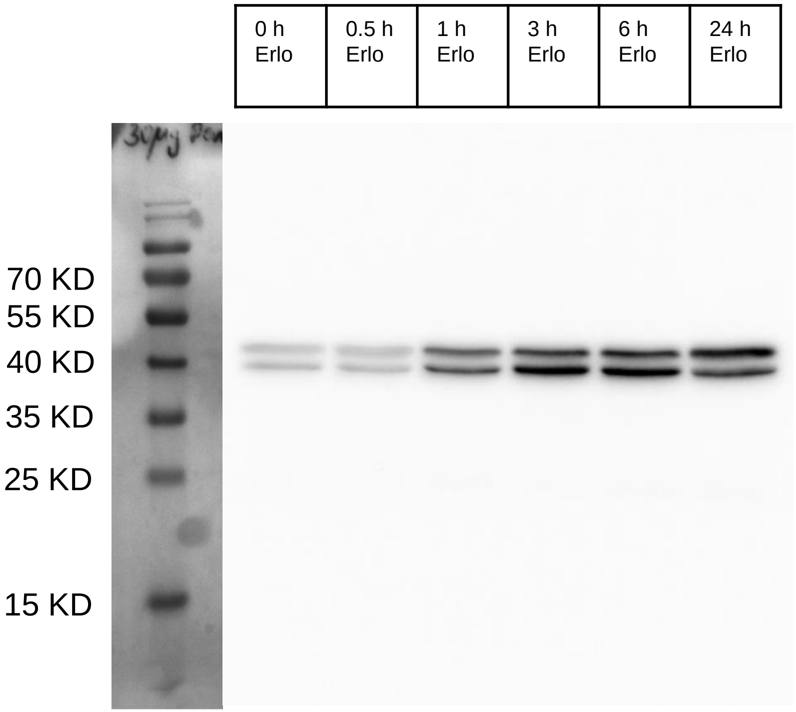

β-actin (pERK/ERK Panc02 cells + 1 µM Erlotinib 24.06.2022)

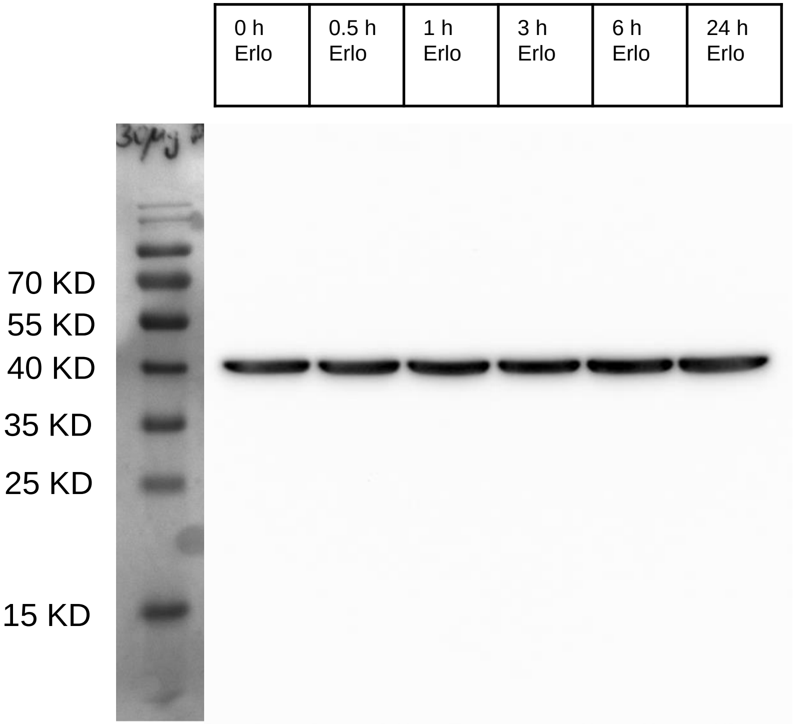

pEGFR (Panc02 cells + 1 µM Erlotinib 27.07.2022)

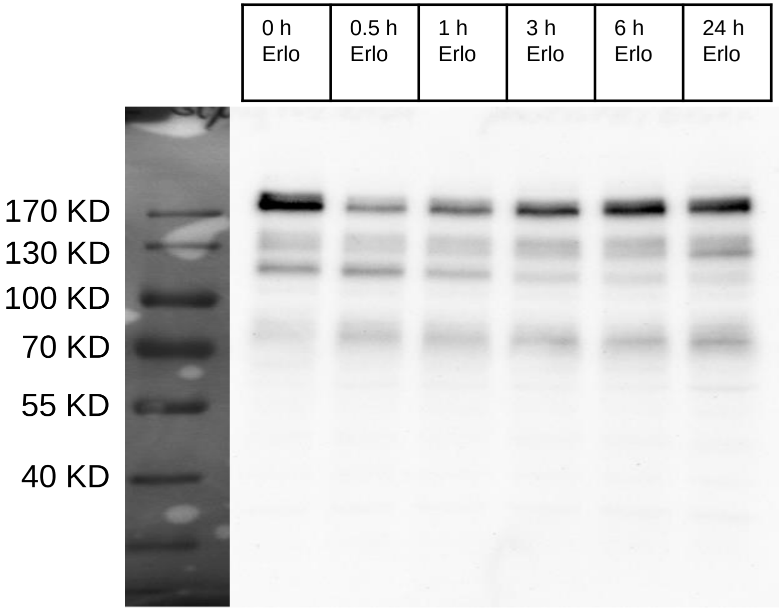

EGFR (Panc02 cells + 1 µM Erlotinib 28.07.2022)

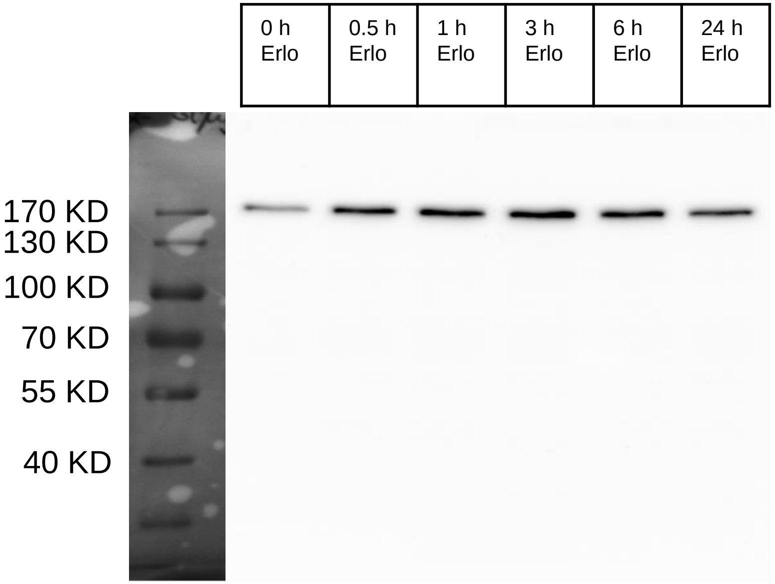

β-actin (pEGFR/ EGFR, Panc02 cells + 1 µM Erlotinib 29.07.2022)

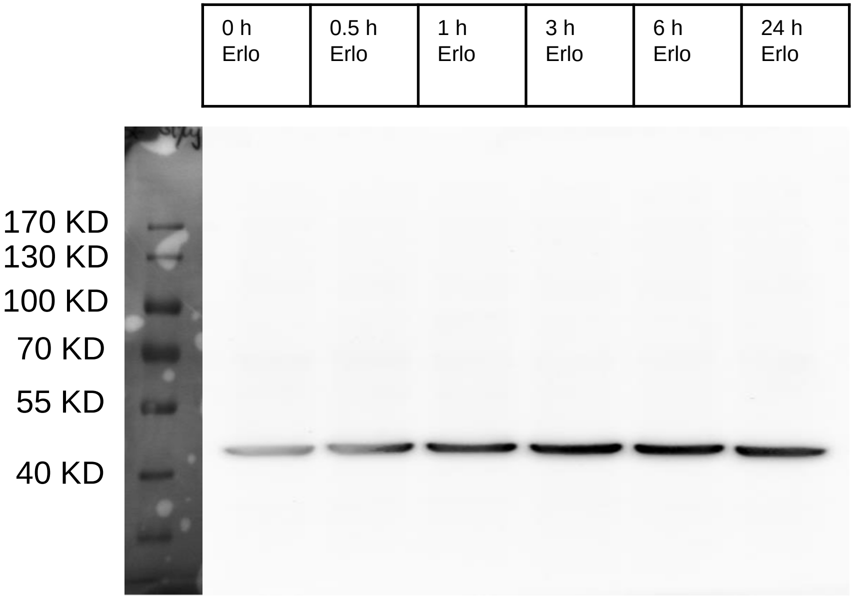

pMEK (Panc02 cells + 1 µM Erlotinib 27.07.2022)

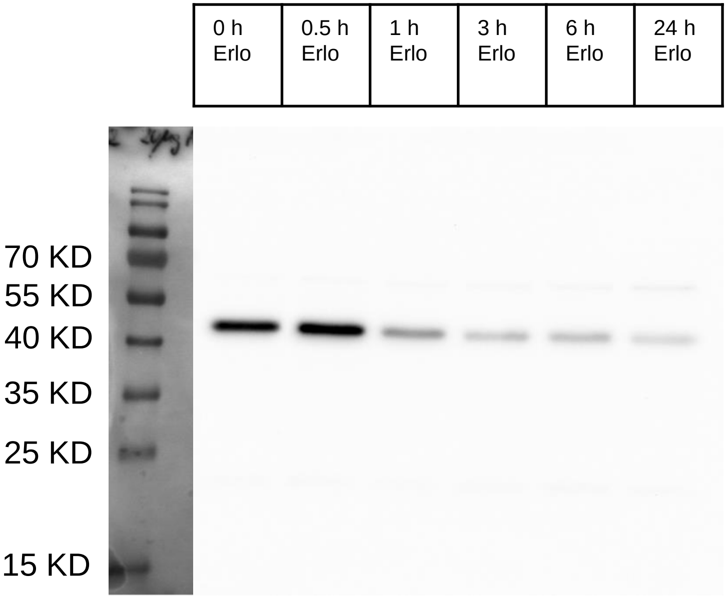

MEK (Panc02 cells + 1 µM Erlotinib 28.07.2022)

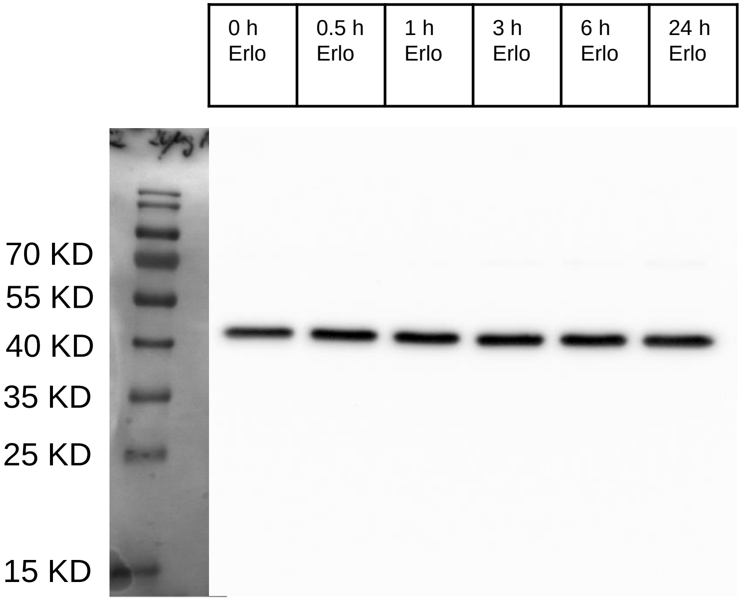

β-actin (pMEK/MEK, Panc02 cells + 1 µM Erlotinib 29.07.2022)

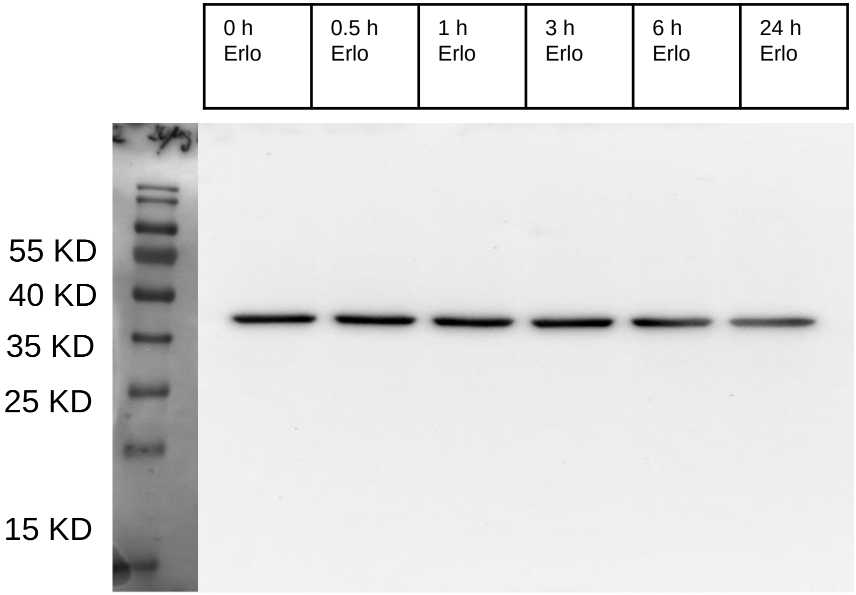

pERK (Panc02 cells + 1 µM Erlotinib 27.07.2022)

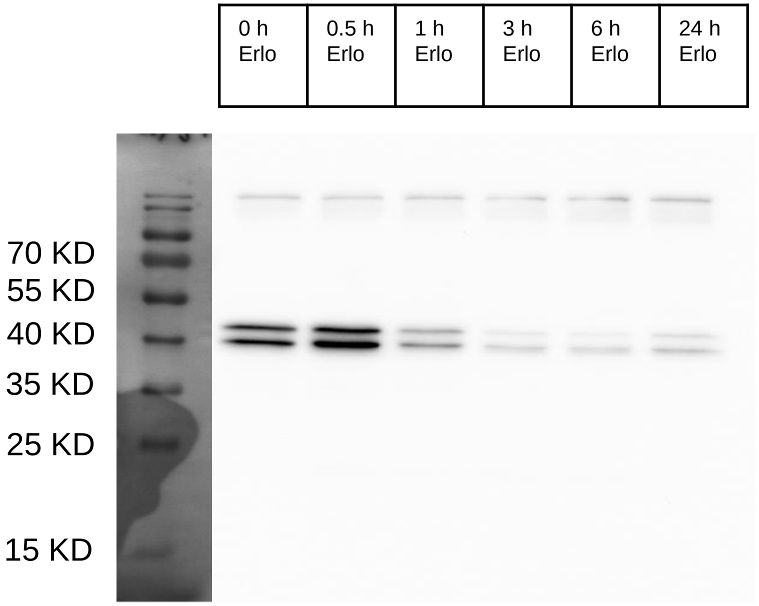

ERK (Panc02 cells + 1 µM Erlotinib 28.07.2022)

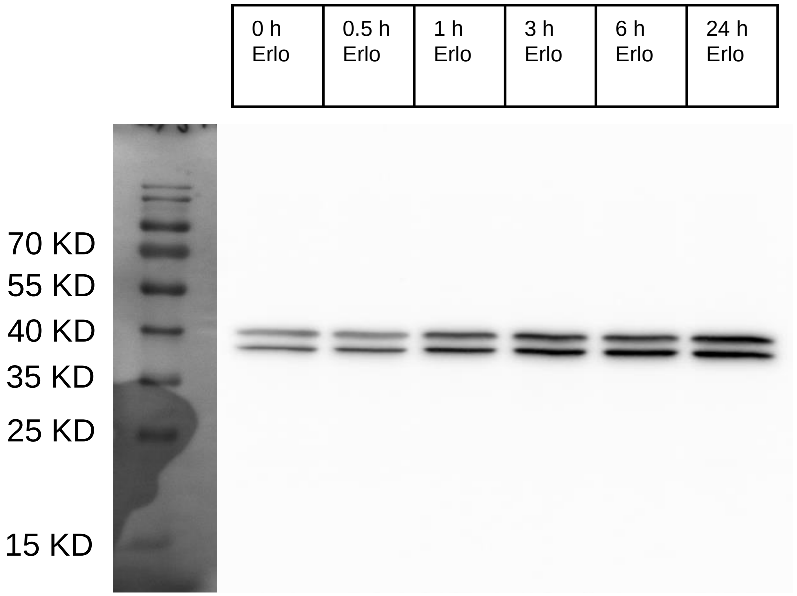

β-actin (pERK/ERK Panc02 cells + 1 µM Erlotinib 29.07.2022)

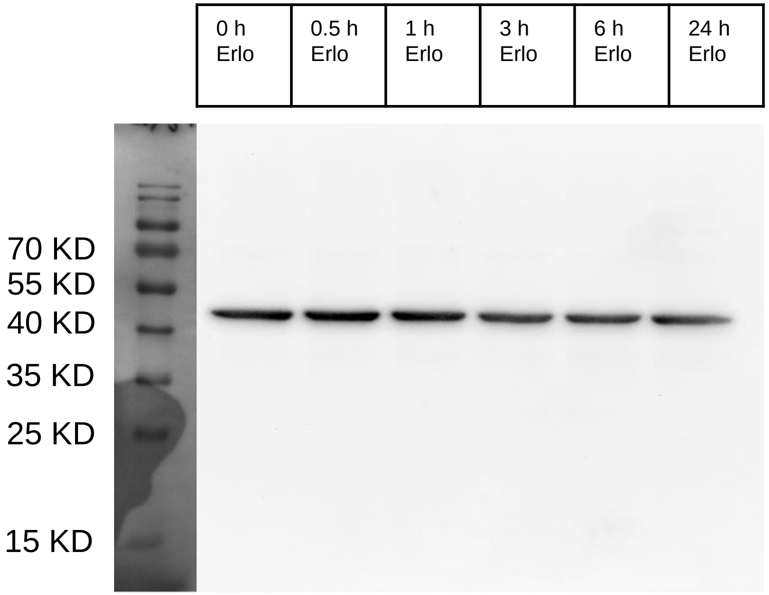

pEGFR (Panc02 cells + 1 µM Erlotinib 17.08.2022)

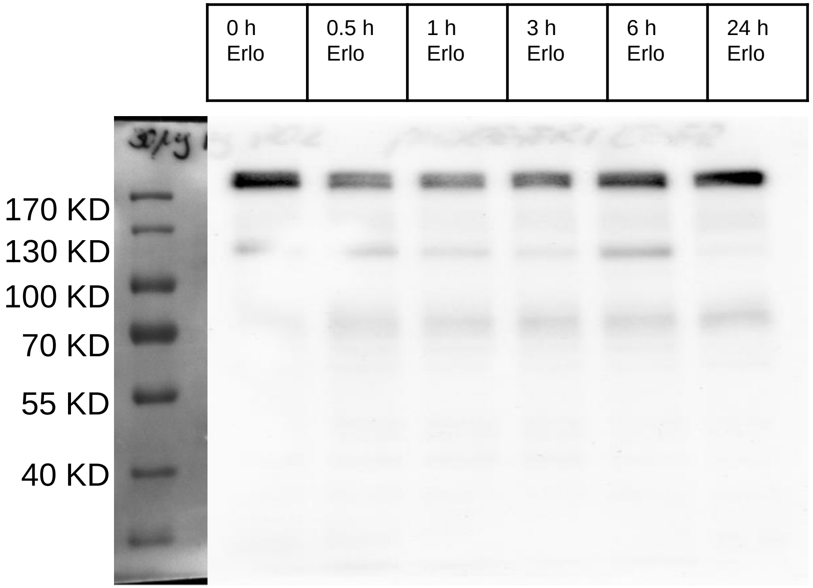

EGFR (Panc02 cells + 1 µM Erlotinib 18.08.2022)

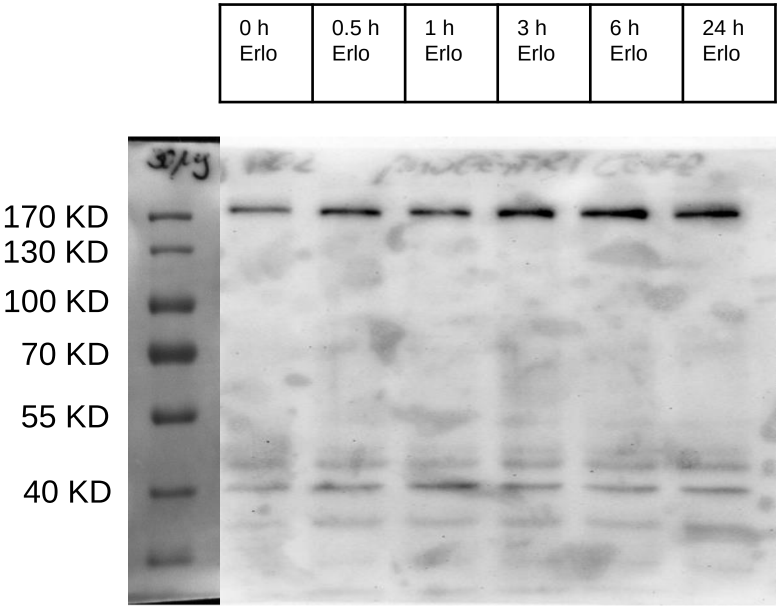

β-actin (pEGFR/ EGFR, Panc02 cells + 1 µM Erlotinib 22.08.2022)

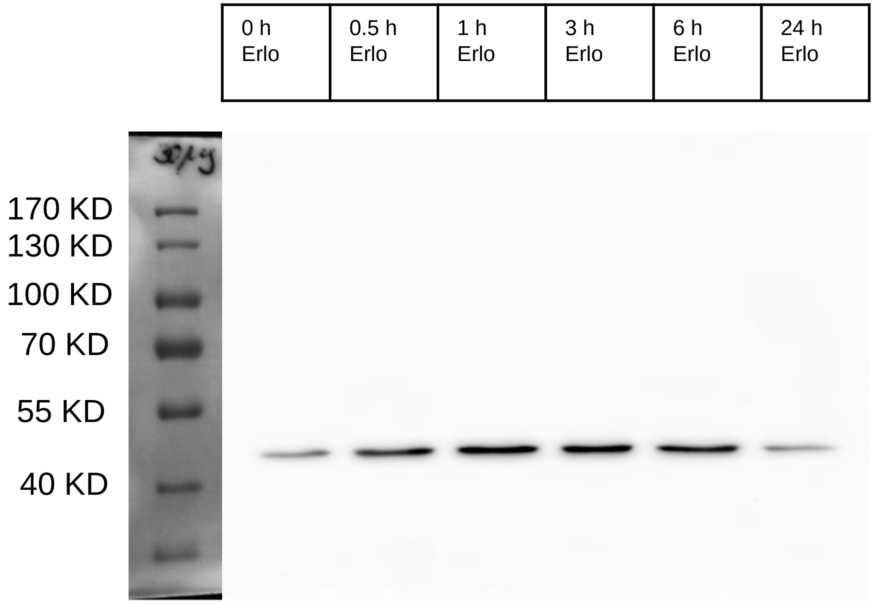

pMEK (Panc02 cells + 1 µM Erlotinib 17.08.2022)

MEK (Panc02 cells + 1 µM Erlotinib 18.08.2022)

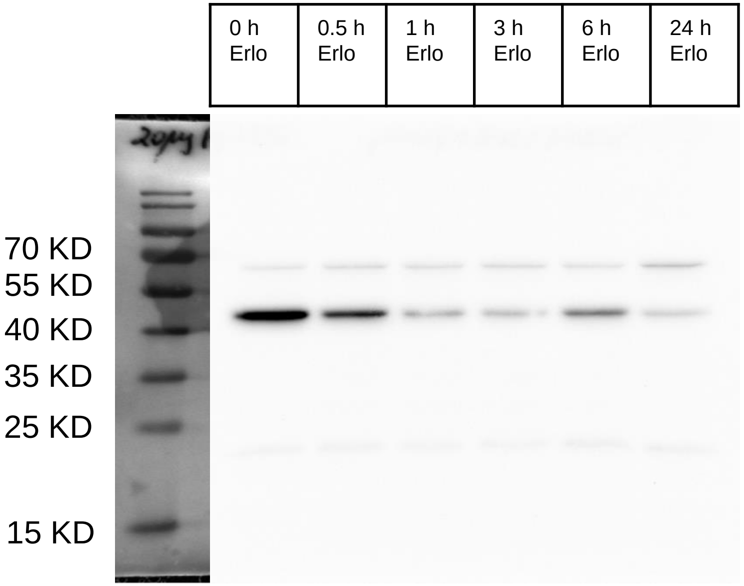

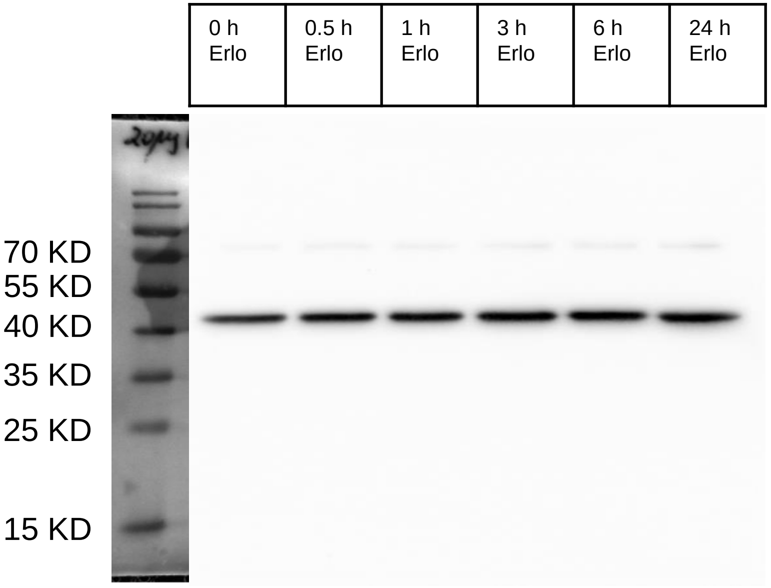

β-actin (pMEK/MEK, Panc02 cells + 1 µM Erlotinib 22.08.2022)

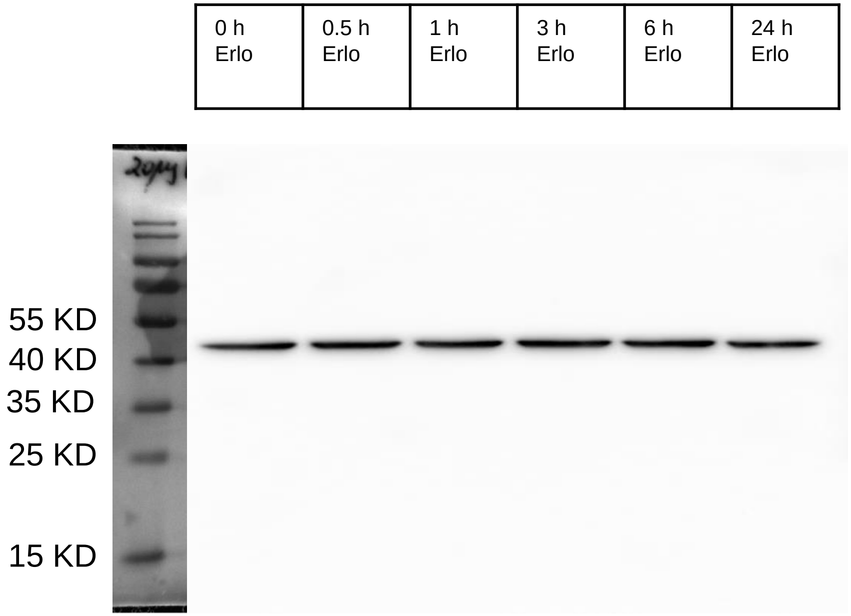

pERK (Panc02 cells + 1 µM Erlotinib 17.08.2022)

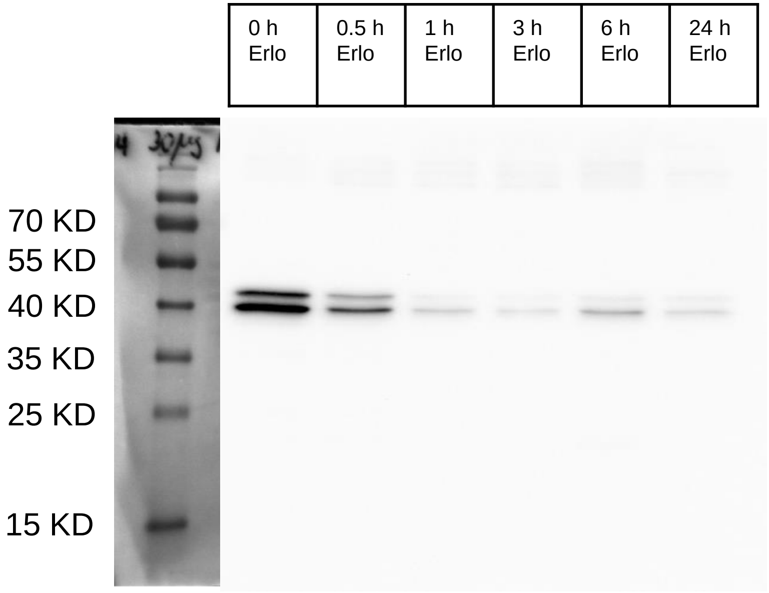

ERK (Panc02 cells + 1 µM Erlotinib 18.08.2022)

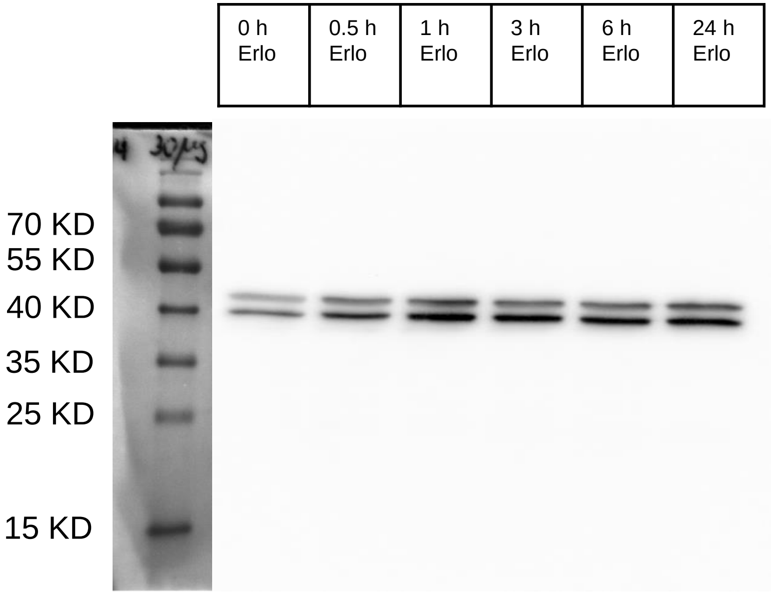

β-actin (pERK/ERK Panc02 cells + 1 μM Erlotinib 22.08.2022)

0 h Erlo	0.5 h Erlo	1 h Erlo	3 h Erlo	6 h Erlo	24 h Erlo

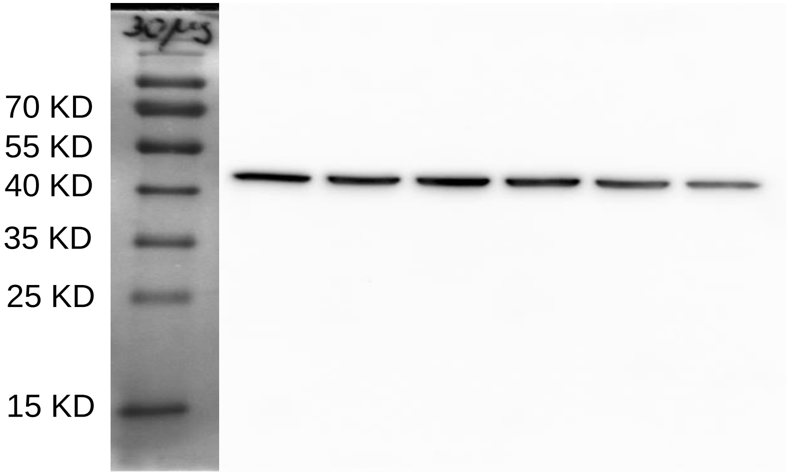

pEGFR (Panc02 cells + 1 µM Erlotinib 26.10.2022)

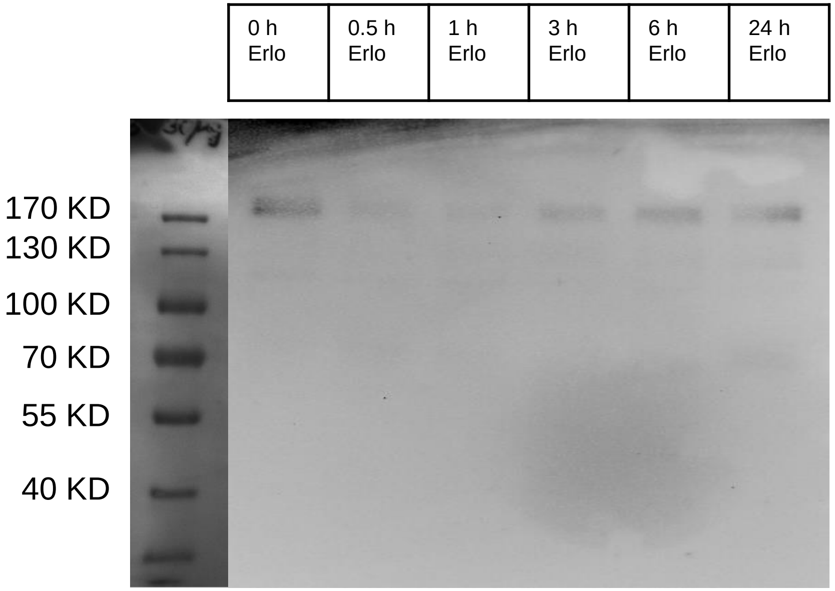

EGFR (Panc02 cells + 1 µM Erlotinib 27.10.2022)

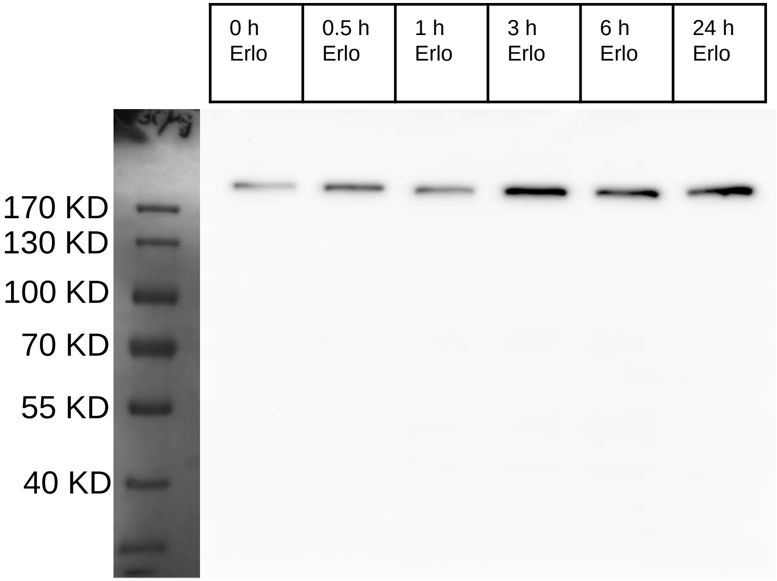

β-actin (pEGFR/ EGFR, Panc02 cells + 1 µM Erlotinib 28.10.2022)

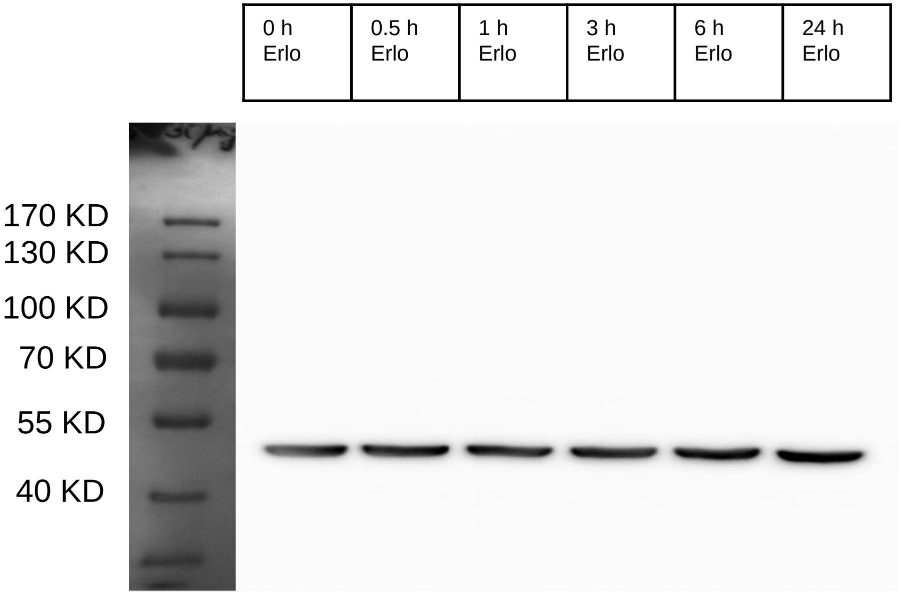

pMEK (Panc02 cells + 1 µM Erlotinib 26.10.2022)

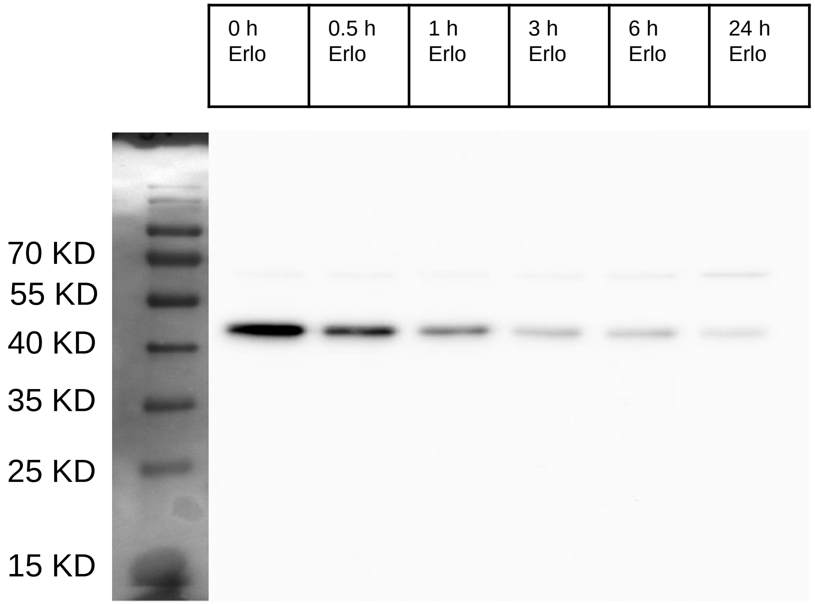

MEK (Panc02 cells + 1 µM Erlotinib 27.10.2022)

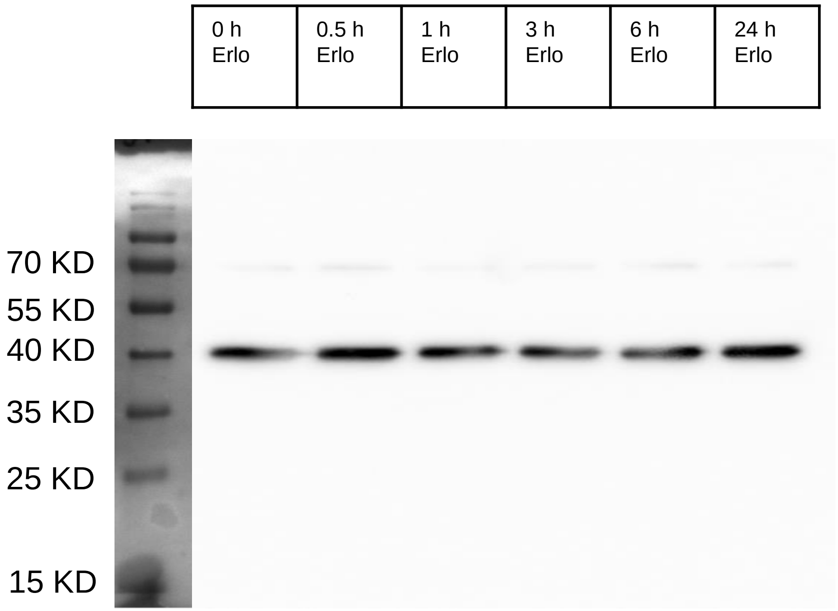

β-actin (pMEK/MEK, Panc02 cells + 1 µM Erlotinib 28.10.2022)

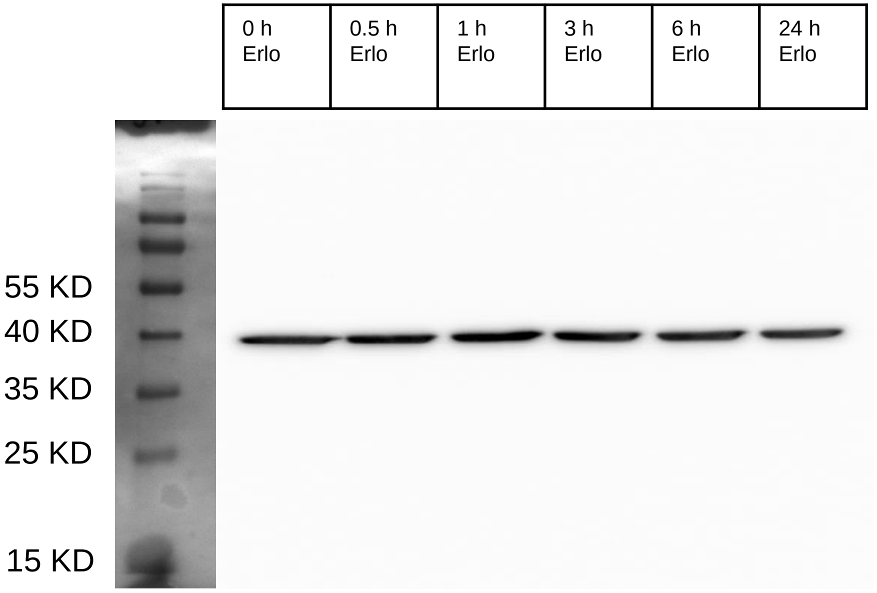

pERK (Panc02 cells + 1 µM Erlotinib 26.10.2022)

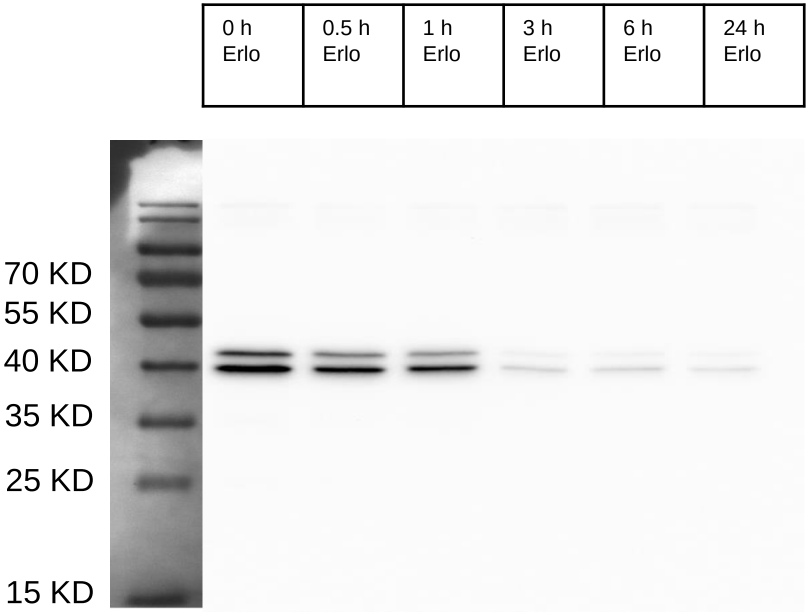

ERK (Panc02 cells + 1 µM Erlotinib 27.10.2022)

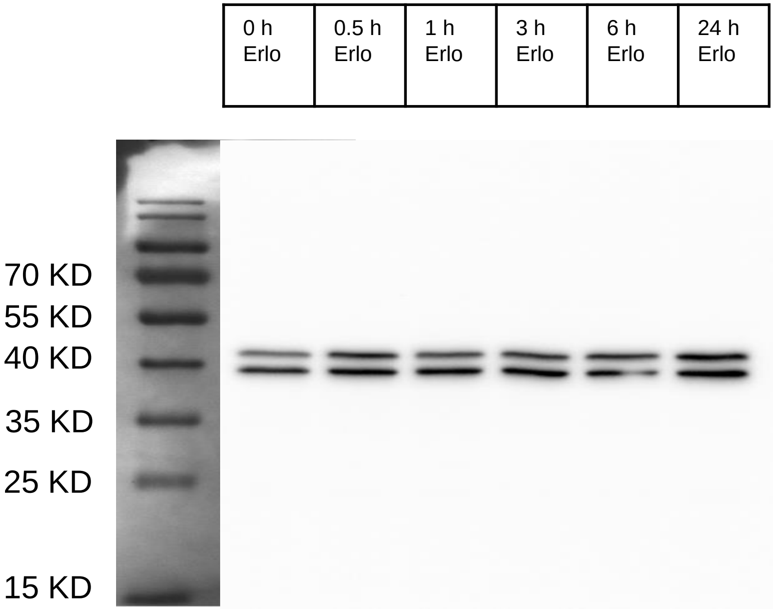

β-actin (pERK/ERK Panc02 cells + 1 µM Erlotinib 28.10.2022)

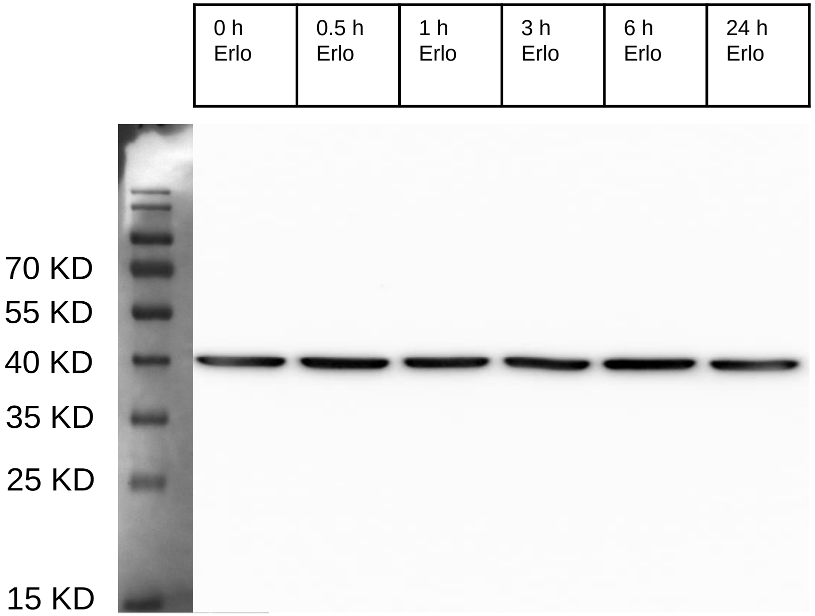

pEGFR: Panc02 cells: 05.01.2021

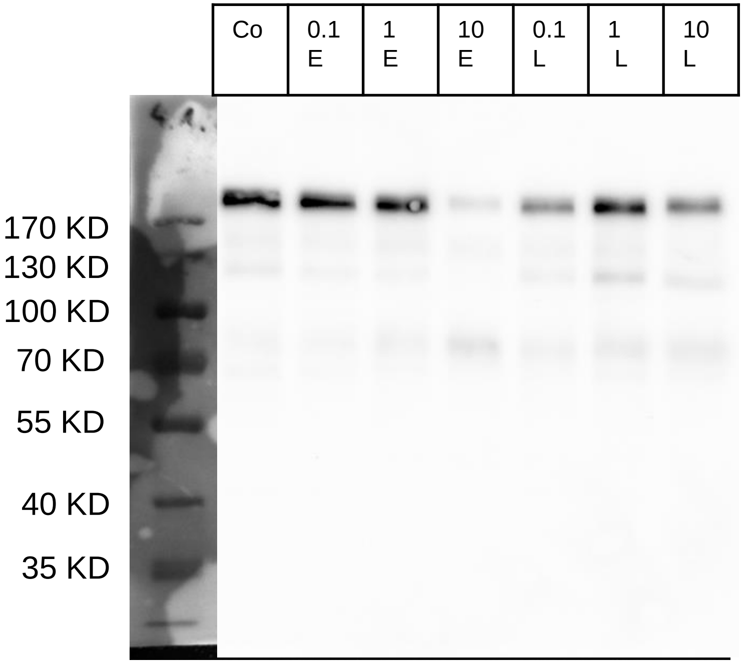

EGFR: Panc02 cells: 06.01.2021

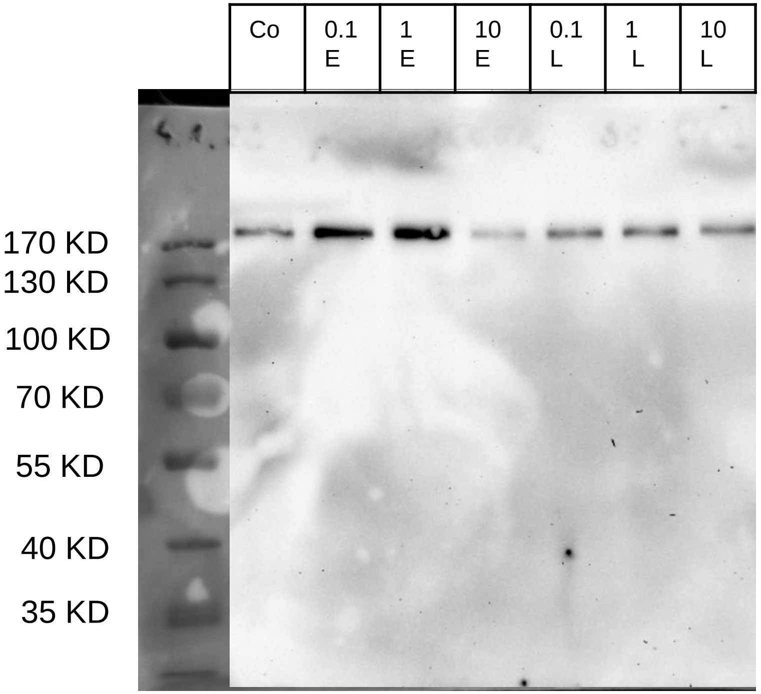

β-actin (Panc02 cells 07.01.2021)

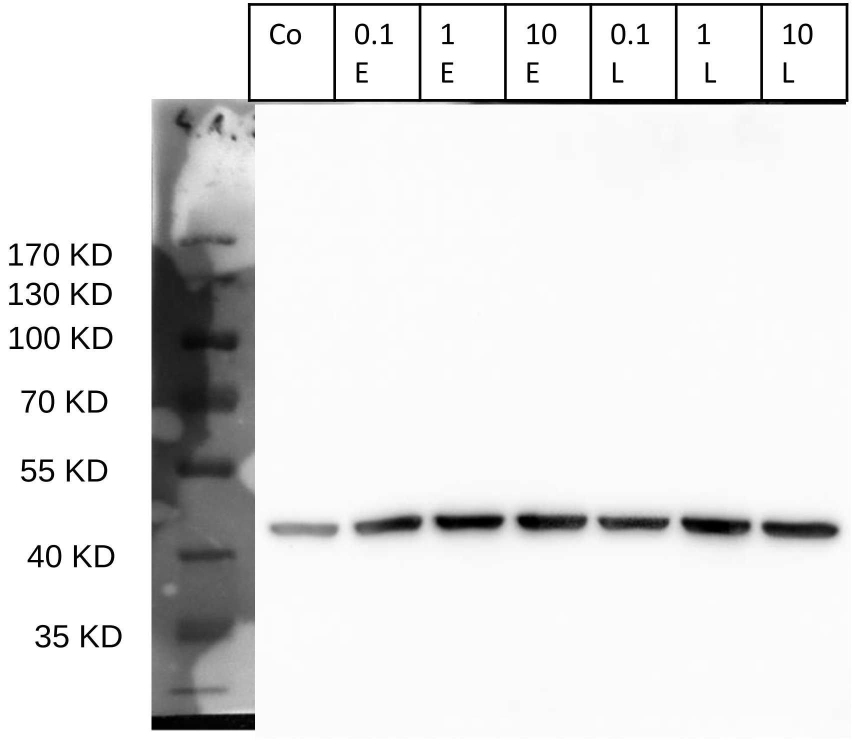

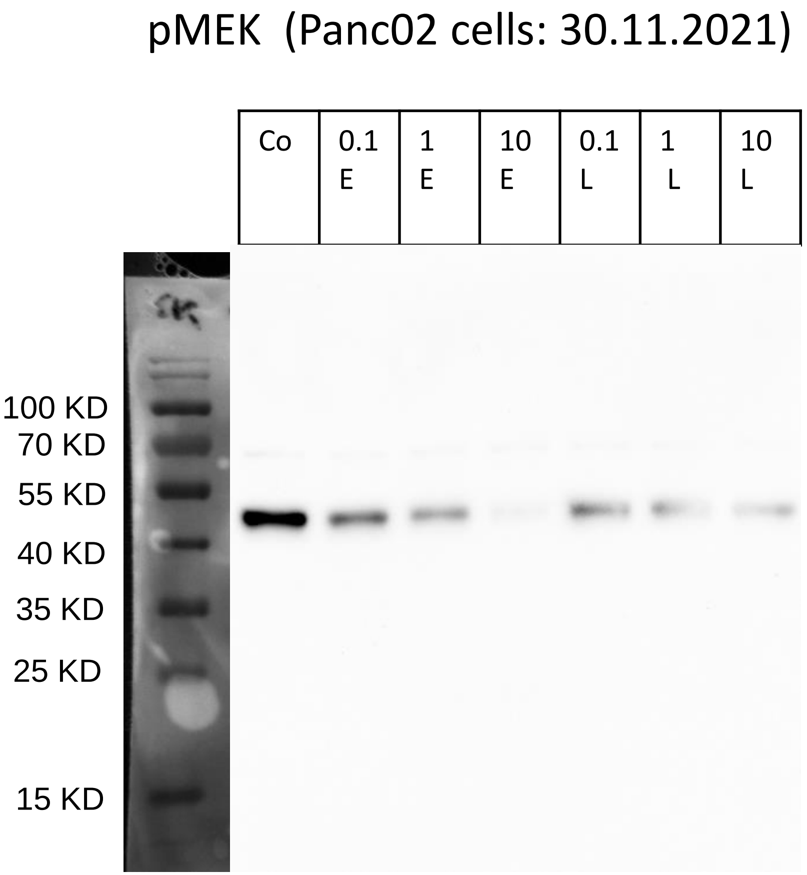
pMEK (Panc02 cells: 30.11.2021)
Co
0.1 E
1 E
10 E
0.1 L
1 L
10 L
100 KD
70 KD
55 KD
40 KD
35 KD
25 KD
15 KD

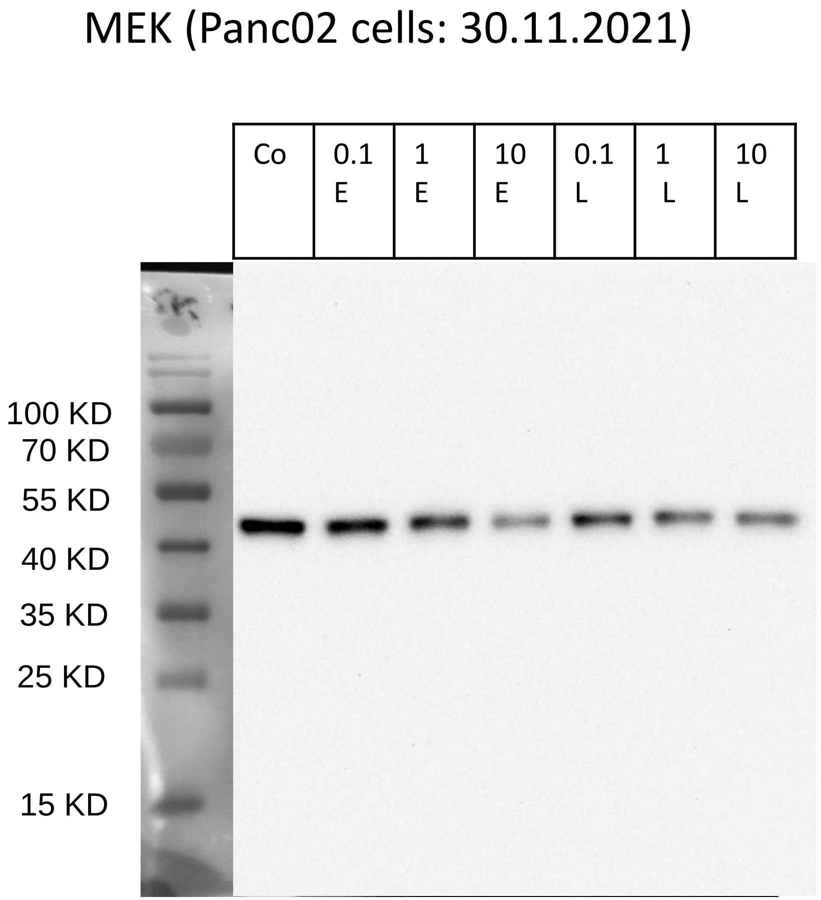
MEK (Panc02 cells: 30.11.2021)
Co
0.1 E
1 E
10 E
0.1 L
1 L
10 L
100 KD
70 KD
55 KD
40 KD
35 KD
25 KD
15 KD

β-actin: (Panc02 cells: 02.12.2021)

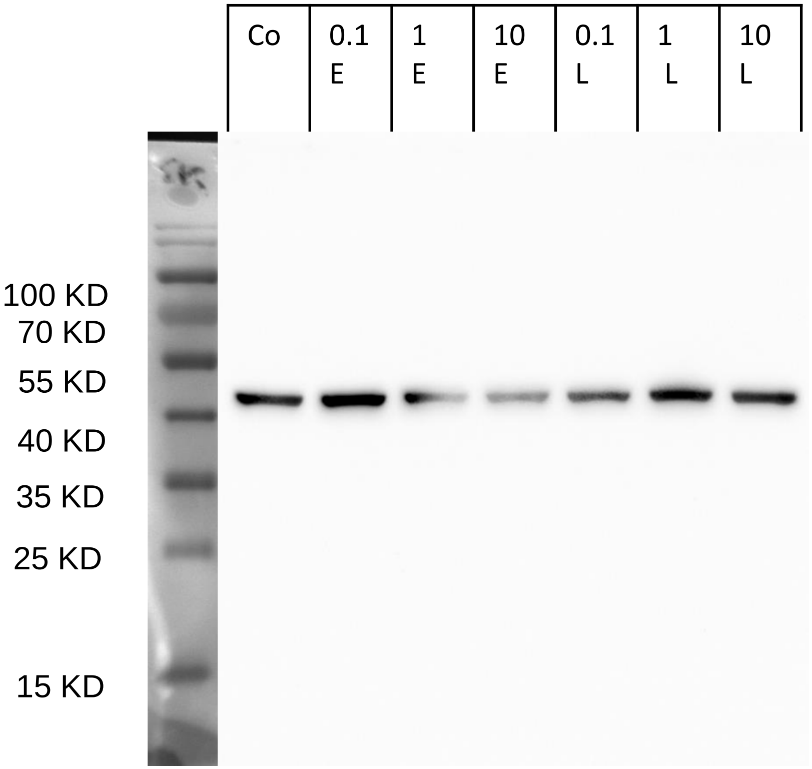

pERK Panc02 cells 30.11.2021

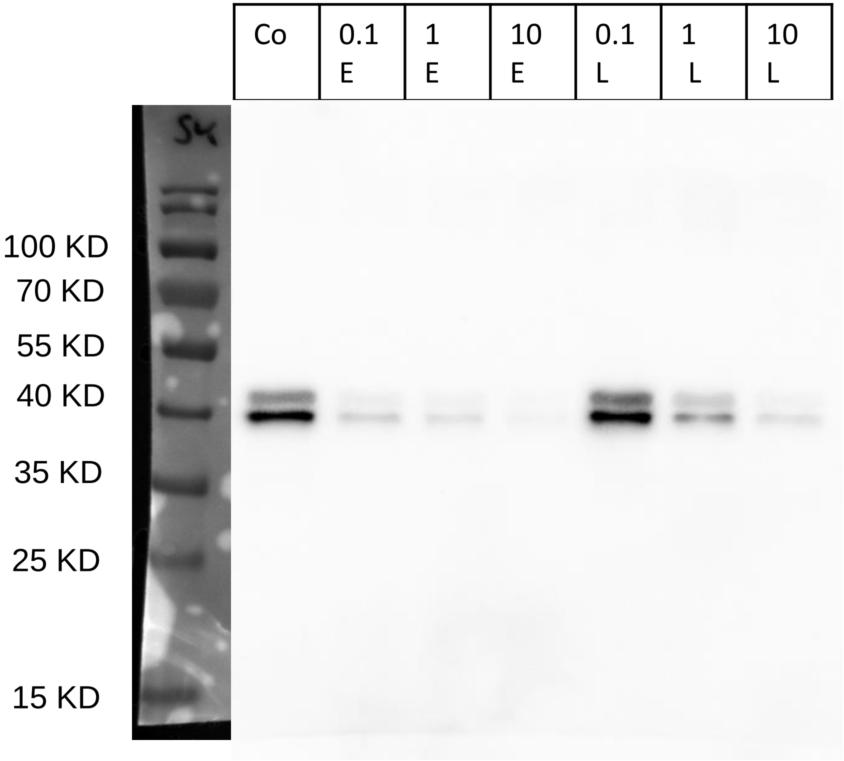

ERK Panc02 cells: 01.12.2021

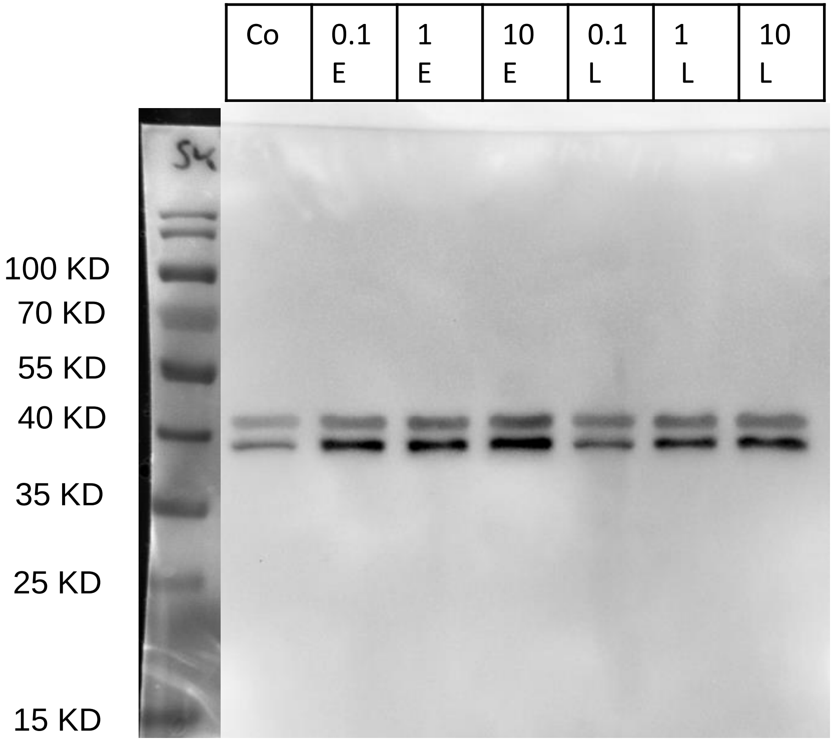

β-actin (Panc02 cells 02.12.2021)

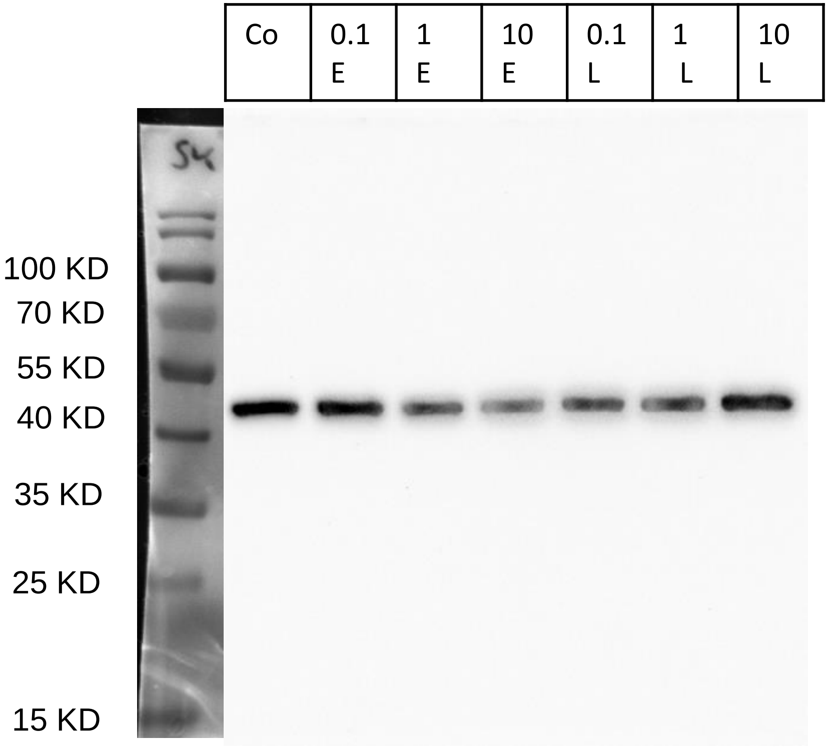

pEGFR: Panc02 cells: 14.03.2022

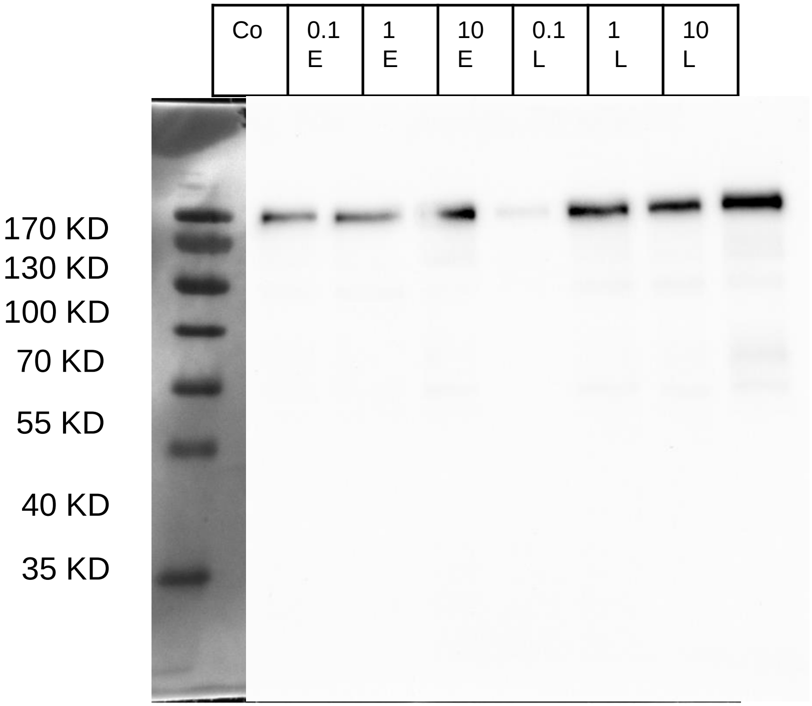

EGFR: Panc02 cells: 16.03.2022

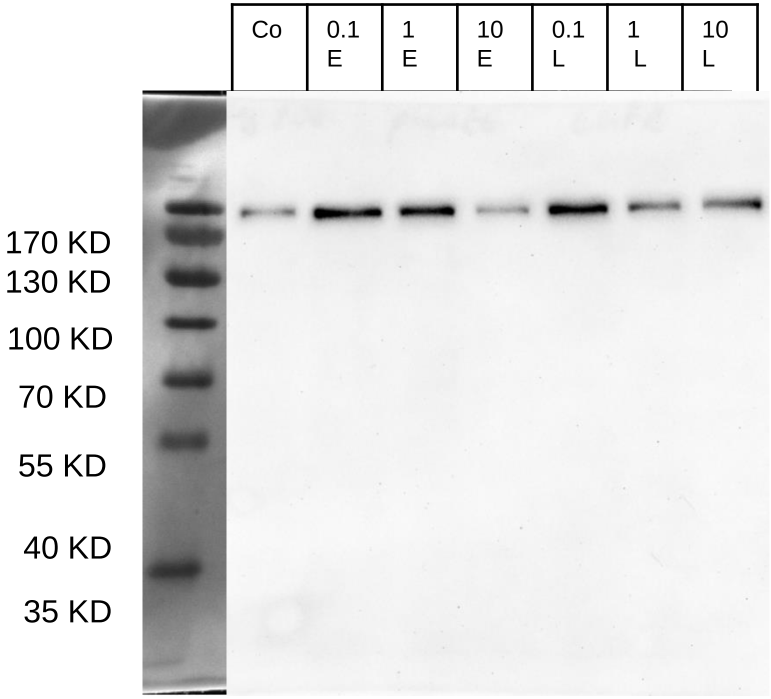

β-actin (Panc02 cells 17.03.2022)

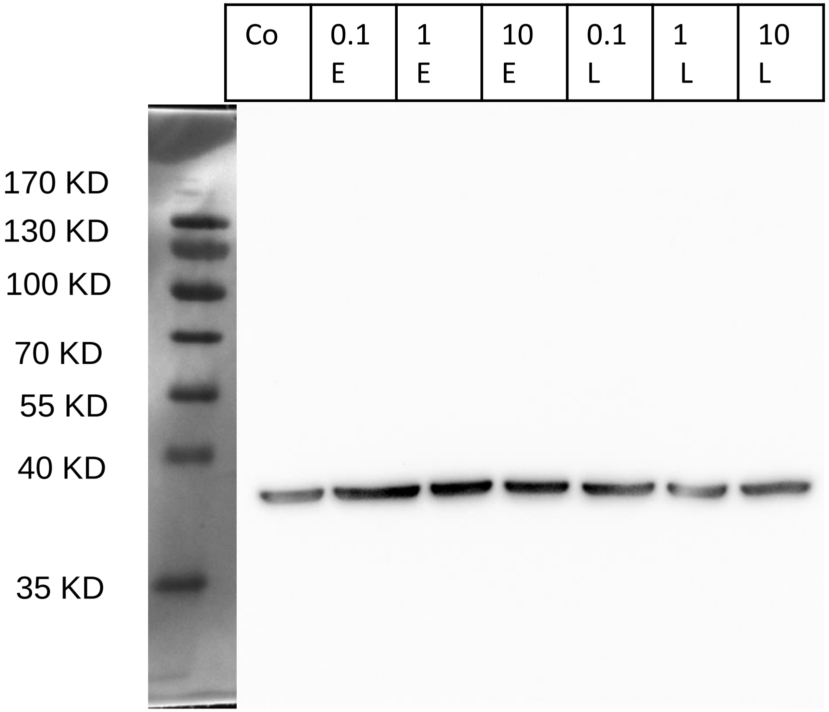

pMEK (Panc02 cells: 14.03.2022)

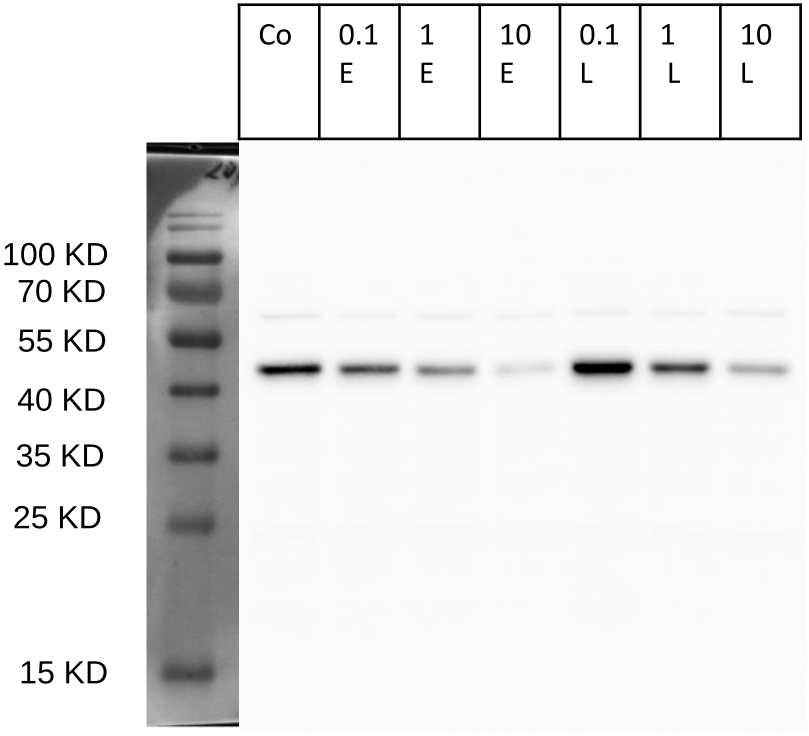

MEK (Panc02 cells: 16.03.2022)

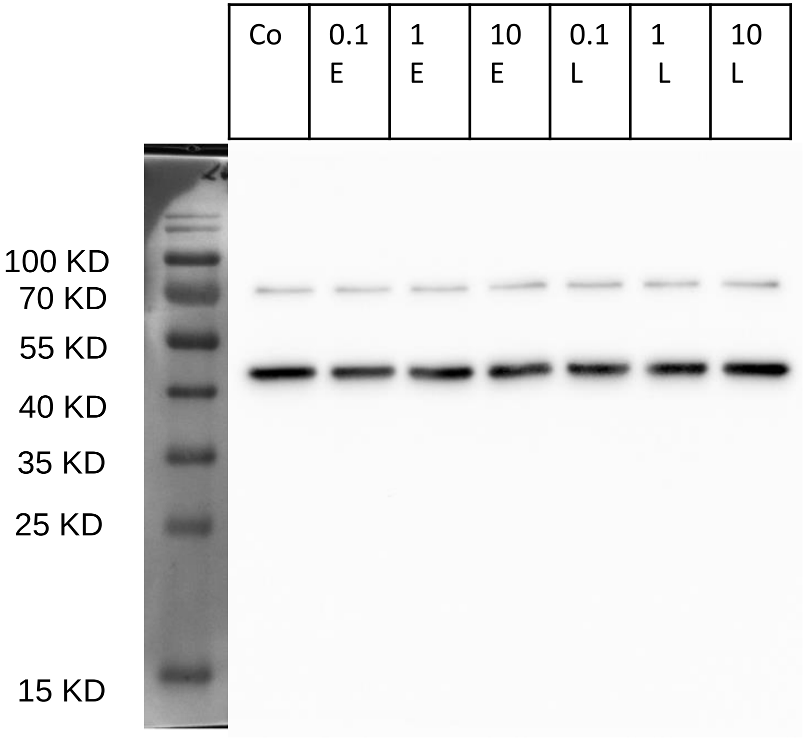

β-actin: (Panc02 cells: 17.03.2022)

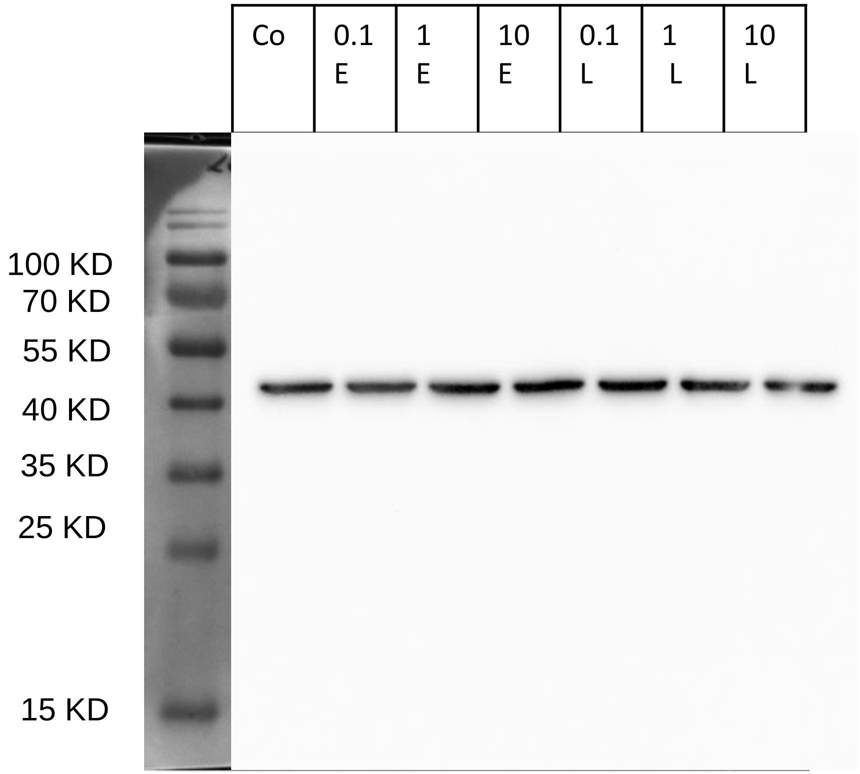

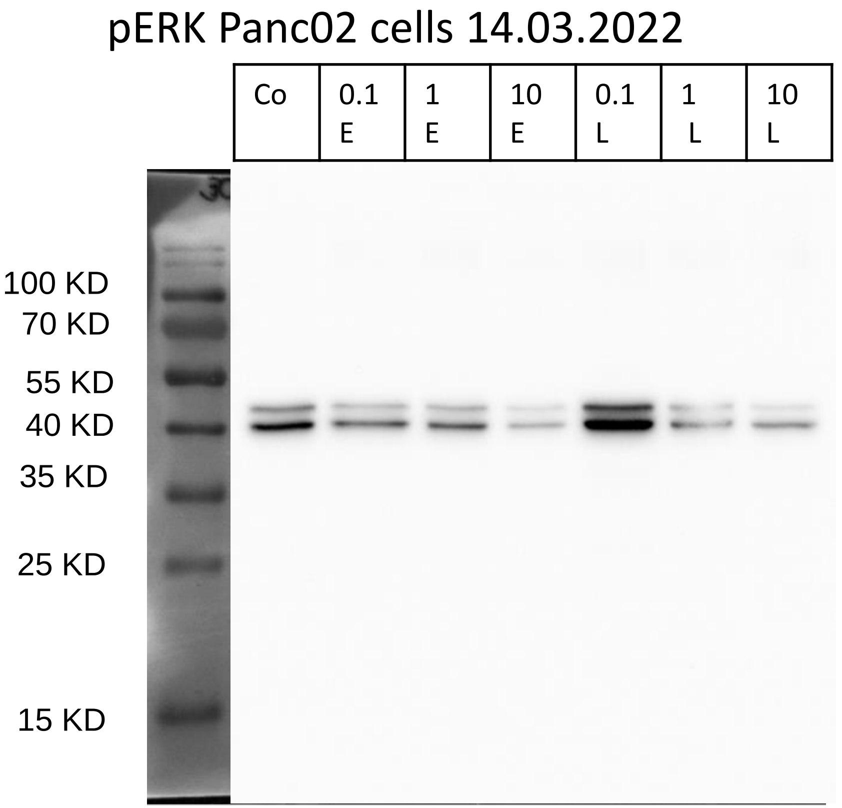

pERK Panc02 cells 14.03.2022
Co
0.1 E
1 E
10 E
0.1 L
1 L
10 L
100 KD
70 KD
55 KD
40 KD
35 KD
25 KD
15 KD

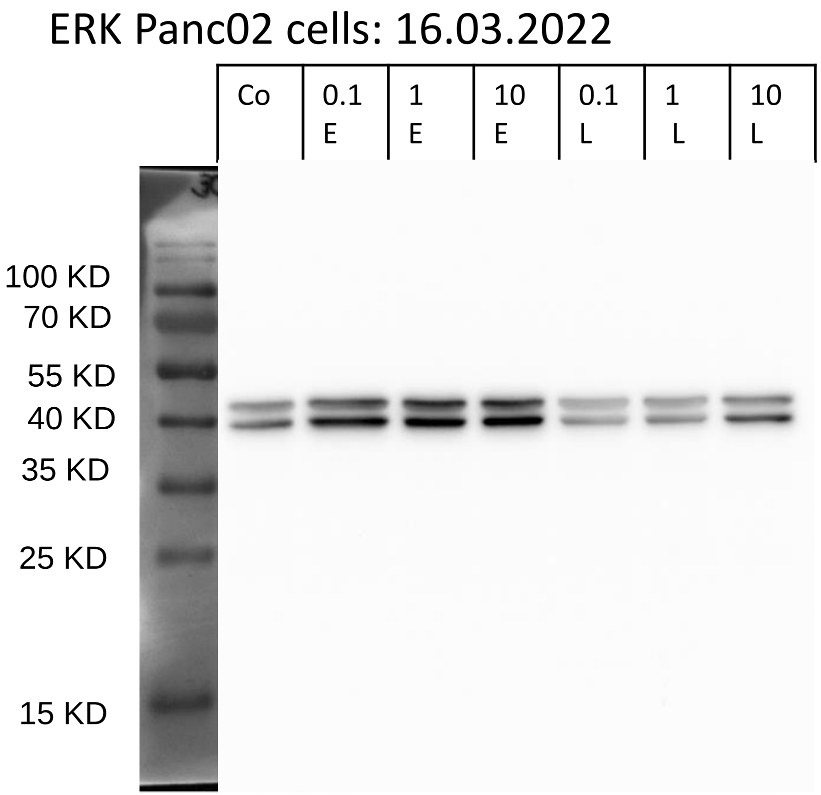

ERK Panc02 cells: 16.03.2022
Co
0.1 E
1 E
10 E
0.1 L
1 L
10 L
100 KD
70 KD
55 KD
40 KD
35 KD
25 KD
15 KD

β-actin (Panc02 cells 17.03.2022)

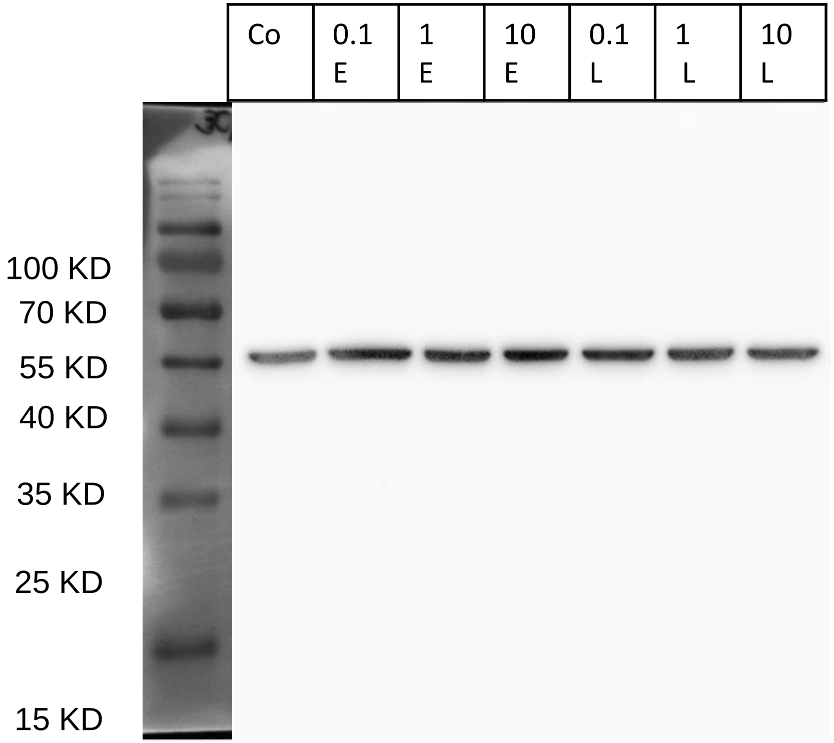

pEGFR: Panc02 cells: 26.04.2022

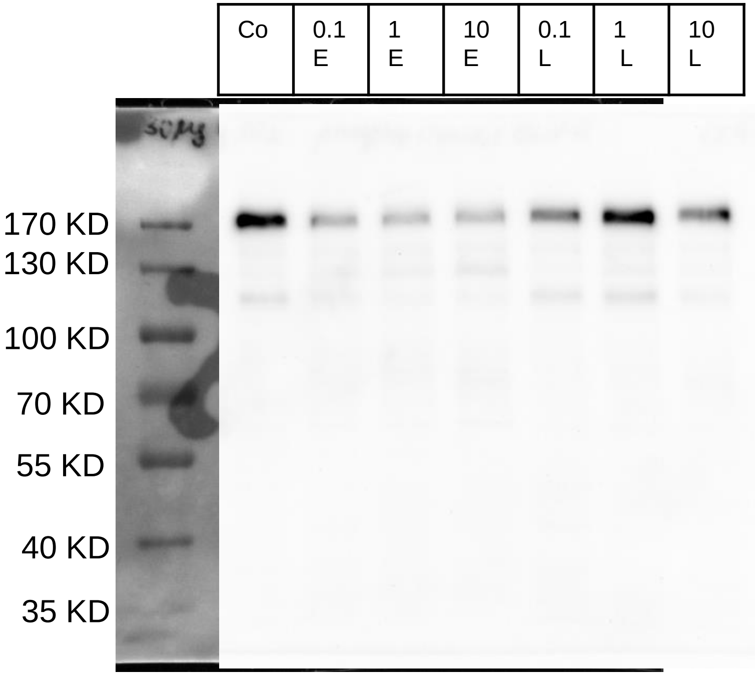

EGFR: Panc02 cells: 27.04.2022

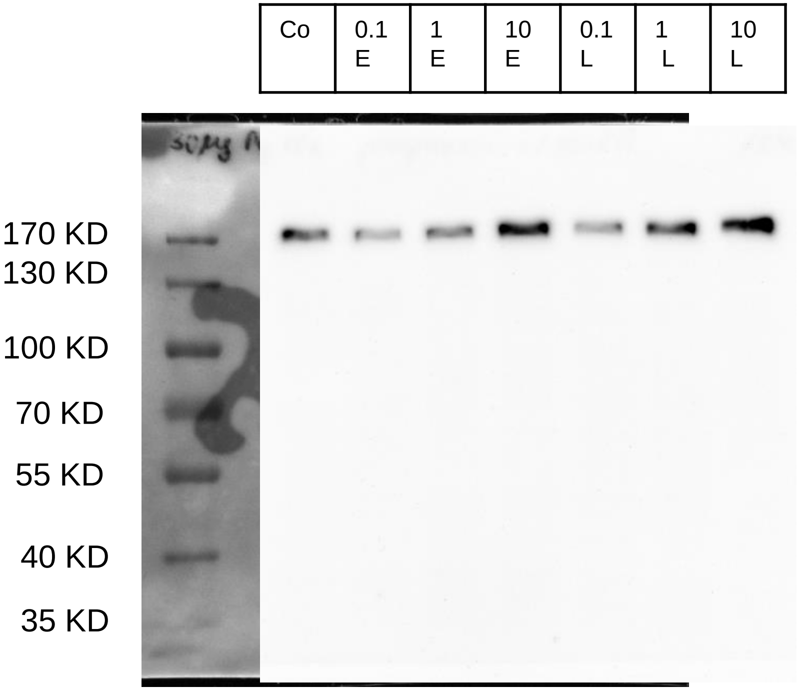

β-actin (Panc02 cells 28.04.2022)

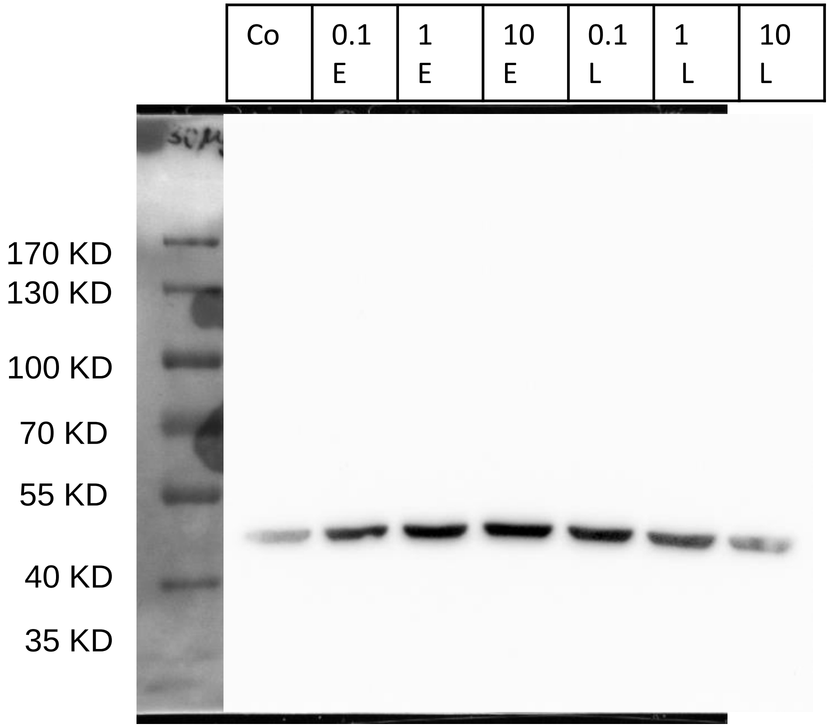

pMEK (Panc02 cells: 26.04.2022)

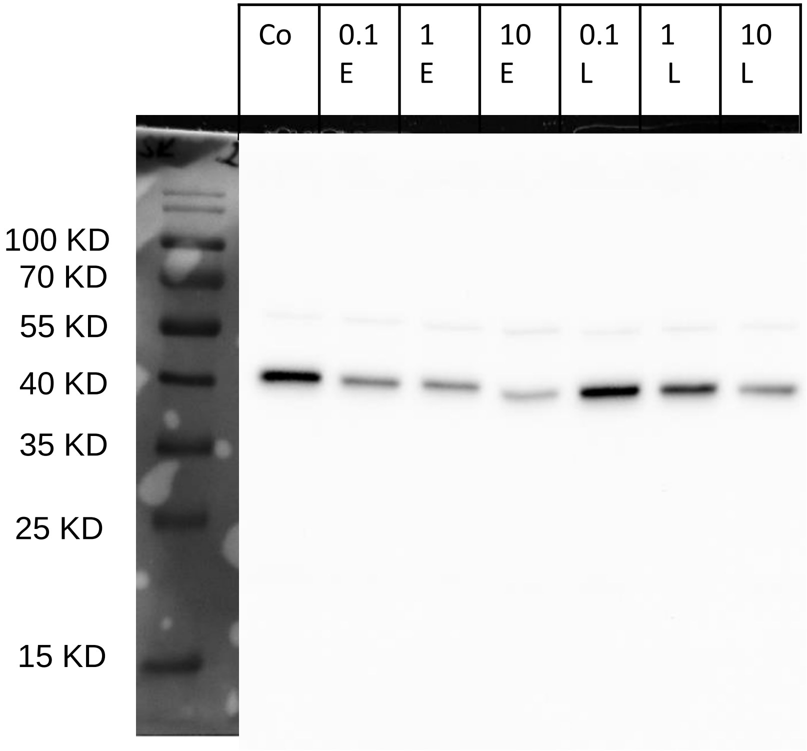

MEK (Panc02 cells: 27.04.2022)

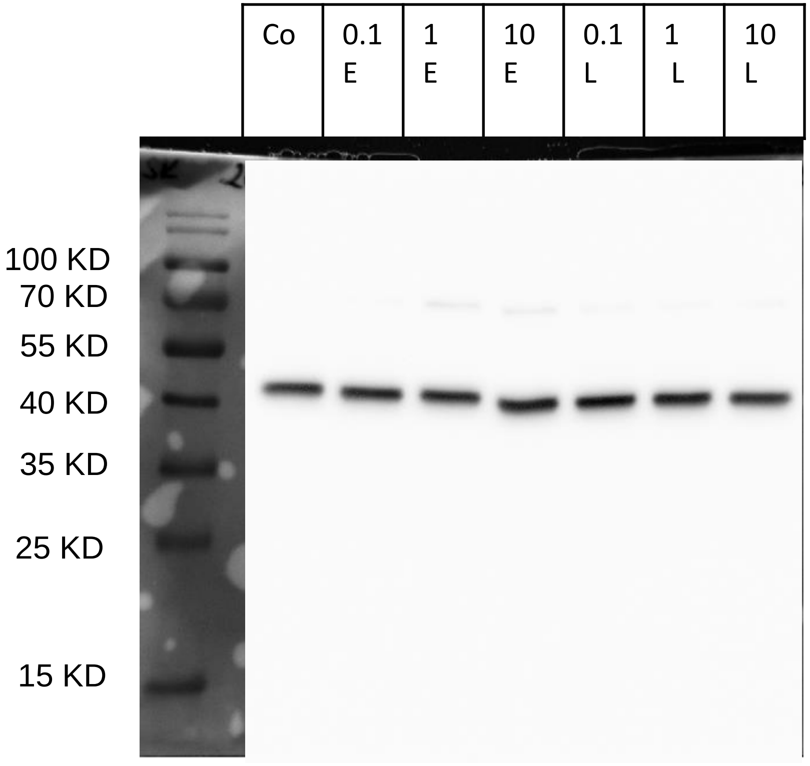

β-actin: (Panc02 cells: 28.04.2022)

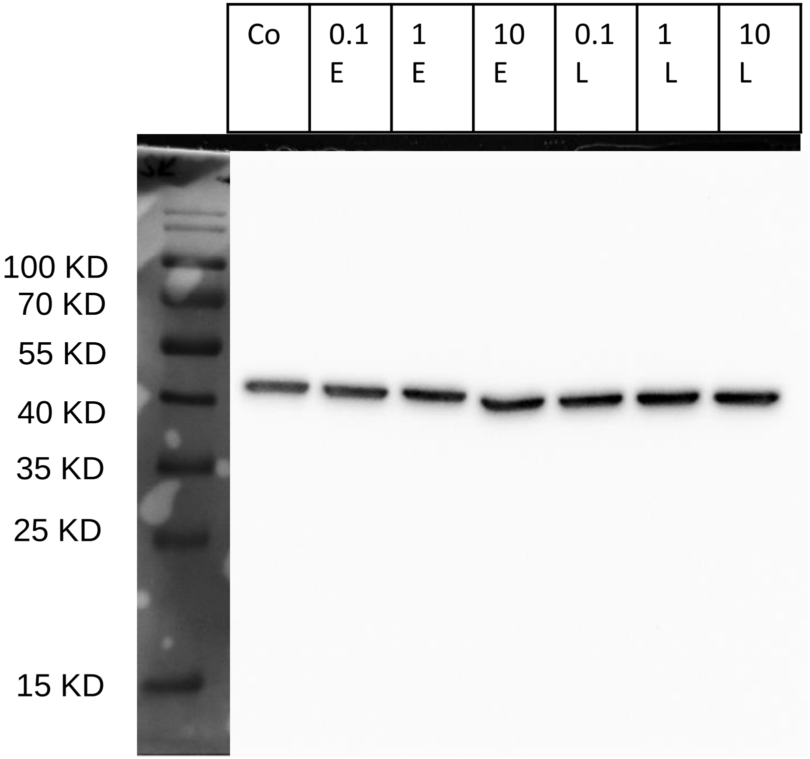

pERK Panc02 cells 26.04.2022

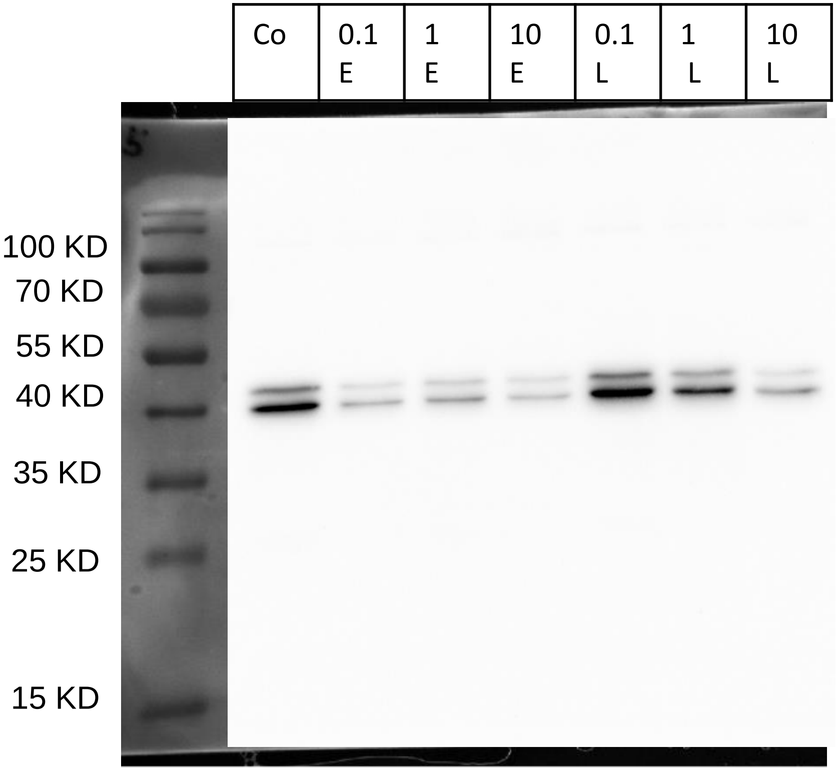

ERK Panc02 cells: 27.04.2022

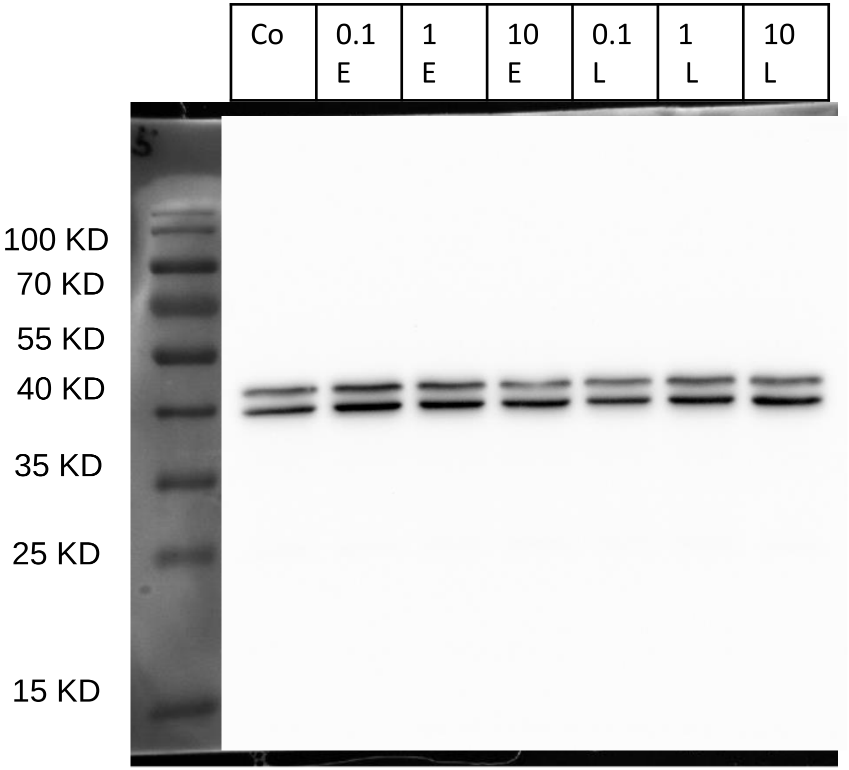

β-actin (Panc02 cells 28.04.2022)

Co	0.1<br>E	1<br>E	10<br>E	0.1<br>L	1<br>L	10<br>L

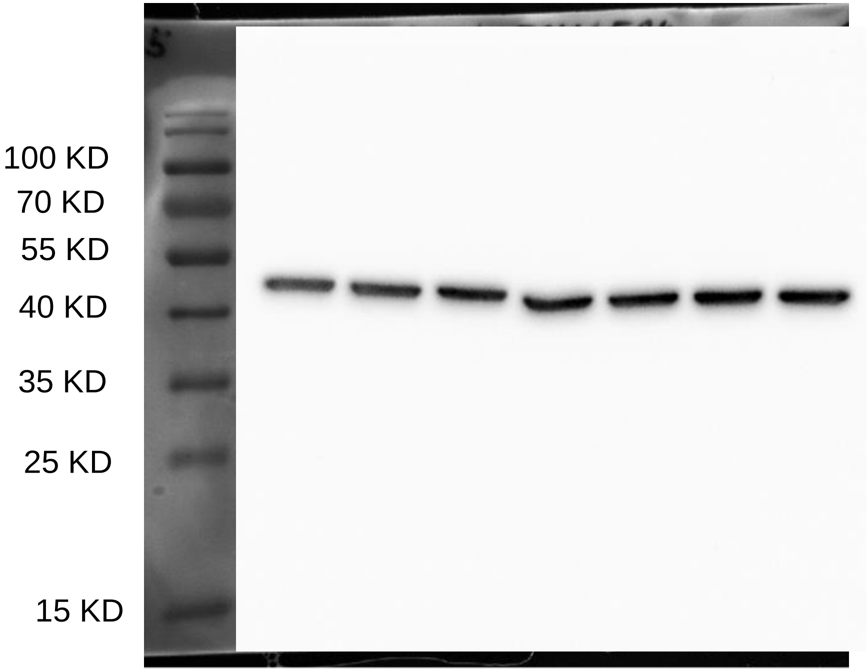

pEGFR (CT-26 / Panc02 cells + 1 µM LXH-254, 1 µM Erlotinib, 17.06.2022)

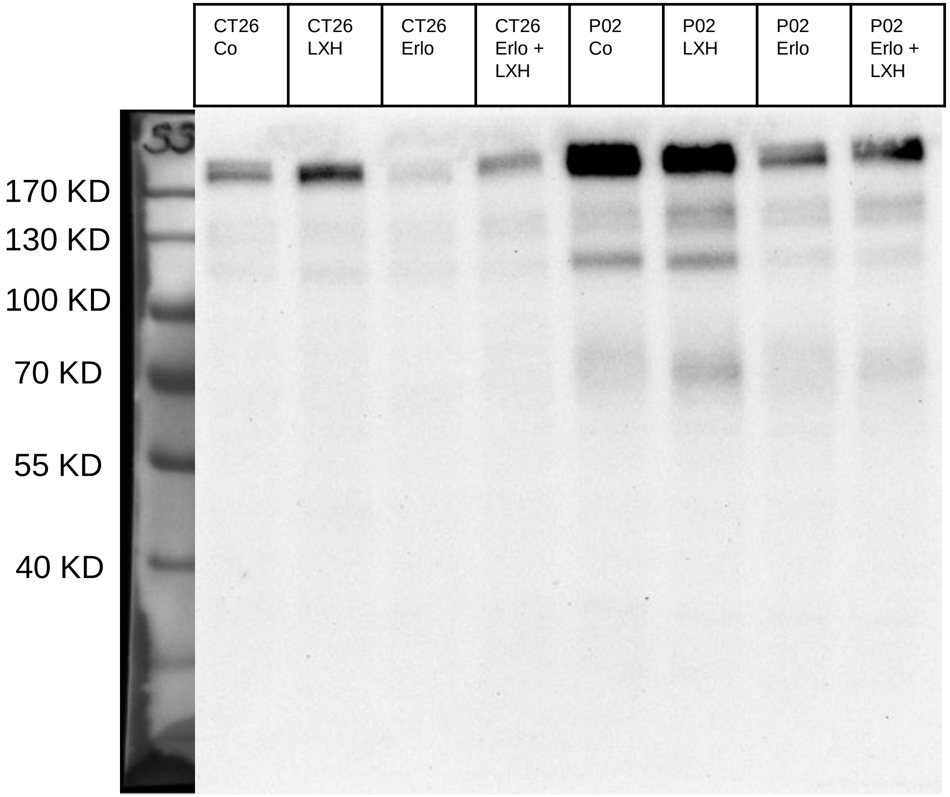

EGFR (CT-26/ Panc02 cells + 1 µM LXH-254, 1 µM Erlotinib, 21.06.2022)

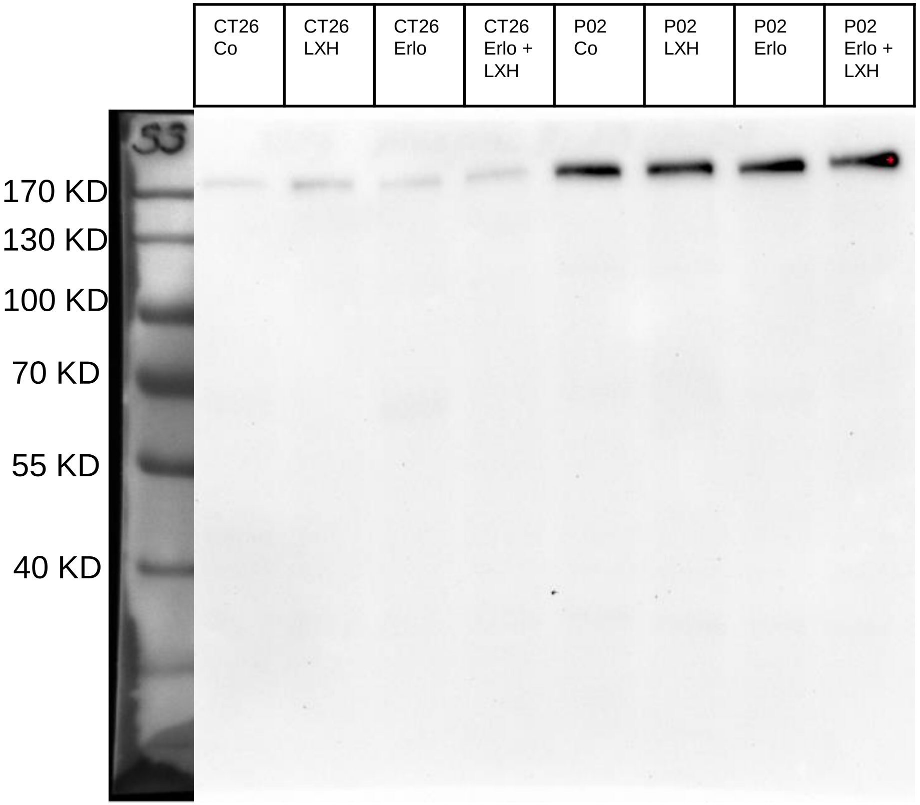

β-actin (pEGFR und EGFR Panc02 cells + 1 µM LXH-254, 1 µM Erlotinib, 22.06.22)

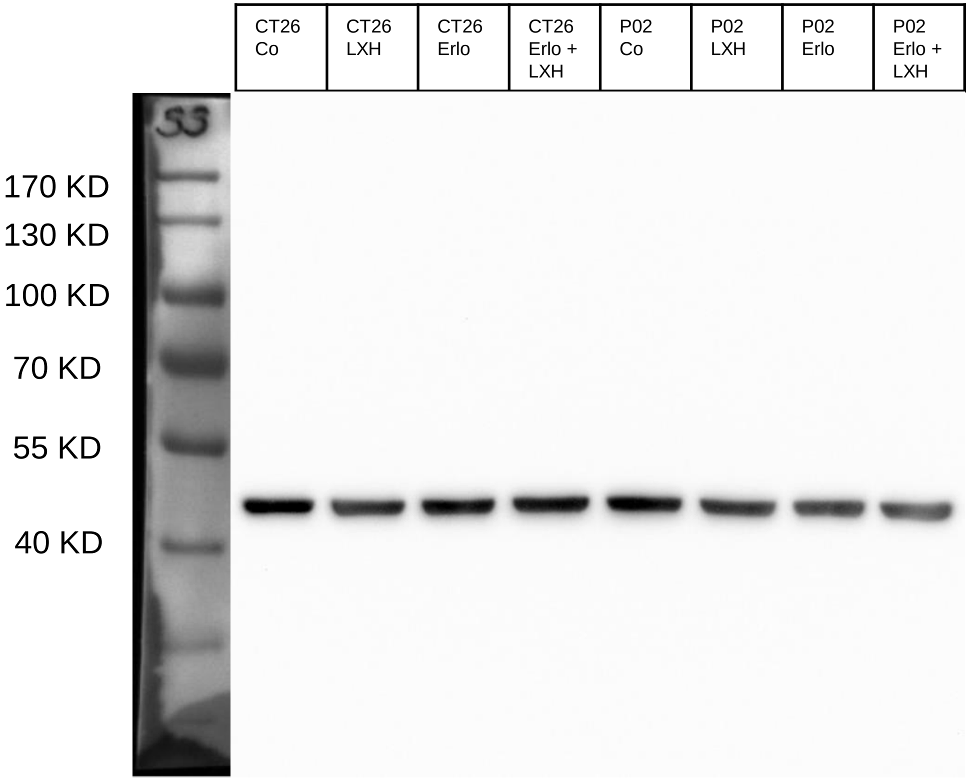

pMEK (Panc02 cells + 1 µM LXH- 254, 1 µM Erlotinib, 17.06.2022)

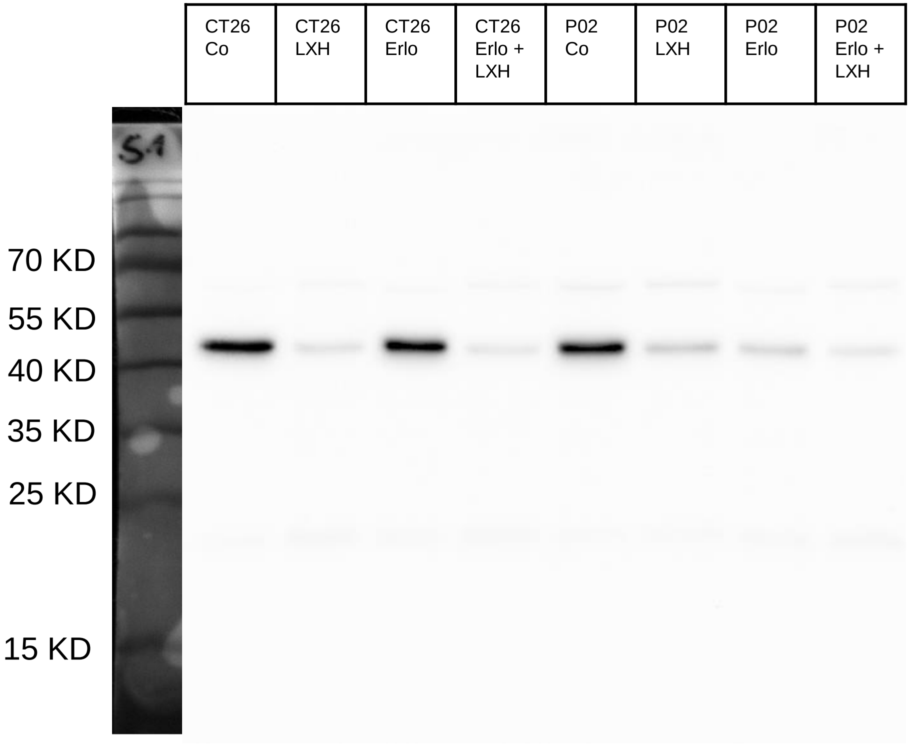

MEK (Panc02 cells + 1µM LXH-254, 1 µM Erlotinib, 21.06.2022)

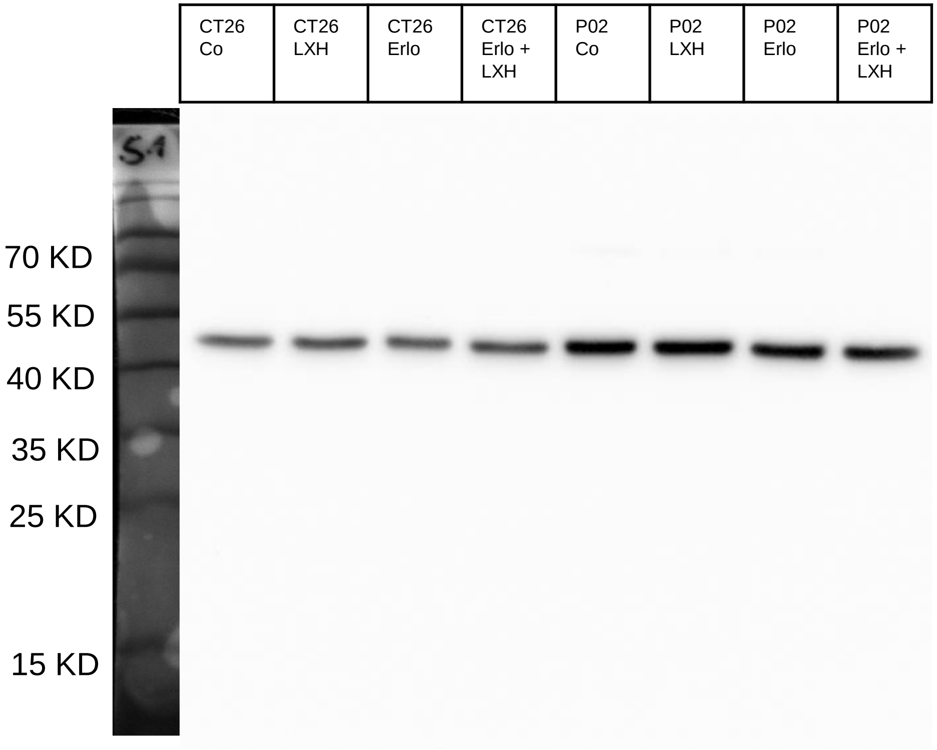

β-actin (pMek/Mek, CT-26/Panc02 cells + 1 µM LXH-254, 1 µM Erlotinib, 22.06.2022)

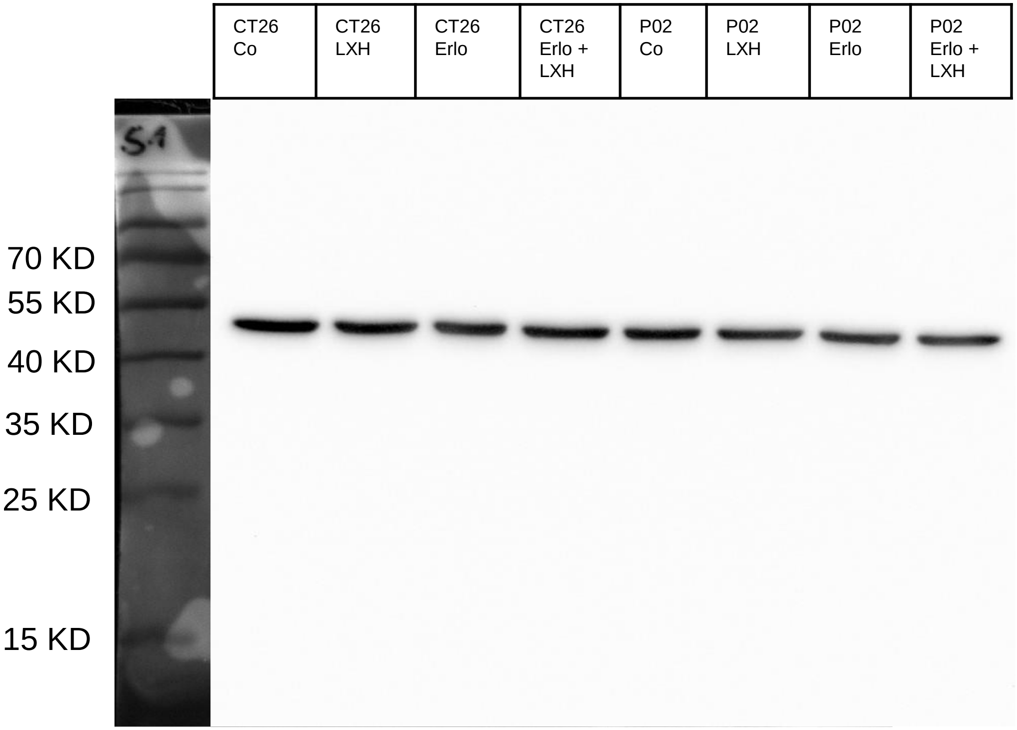

pERK (Panc02 cells + 1 µM LXH-254, 1 µM Erlotinib, 17.06.2022)

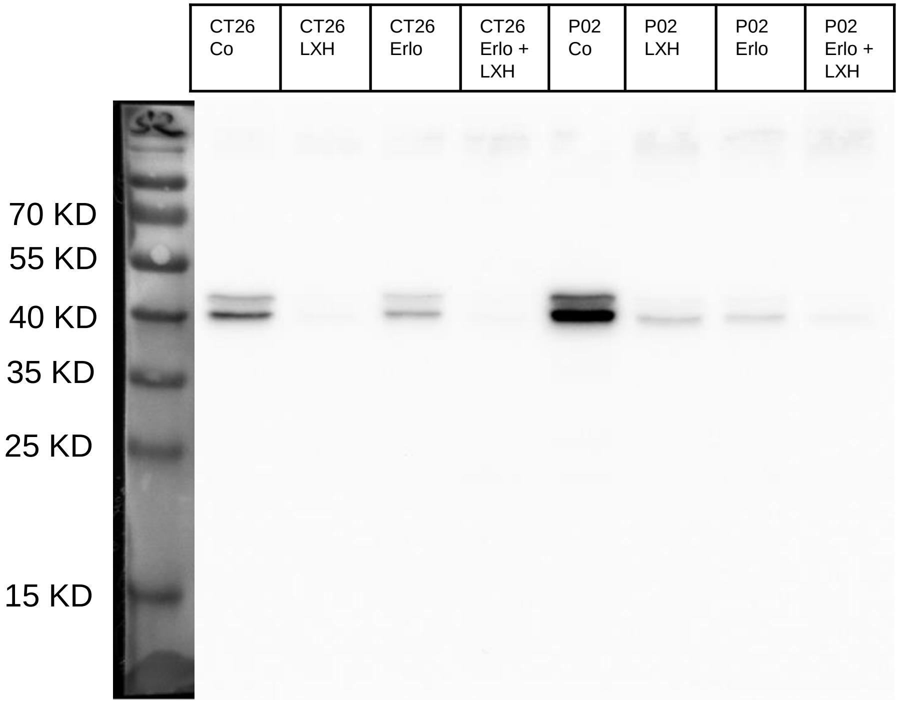

ERK (Panc02 cells + 1 µM LXH-254, 1 µM Erlotinib, 21.06.2022)

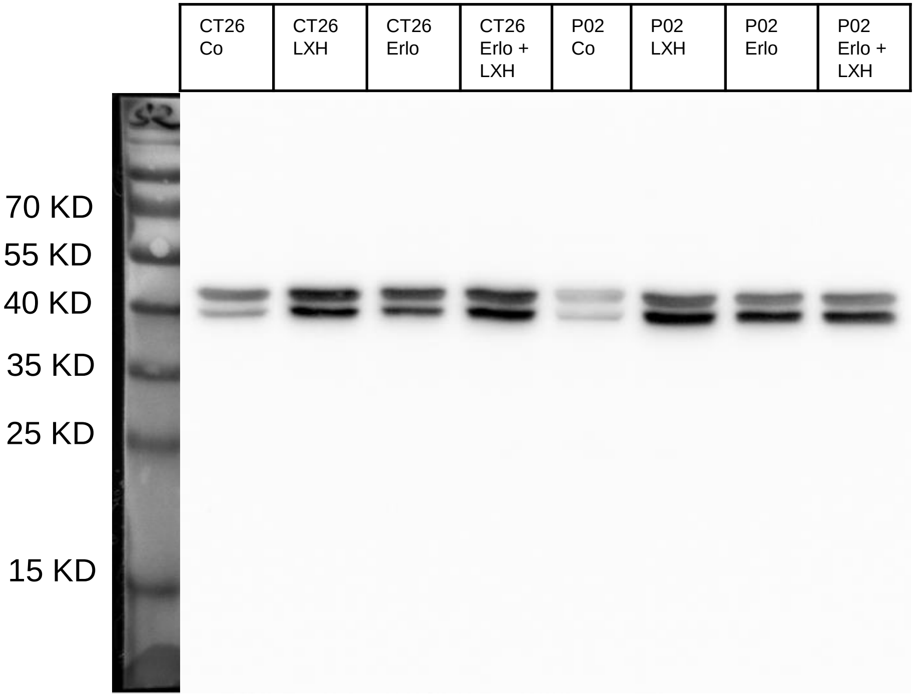

β-actin (pErk/Erk, Panc02-Zellen + 1 µM LXH-254, 1 µM Erlotinib, 22.06.2022)

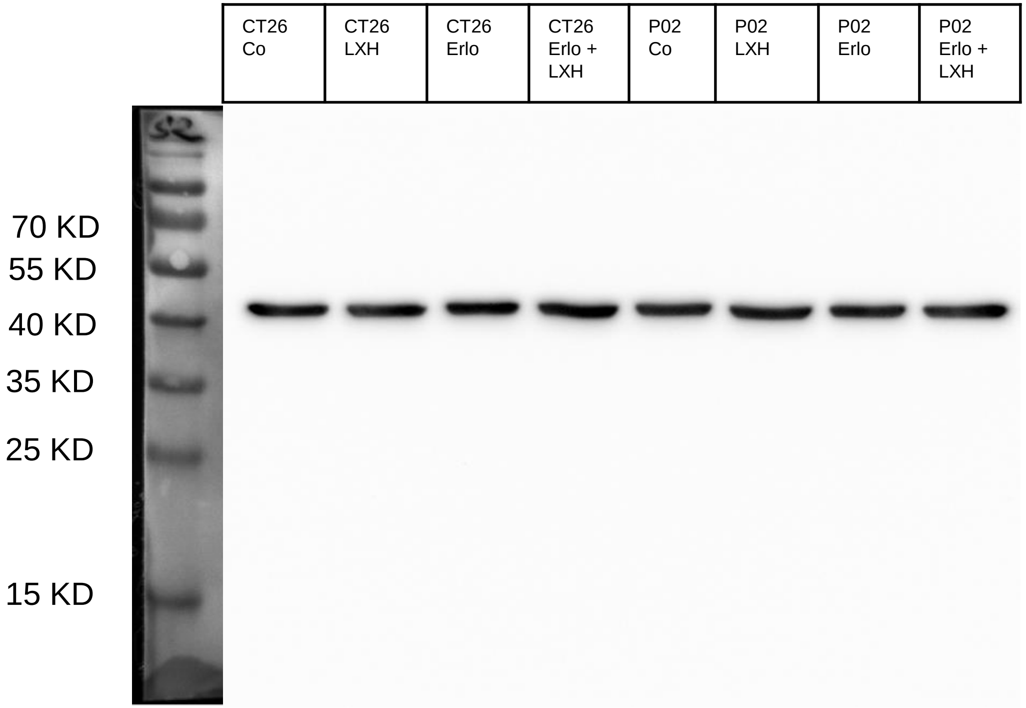

pEGFR (CT-26 / Panc02 cells + 1 µM LXH-254, 1 µM Erlotinib, 06.07.2022)

EGFR (CT-26/ Panc02 cells + 1 µM LXH-254, 1 µM Erlotinib, 07.07.2022)

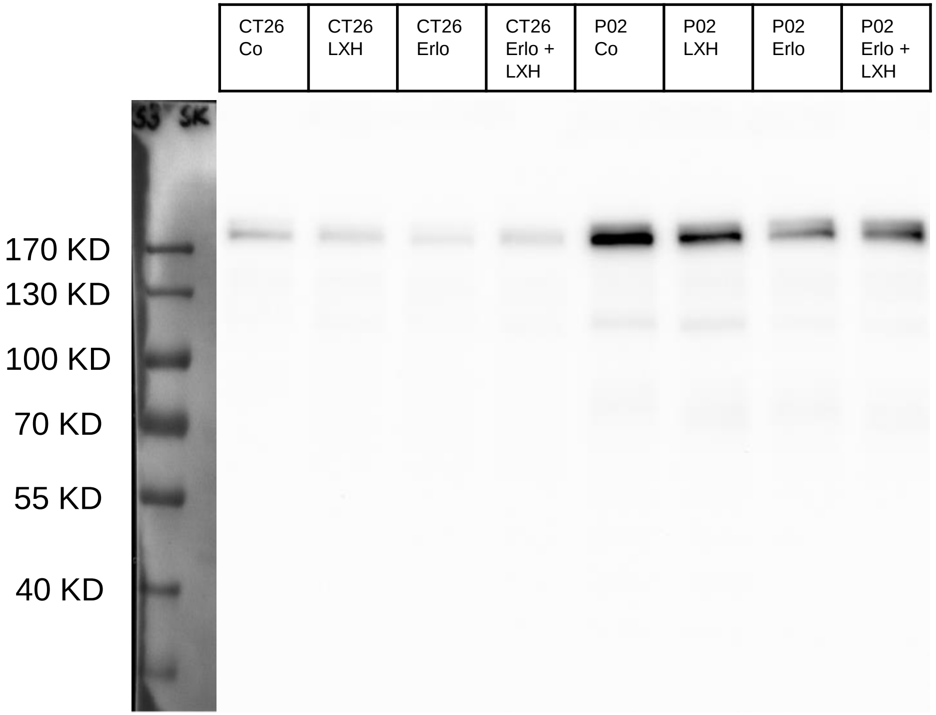

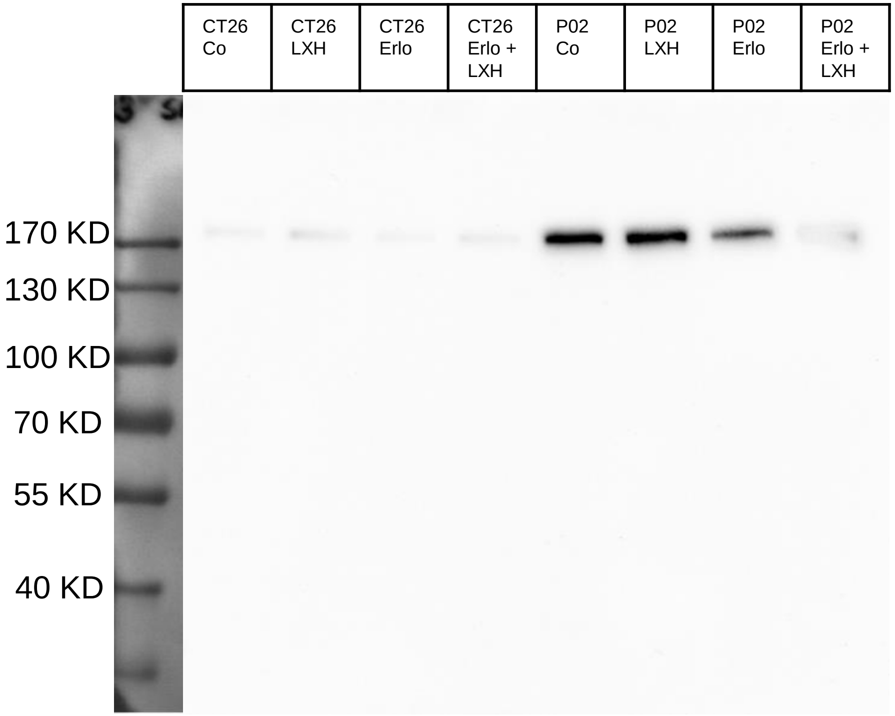

β-actin (pEGFR/EGFR, Panc02-Zellen + 1 µM LXH-254, 1 µM Erlotinib, 08.07.2022)

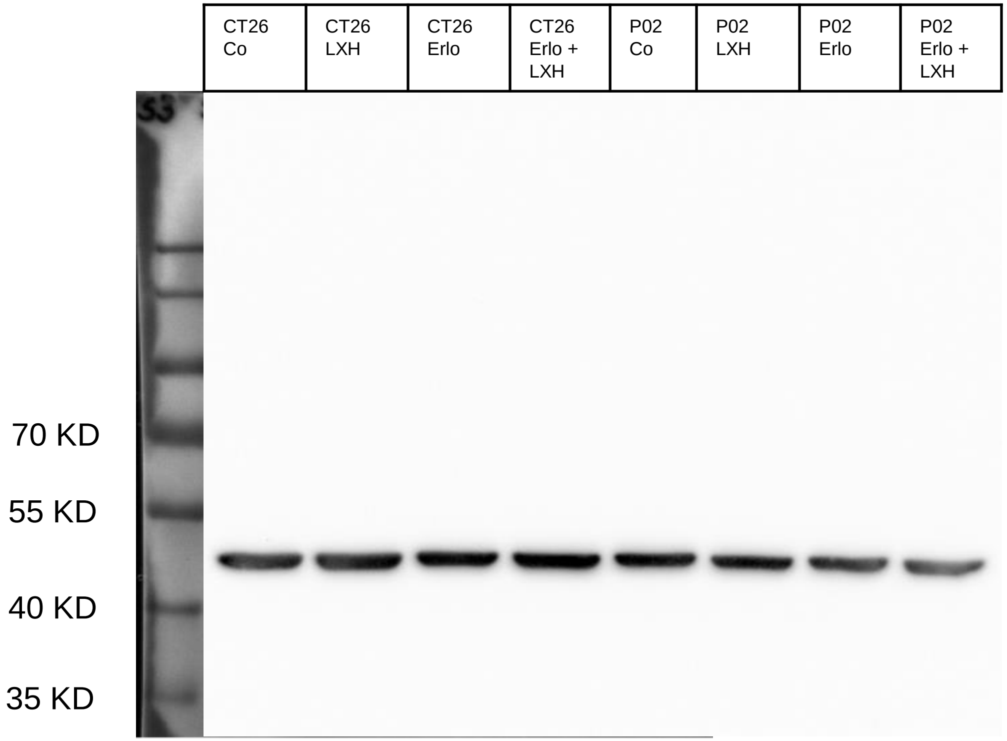

pMEK (CT-26 / Panc02 cells + 1 µM LXH-254, 1 µM Erlotinib, 06.07.2022)

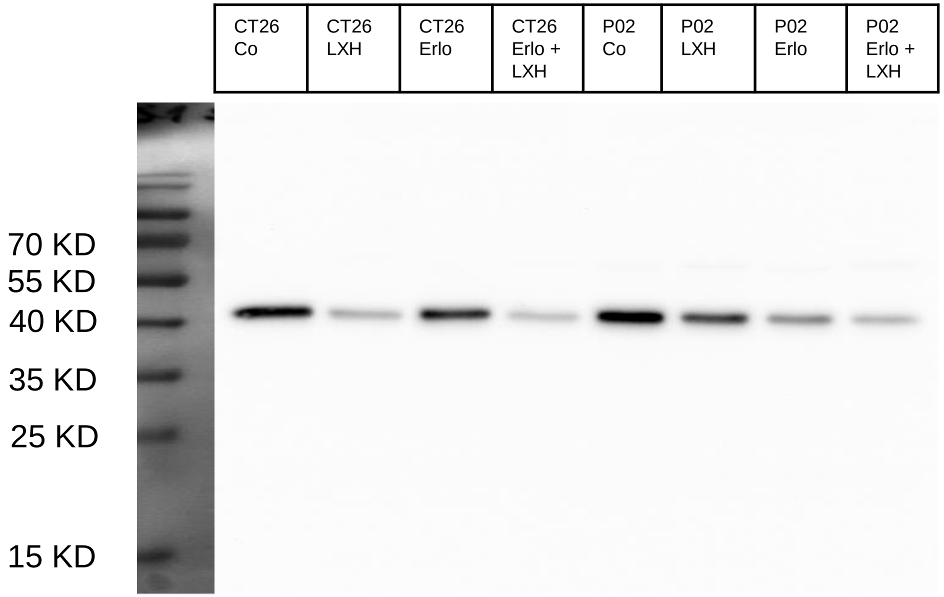

MEK (CT-26/ Panc02 cells + 1 µM LXH-254, 1 µM Erlotinib, 07.07.2022)

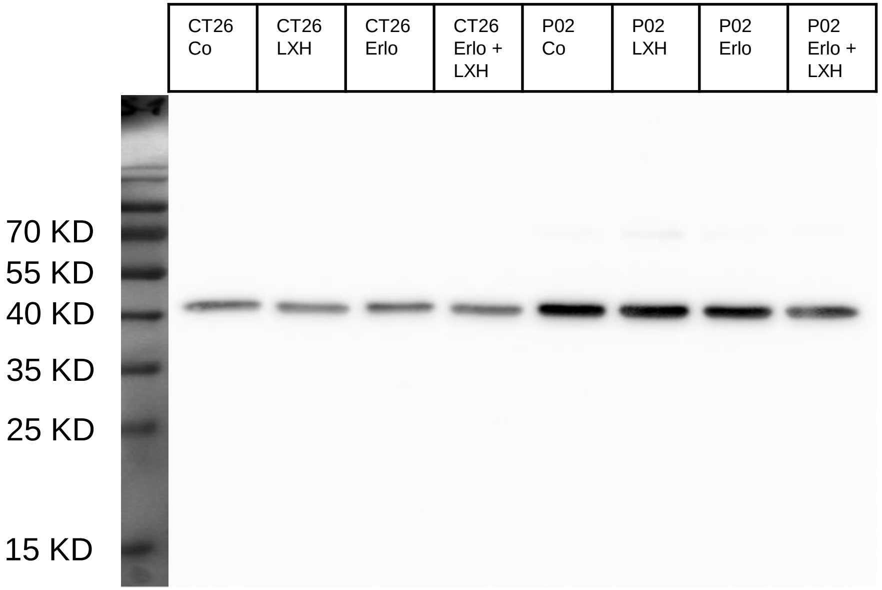

β-actin (pMek/Mek, Panc02-Zellen + 1 µM LXH-254, 1 µM Erlotinib, 08.07.2022)

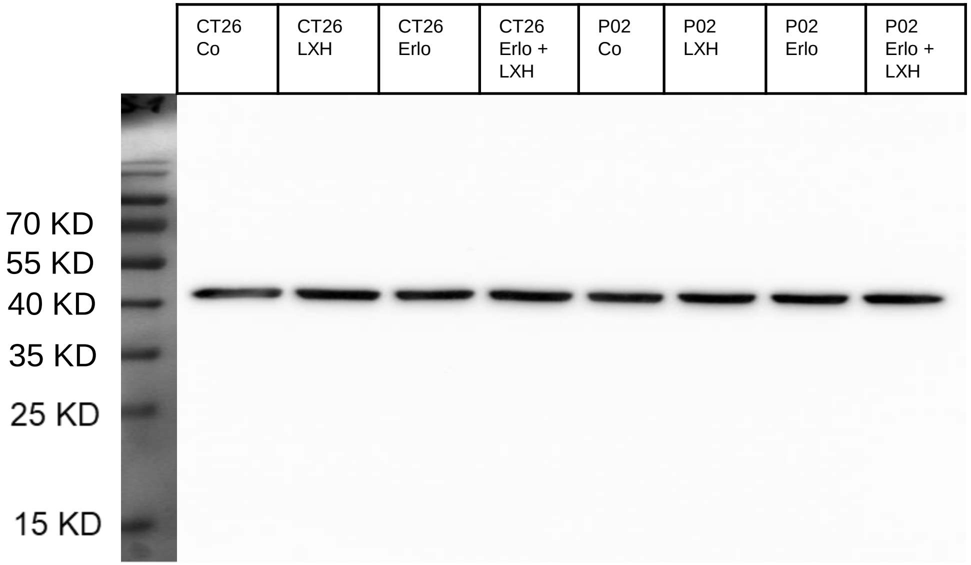

pERK (CT-26 / Panc02 cells + 1 µM LXH-254, 1 µM Erlotinib, 06.07.2022)

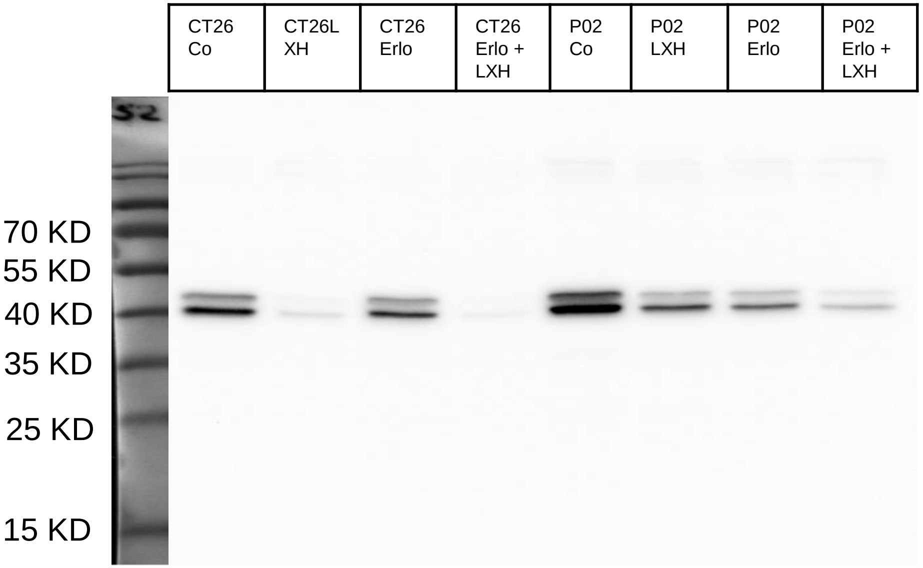

ERK (CT-26/ Panc02 cells + 1 µM LXH-254, 1 µM Erlotinib, 07.07.2022)

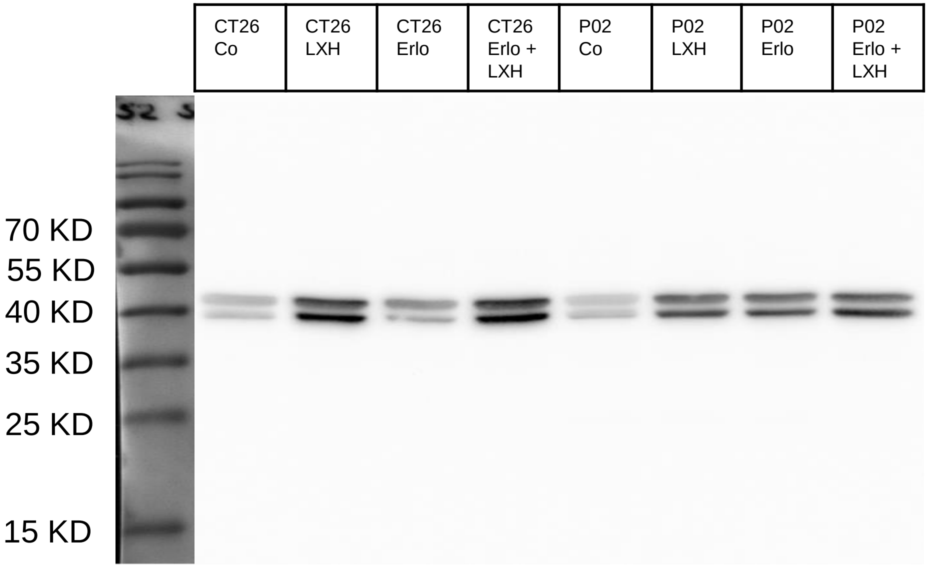

β-actin (pERK/ERK, Panc02-Zellen + 1 µM LXH-254, 1 µM Erlotinib, 08.07.2022)

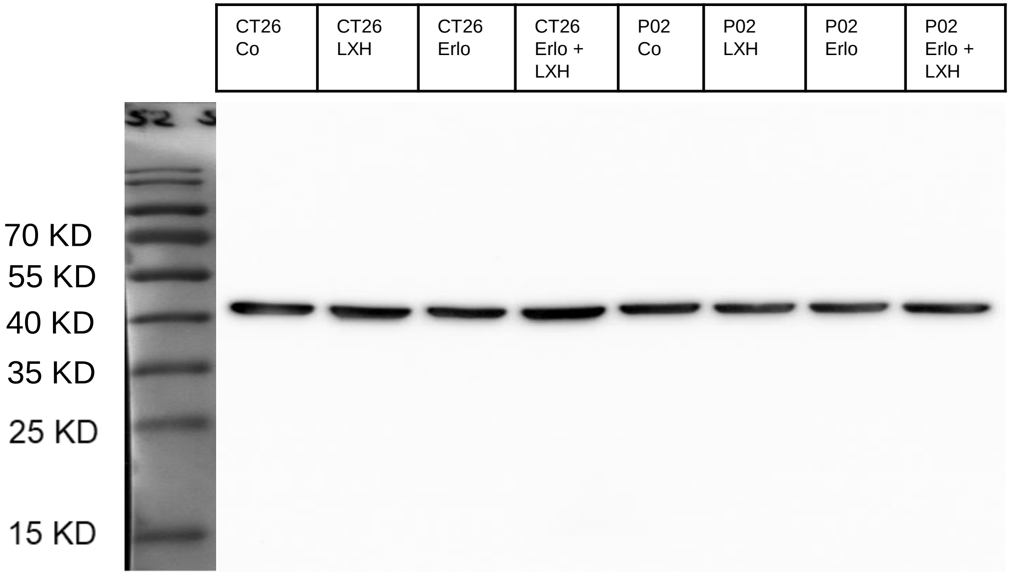

pEGFR (CT-26 / Panc02 cells + 1 µM LXH-254, 1 µM Erlotinib, 28.07.2022)

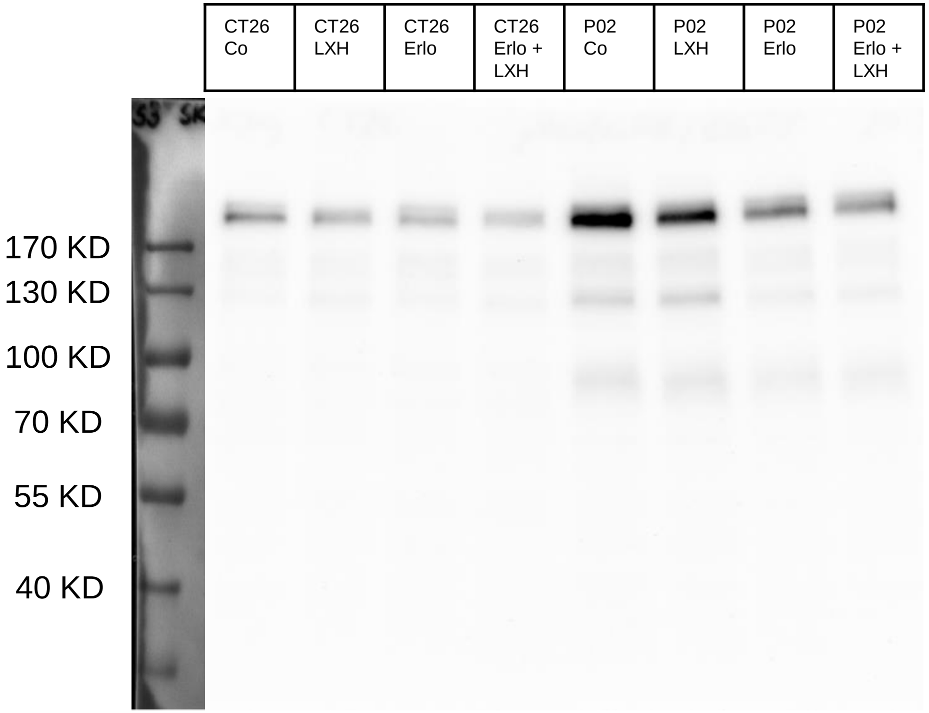

EGFR (CT-26/ Panc02 cells + 1 µM LXH-254, 1 µM Erlotinib, 29.07.2022)

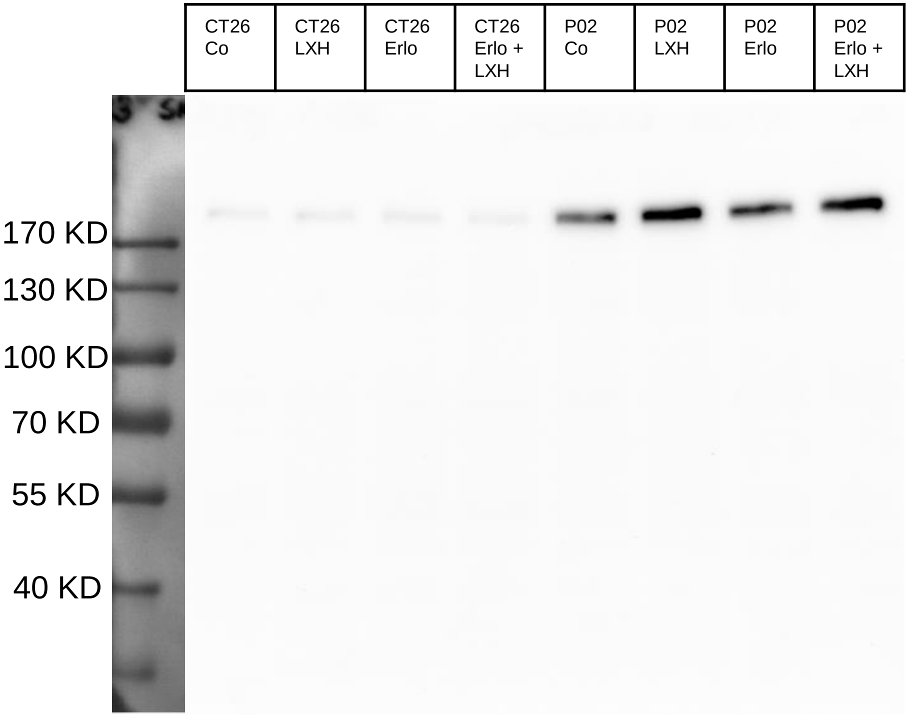

β-actin (pEGFR/EGFR, Panc02-Zellen + 1 µM LXH-254, 1 µM Erlotinib, 01.08.2022)

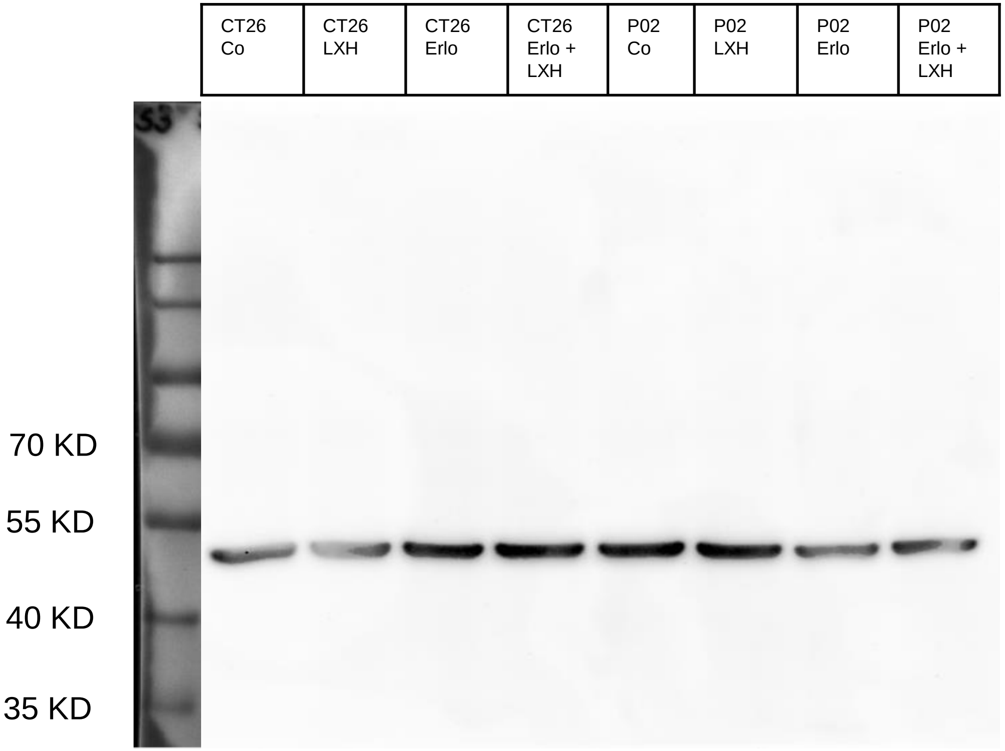

pMek (CT-26 / Panc02 cells + 1 µM LXH-254, 1 µM Erlotinib, 28.07.2022)

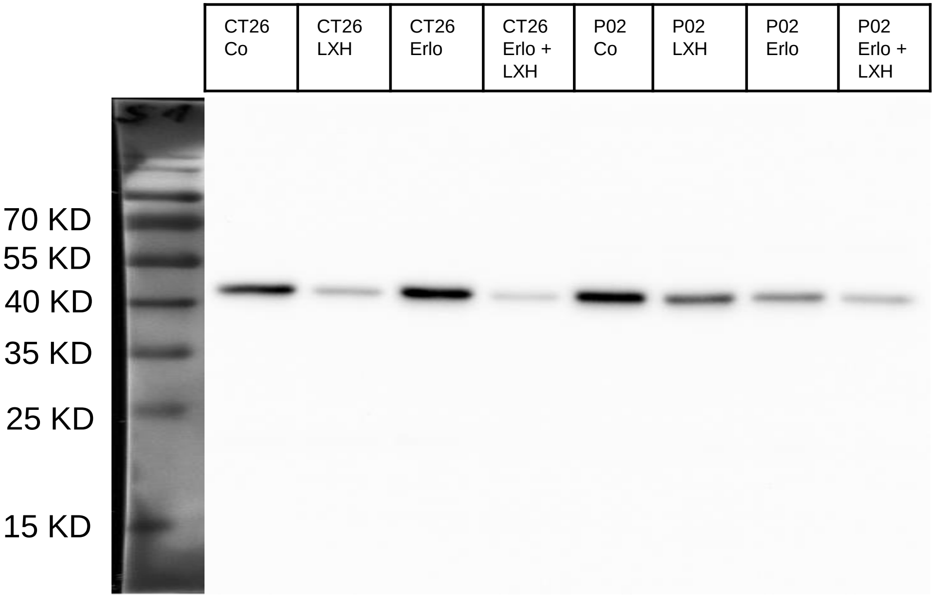

Mek (CT-26/ Panc02 cells + 1 µM LXH-254, 1 µM Erlotinib, 29.07.2022)

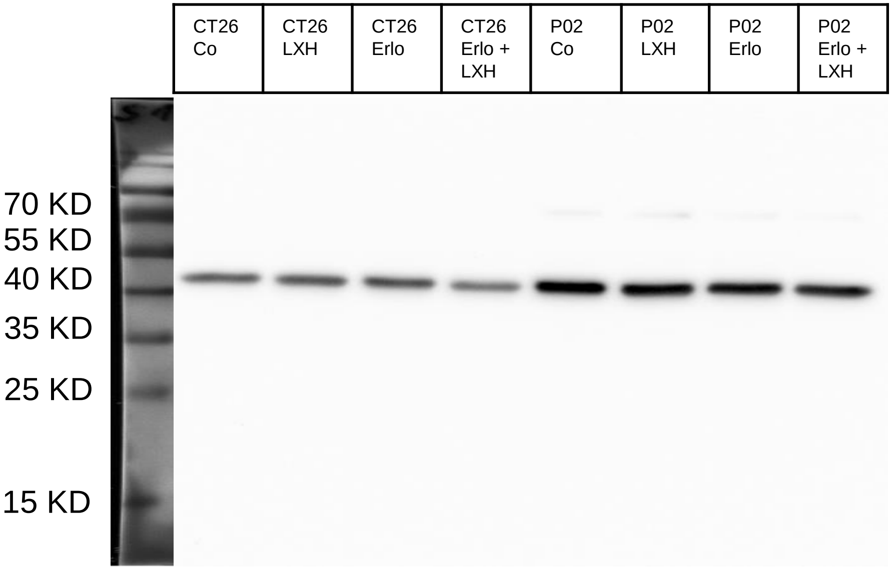

β-actin (pMEK/MEK, Panc02-Zellen + 1 µM LXH-254, 1 µM Erlotinib, 01.08.2022)

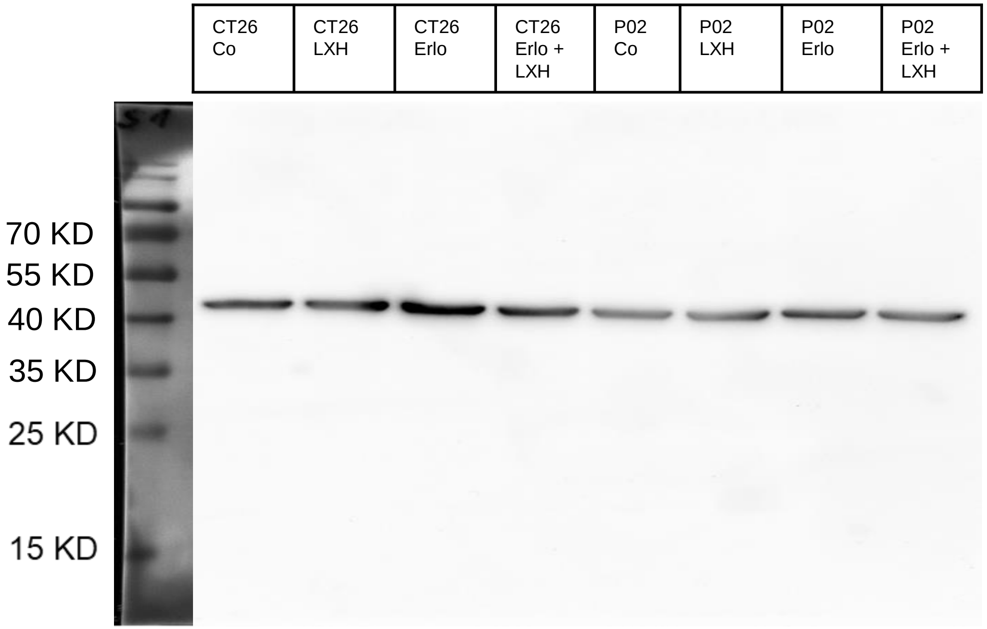

pERK (CT-26 / Panc02 cells + 1 µM LXH-254, 1 µM Erlotinib, 28.07.2022)

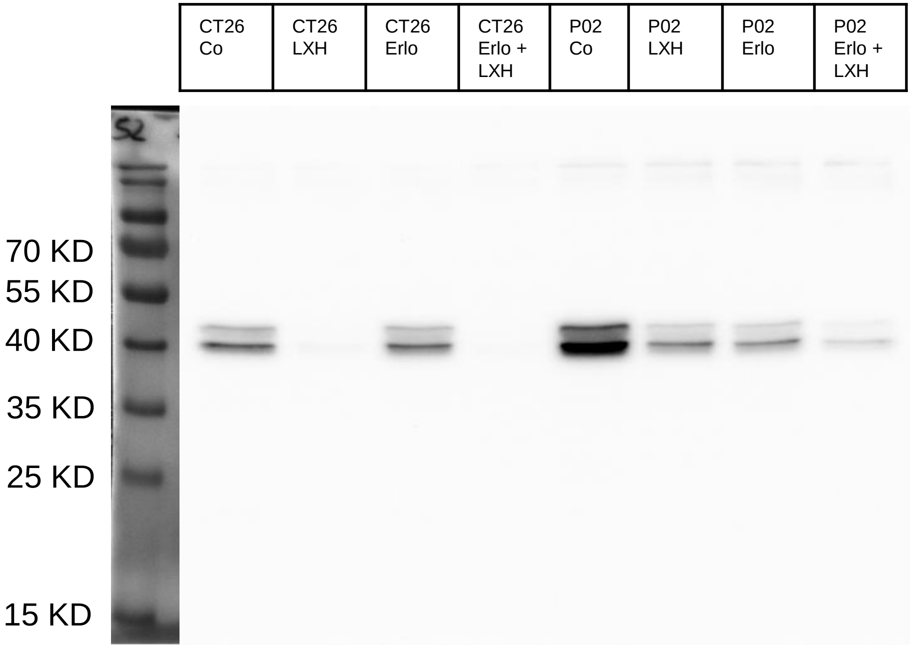

ERK (CT-26/ Panc02 cells + 1 µM LXH-254, 1 µM Erlotinib, 29.07.2022)

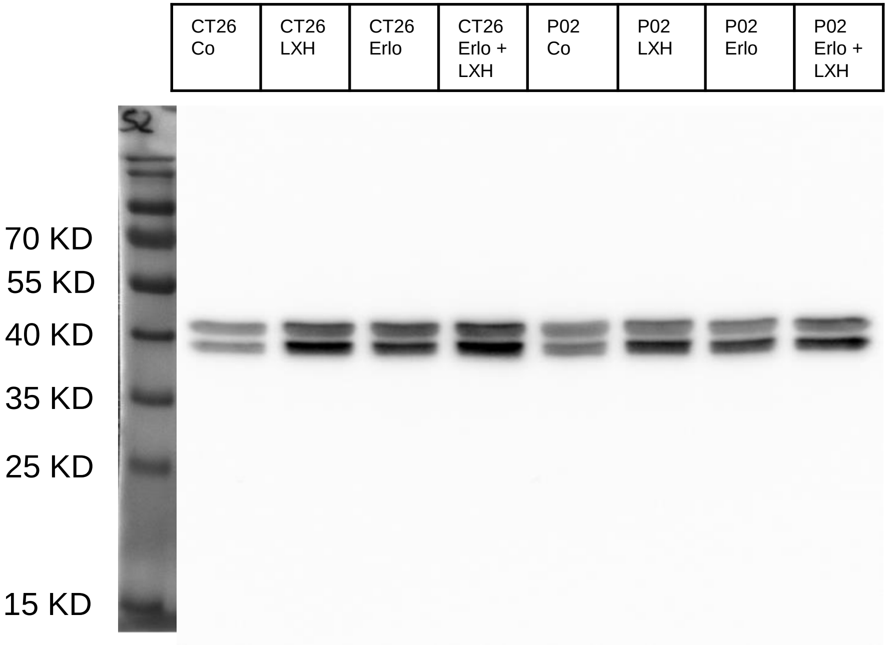

β-actin (pERK/ERK, Panc02-Zellen + 1 µM LXH-254, 1 µM Erlotinib, 01.08.2022)

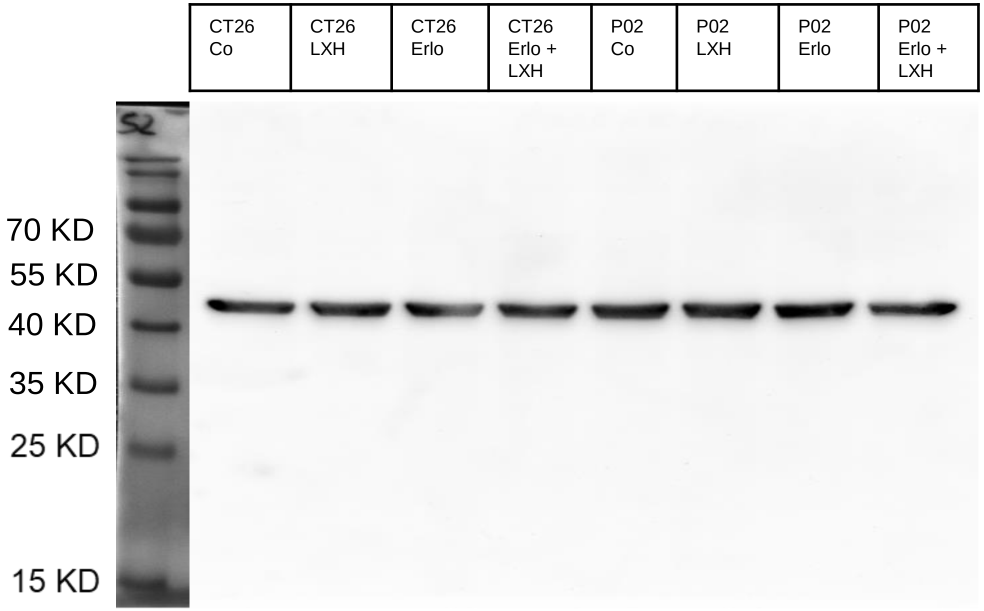

pEGFR (CT-26 / Panc02 cells + 1 µM LXH-254, 1 µM Erlotinib, 02.08.2022)

EGFR (CT-26/ Panc02 cells + 1 µM LXH-254, 1 µM Erlotinib, 03.08.2022)

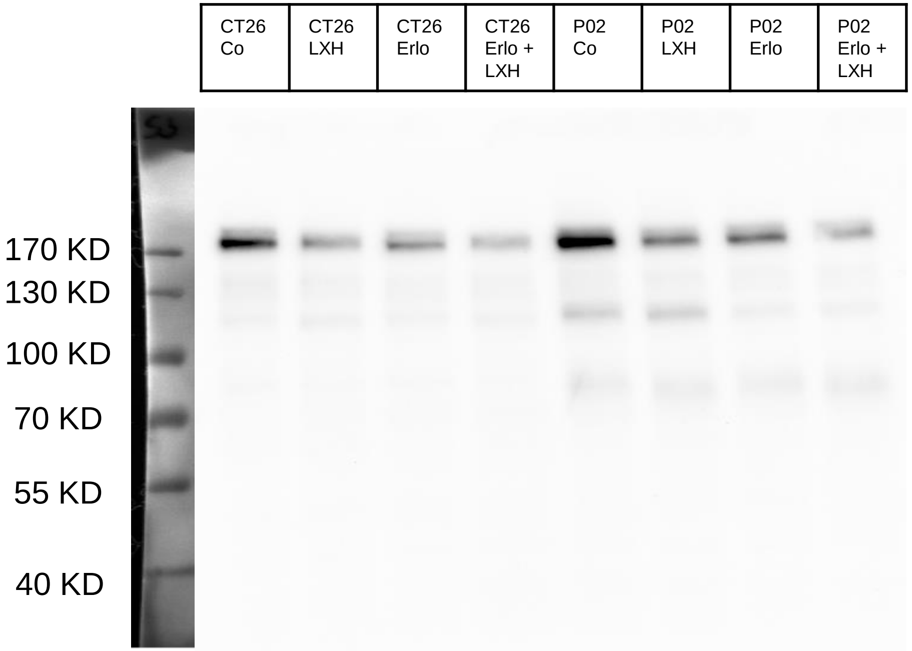

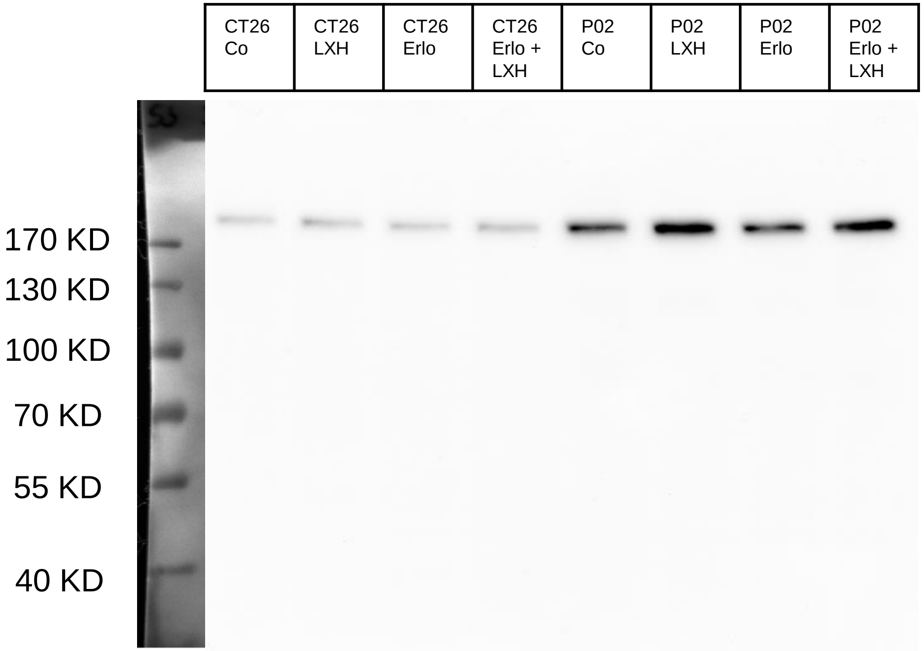

β-actin (pEGFR/EGFR, Panc02-Zellen + 1 µM LXH-254, 1 µM Erlotinib, 04.08.2022)

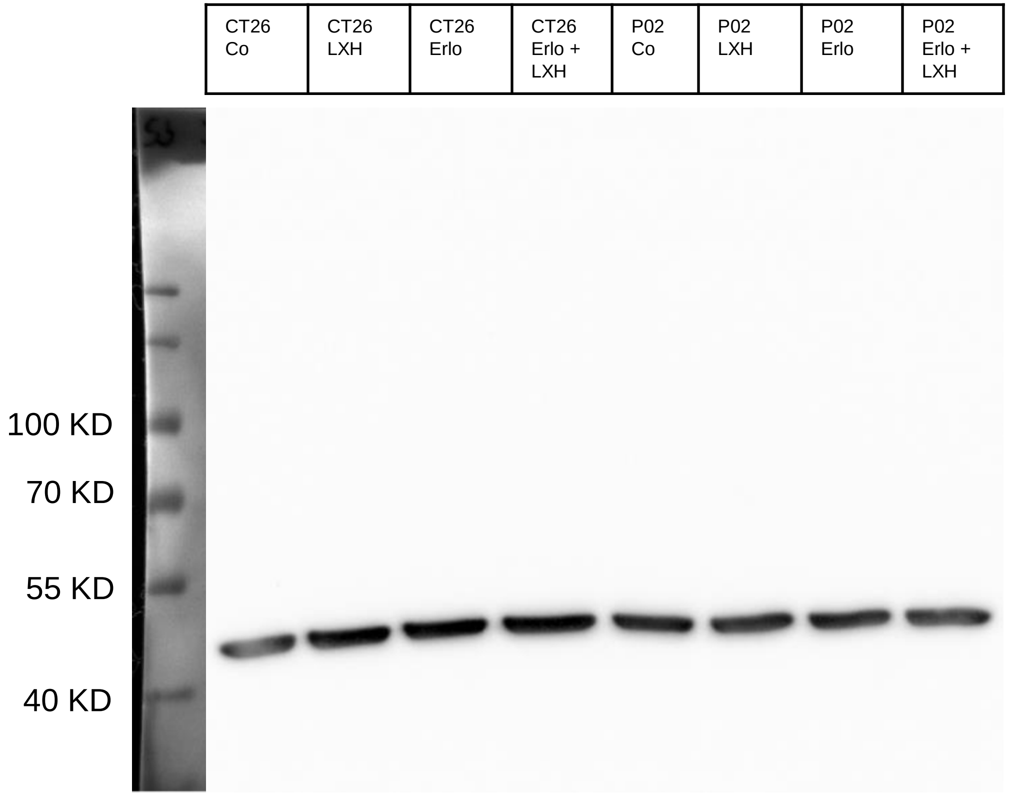

pMEK (CT-26 / Panc02 cells + 1 µM LXH-254, 1 µM Erlotinib, 02.08.2022)

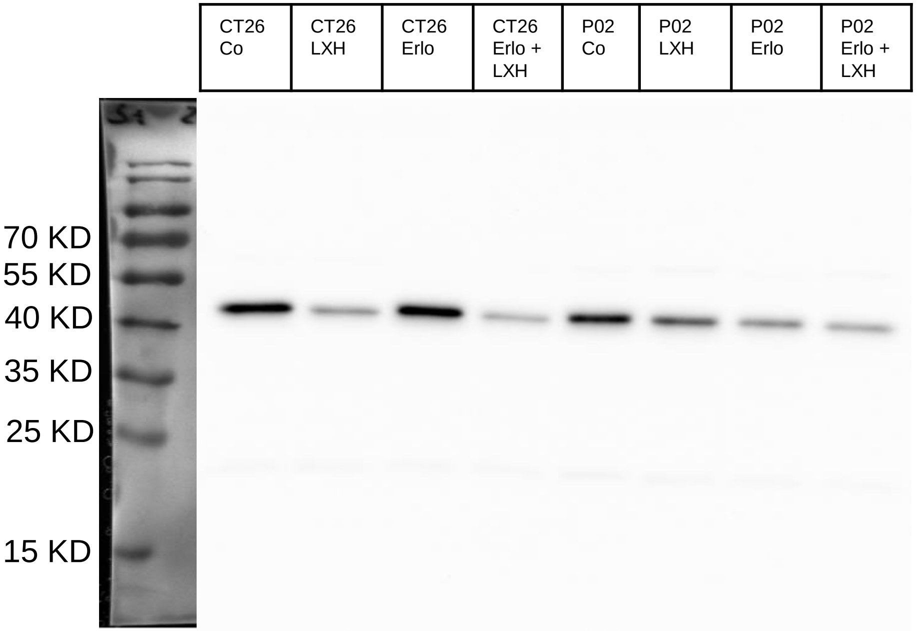

MEK (CT-26/ Panc02 cells + 1 µM LXH-254, 1 µM Erlotinib, 03.08.2022)

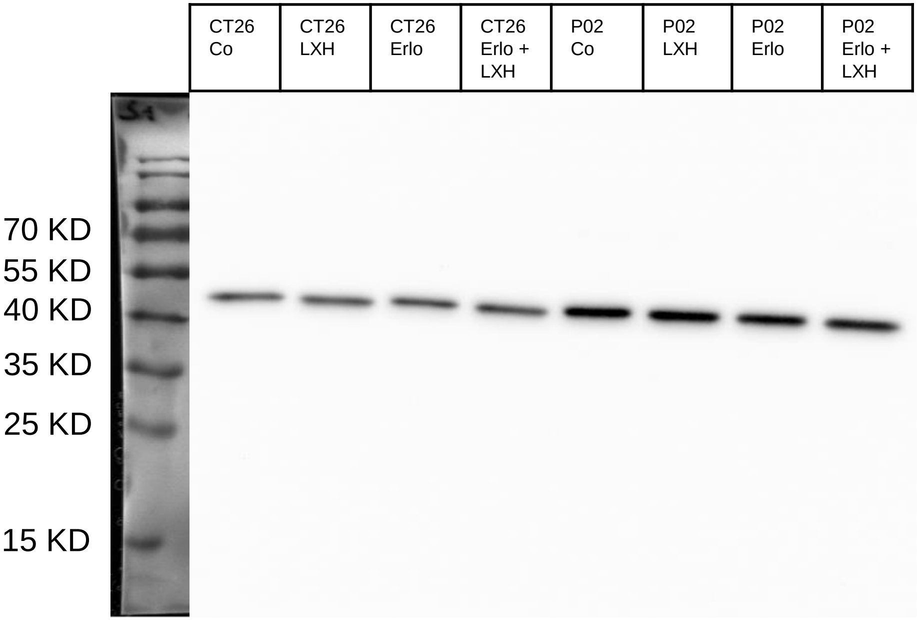

β-actin (pMek/Mek, Panc02-Zellen + 1 µM LXH-254, 1 µM Erlotinib, 04.08.2022)

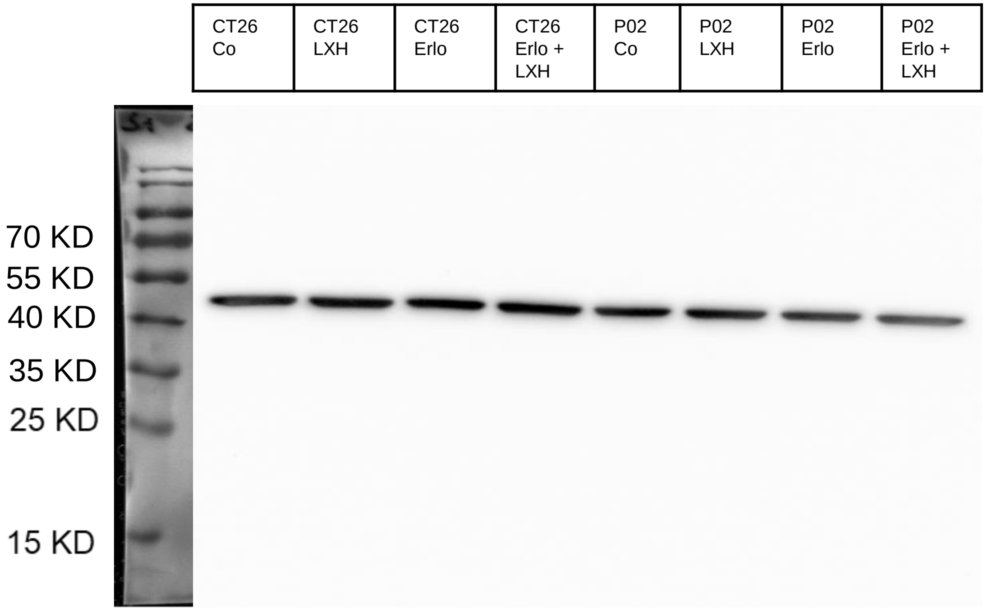

pERK (CT-26 / Panc02 cells + 1 µM LXH-254, 1 µM Erlotinib, 02.08.2022)

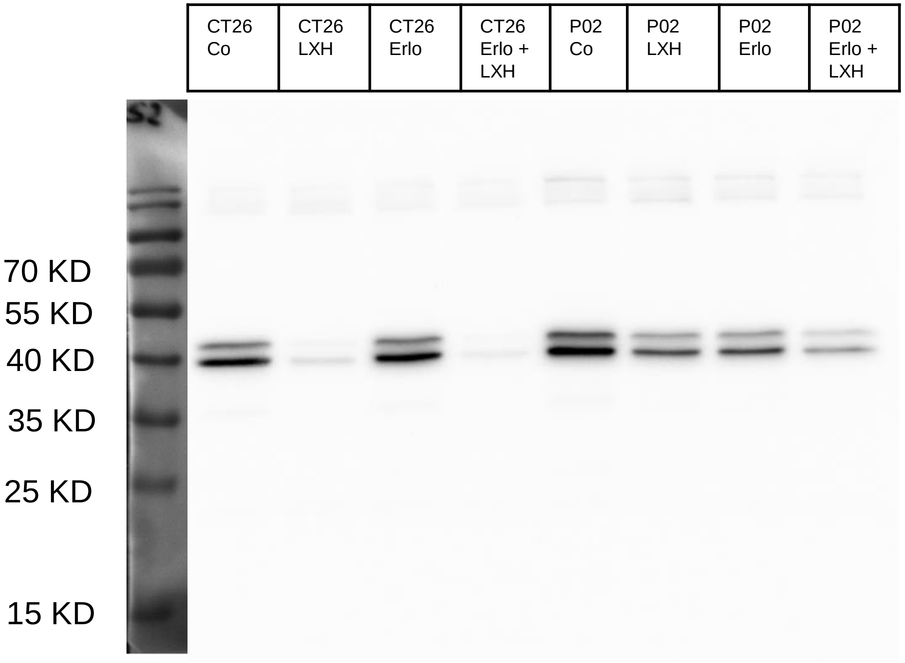

ERK (CT-26/ Panc02 cells + 1 µM LXH-254, 1 µM Erlotinib, 03.08.2022)

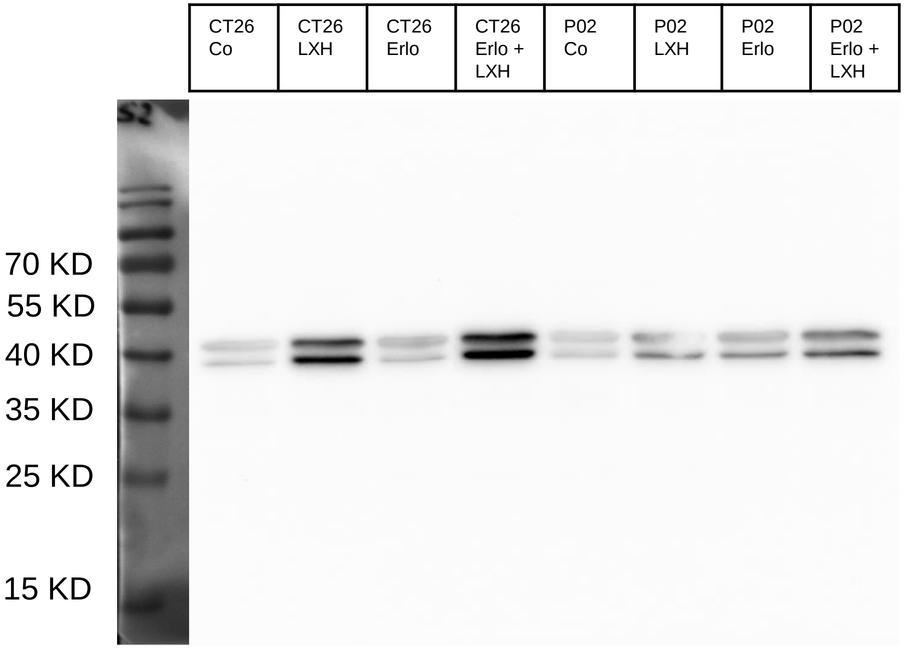

β-actin (pERK/ERK, Panc02-Zellen + 1 µM LXH-254, 1 µM Erlotinib, 04.08.2022)

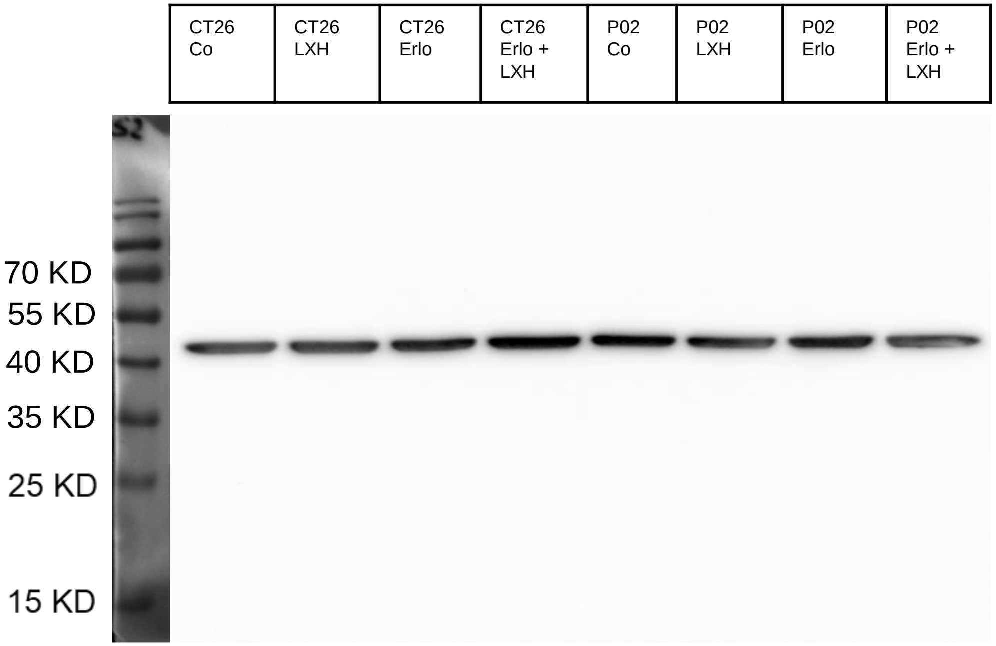

pEGFR (CT-26 / Panc02 cells + 1 µM LXH-254, 1 µM Erlotinib, 05.10.2022)

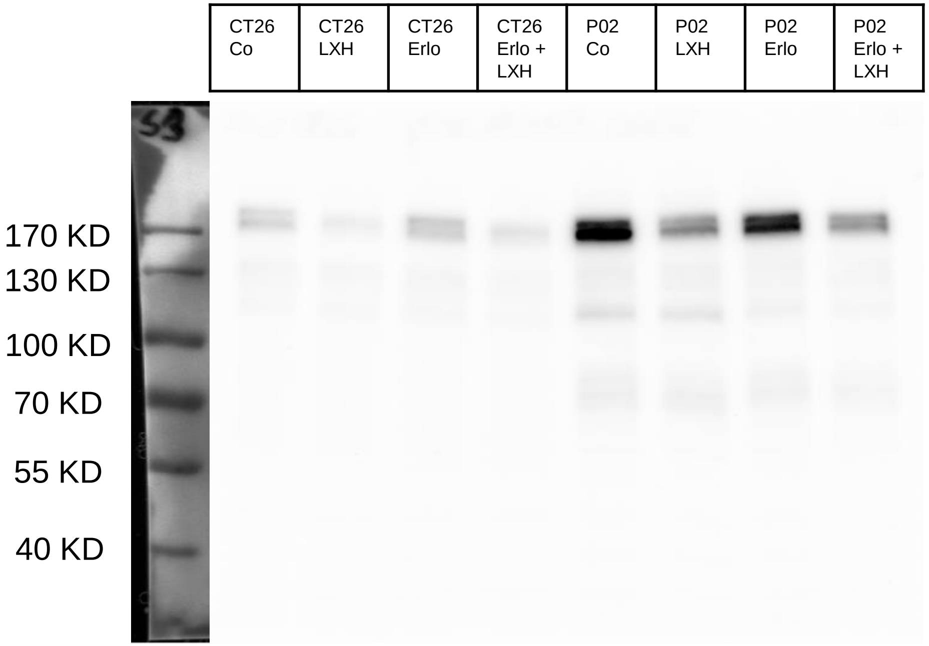

EGFR (CT-26/ Panc02 cells + 1 µM LXH-254, 1 µM Erlotinib, 06.10.2022)

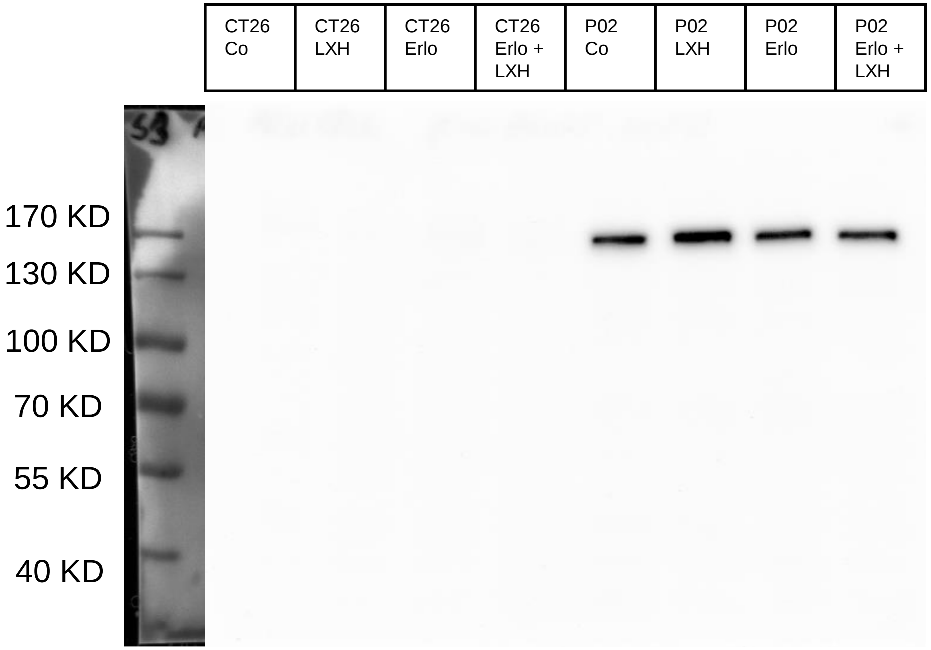

β-actin (pEGFR/EGFR, Panc02-Zellen + 1 µM LXH-254, 1 µM Erlotinib, 07.10.2022)

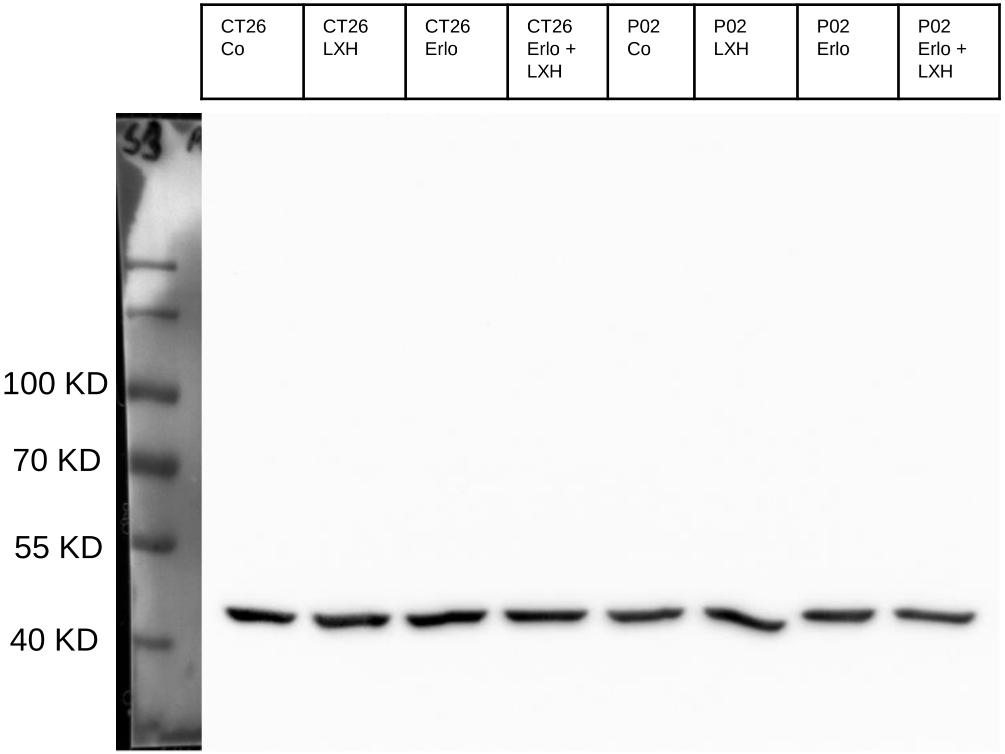

pMEK (CT-26 / Panc02 cells + 1 µM LXH-254, 1 µM Erlotinib, 05.10.2022)

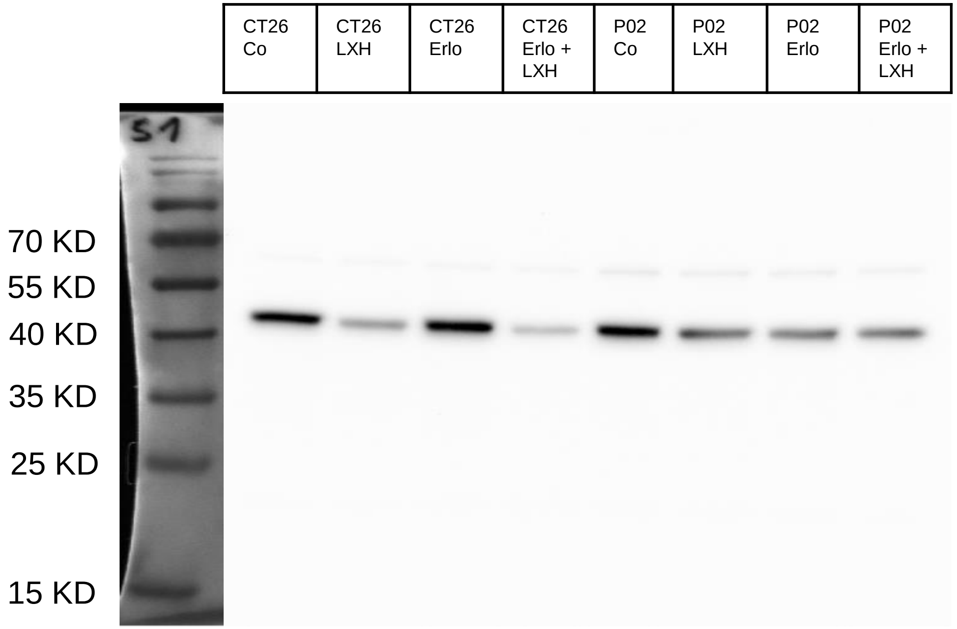

MEK (CT-26/ Panc02 cells + 1 µM LXH-254, 1 µM Erlotinib, 06.10.2022)

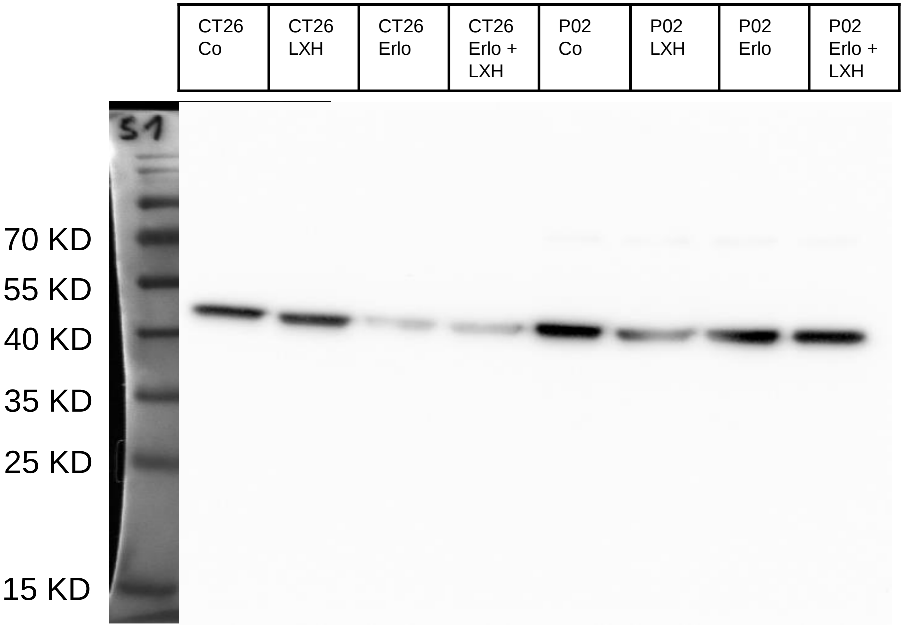

β-actin (pMEK/MEK, Panc02-Zellen + 1 µM LXH-254, 1 µM Erlotinib, 07.10.2022)

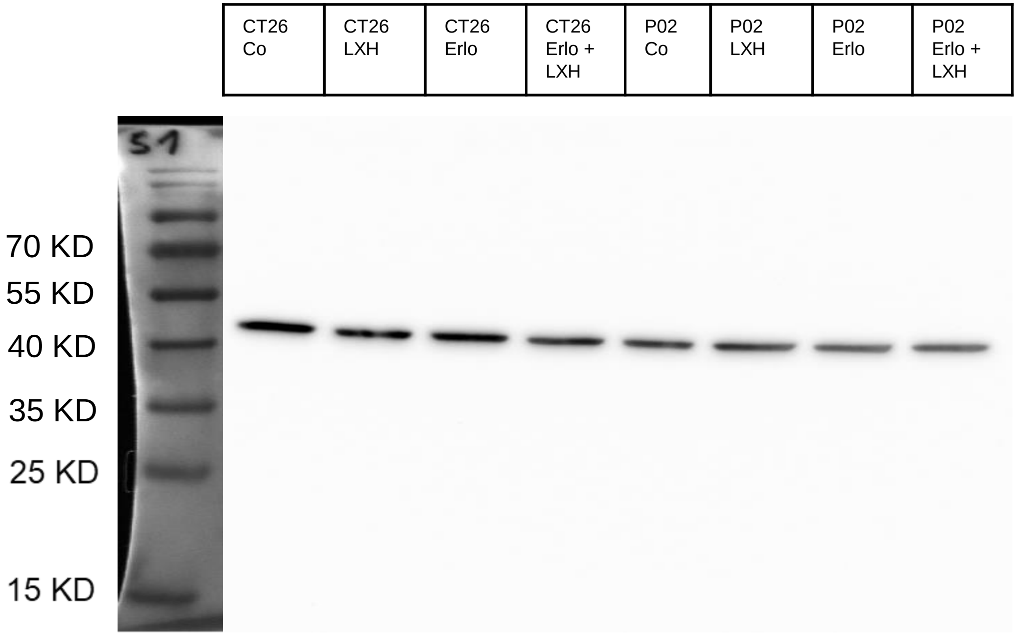

pERK (CT-26 / Panc02 cells + 1 µM LXH-254, 1 µM Erlotinib, 05.10.2022)

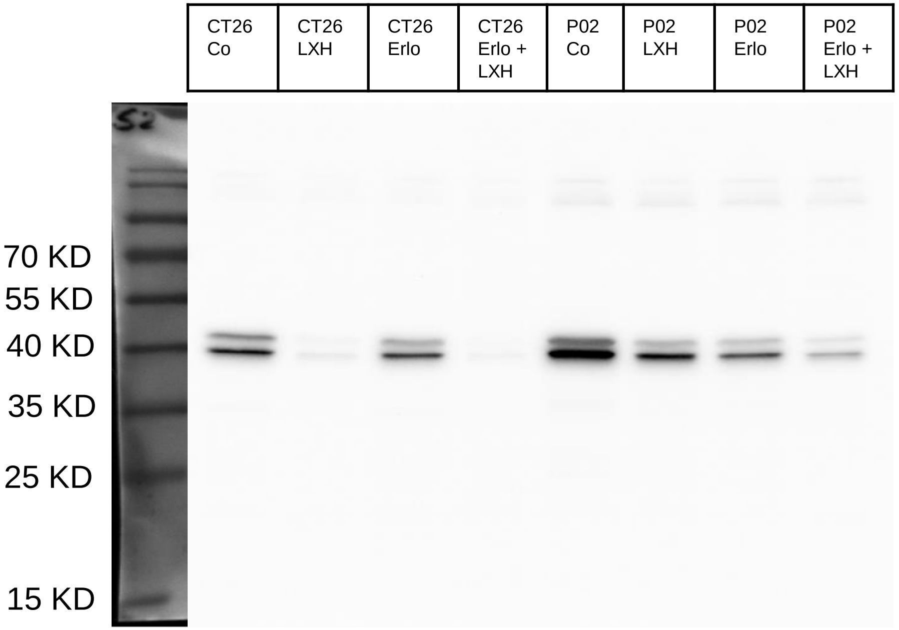

ERK (CT-26/ Panc02 cells + 1 µM LXH-254, 1 µM Erlotinib, 06.10.2022)

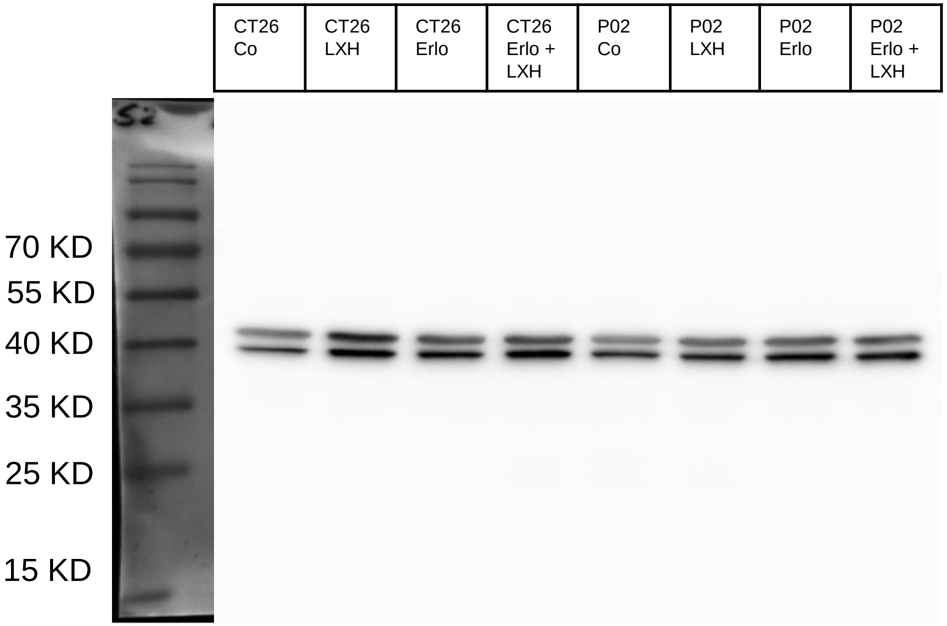

β-actin (pERK/ERK, Panc02-Zellen + 1 µM LXH-254, 1 µM Erlotinib, 07.10.2022)

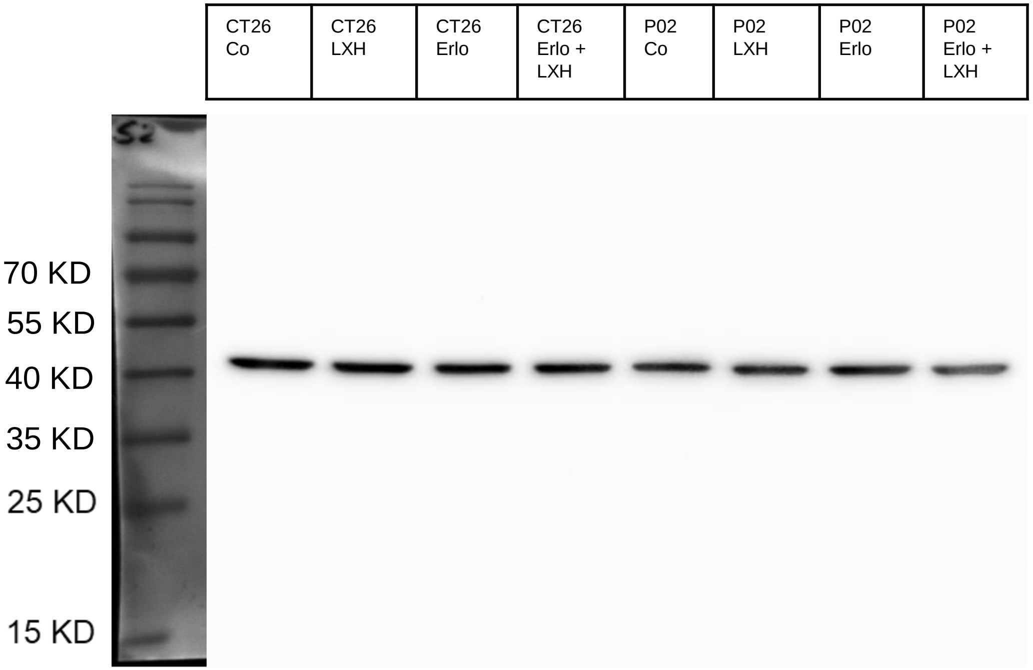

pEGFR (CT-26 / Panc02 cells + 1 µM LXH-254, 1 µM Erlotinib, 18.10.2022)

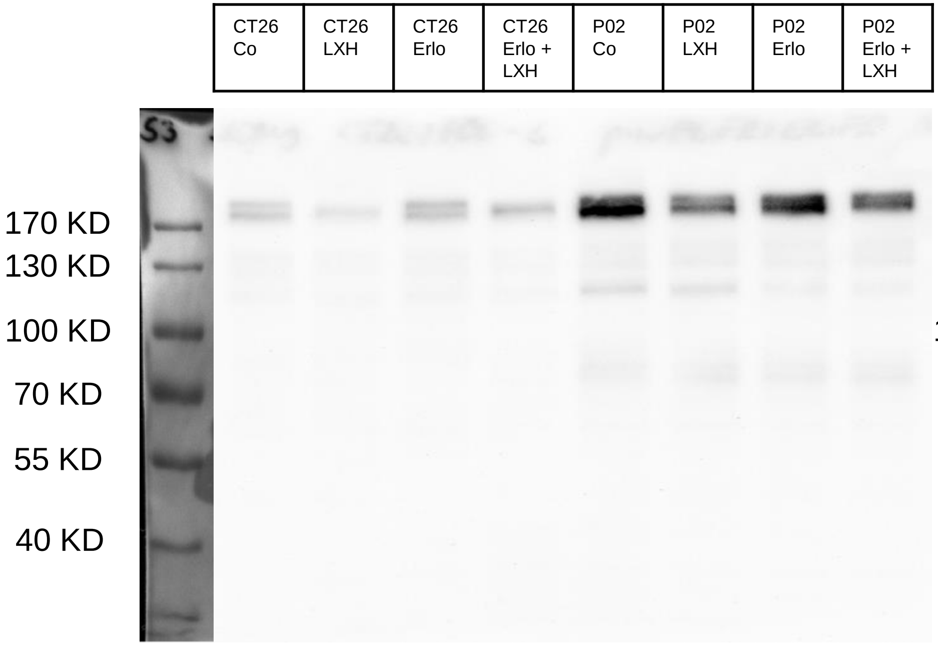

EGFR (CT-26/ Panc02 cells + 1 µM LXH-254, 1 µM Erlotinib, 19.10.2022)

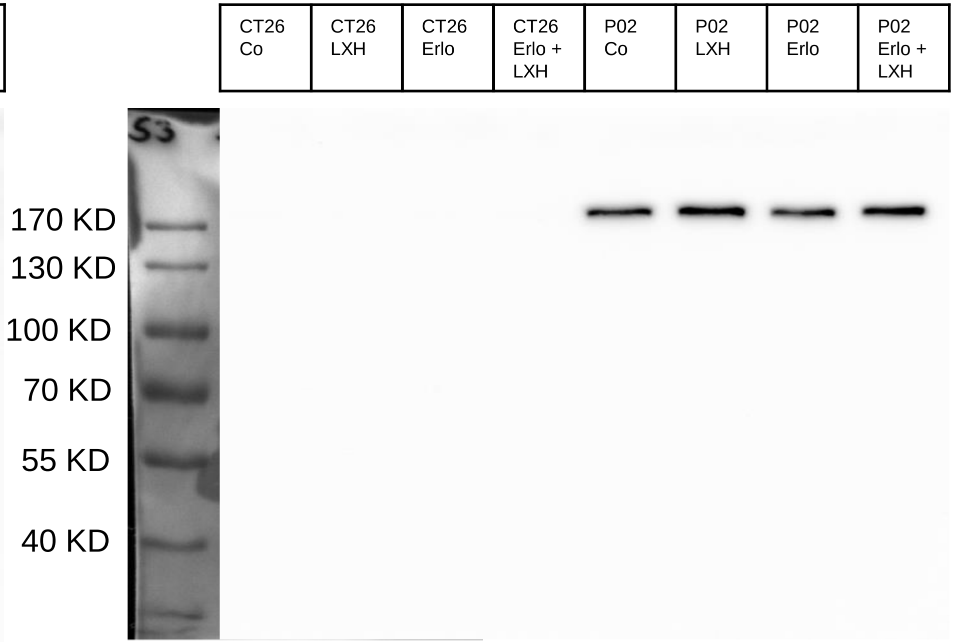

β-actin (pEGFR/EGFR, Panc02-Zellen + 1 µM LXH-254, 1 µM Erlotinib, 20.10.2022)

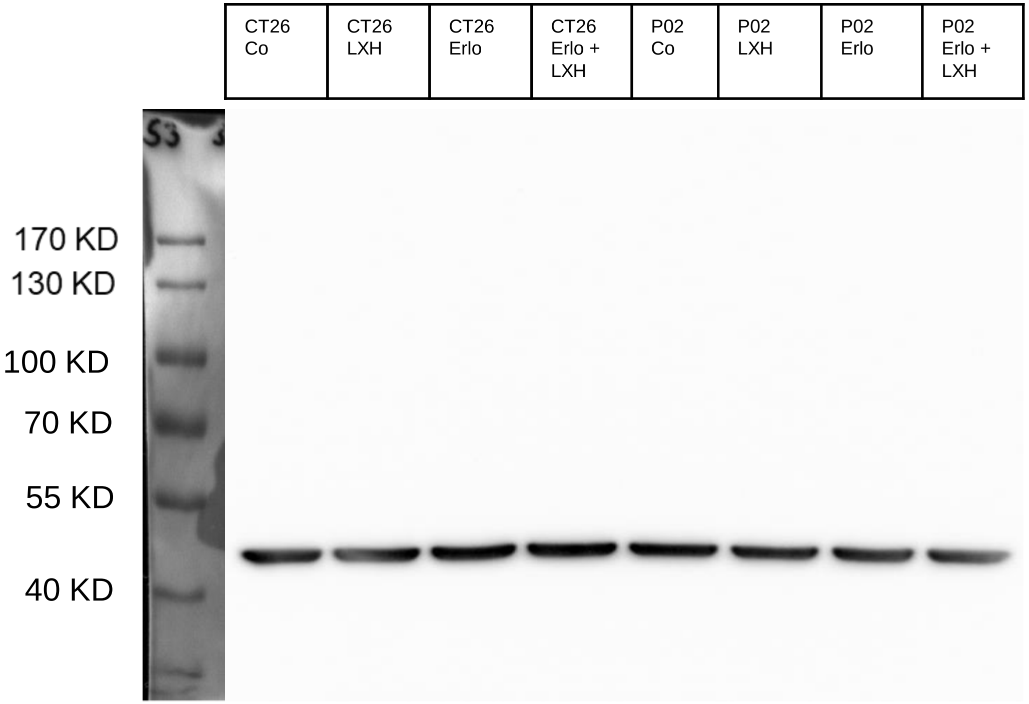

pMEK (CT-26 / Panc02 cells + 1 µM LXH-254, 1 µM Erlotinib, 18.10.2022)

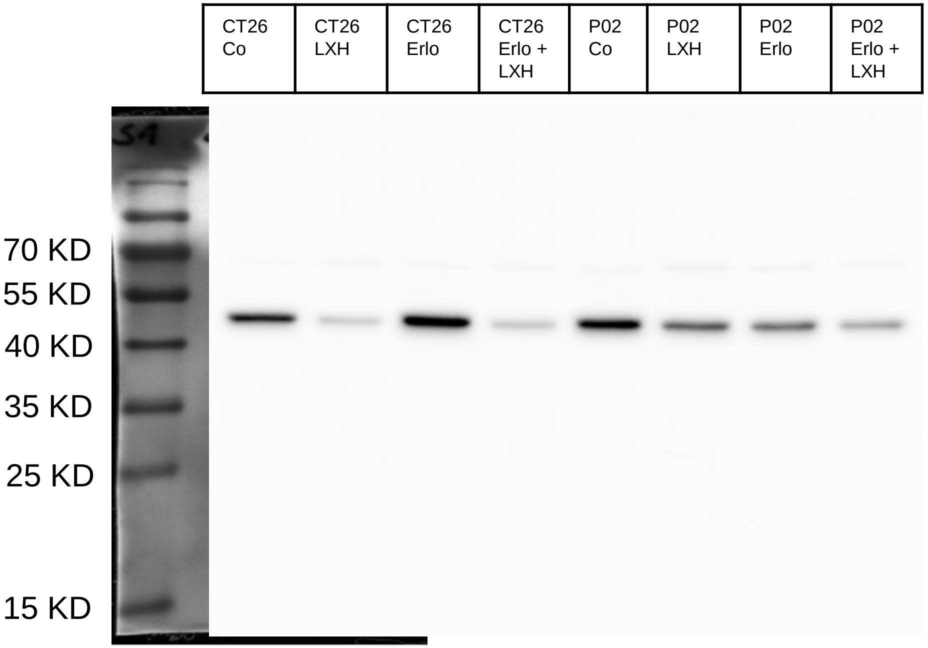

MEK (CT-26/ Panc02 cells + 1 µM LXH-254, 1 µM Erlotinib, 19.10.2022)

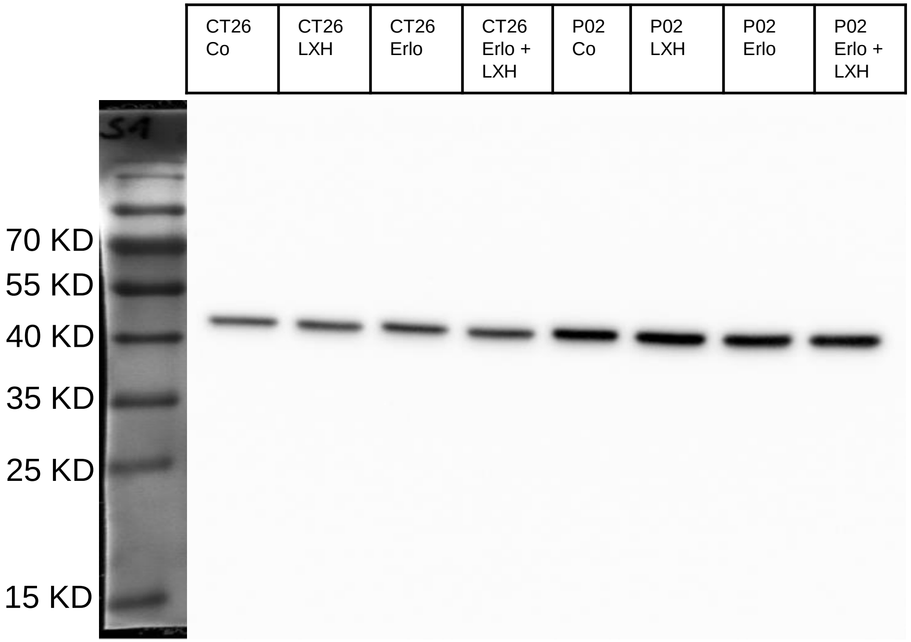

β-actin (pMEK/MEK, Panc02-Zellen + 1 µM LXH-254, 1 µM Erlotinib, 20.10.2022)

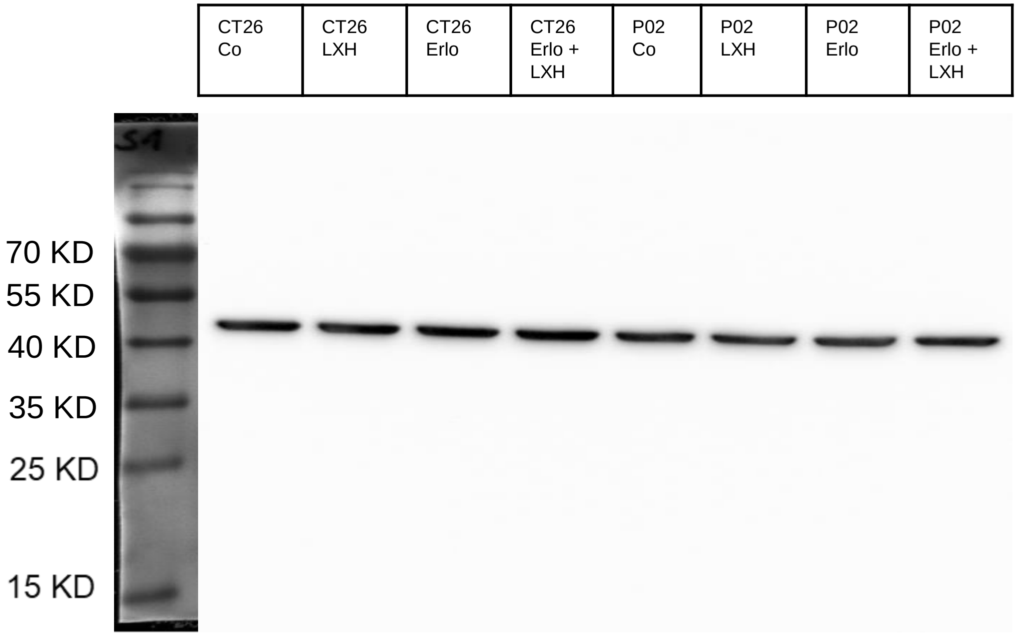

pERK (CT-26 / Panc02 cells + 1 µM LXH-254, 1 µM Erlotinib, 18.10.2022)

ERK (CT-26/ Panc02 cells + 1 µM LXH-254, 1 µM Erlotinib, 19.10.2022)

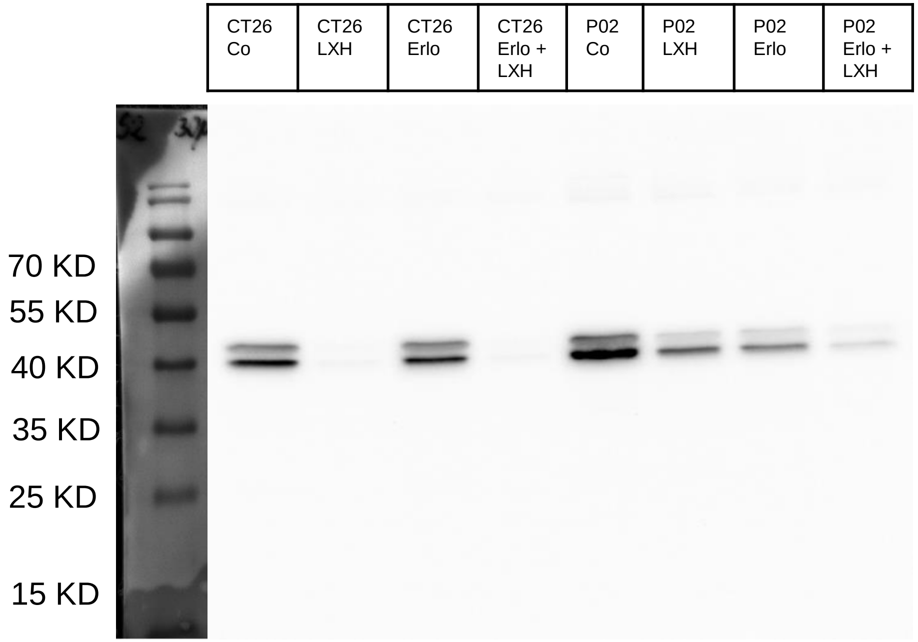

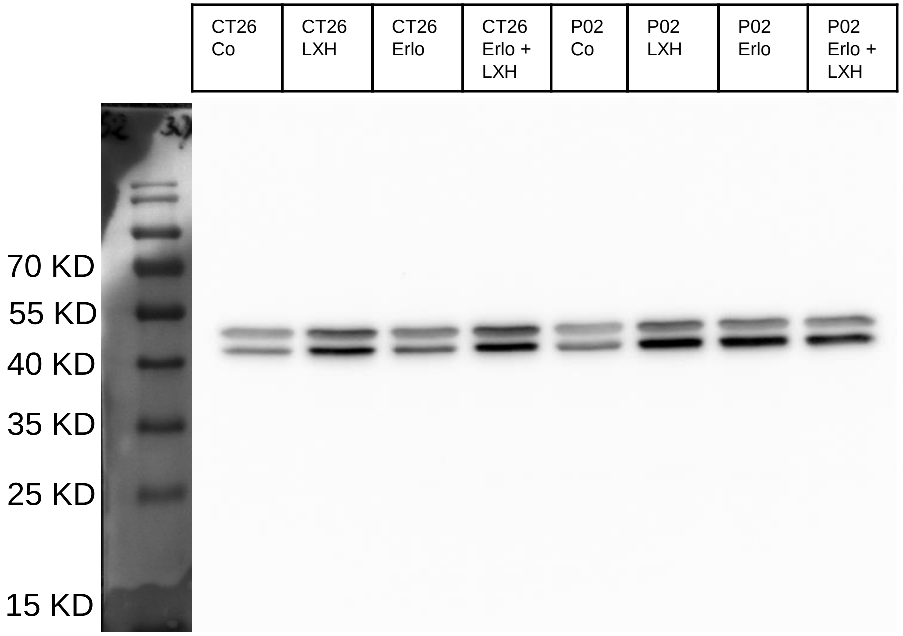

β-actin (pERK/ERK, Panc02-Zellen + 1 µM LXH-254, 1 µM Erlotinib, 20.10.2022)

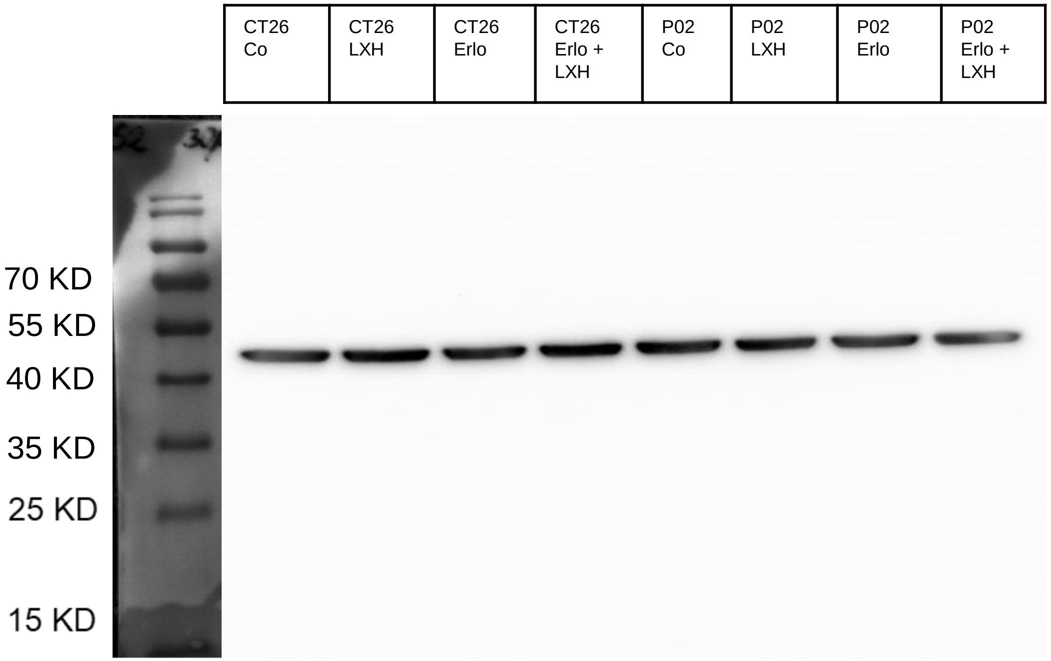

Supplement: S7 Fig — (PDF) [file pone.0347843.s007.pdf]
