# Supplementary material for: Targeting pancreatic cancer with combined inhibition of EGFR and RAF
Source: PLoS One. 2026 Apr 24;21(4):e0347843. doi: 10.1371/journal.pone.0347843 (PMC13108728; doi:10.1371/journal.pone.0347843)
Supplement: Attachment — Submitted filename: Response to Reviewer.pdf [file pone.0347843.s010.pdf]

**Reviewer 1:**

**<u>Comment 1:</u>**

The paper of Brandstetter et al. mainly shows negative in vivo therapeutic results, validating the previous data of Blasco et al., that obtained therapeutic effect by genetic but not by pharmacologic strategies against EGFR and Raf1. The work merits publication if they need to include the following information:

The fact that in vivo survival is lower under the treatment regimen (erlotinib and LXH-254) suggest that the in vitro results were not therapeutic but toxic. This information should be included in the discussion.

**<u>Answer to Comment 1:</u>**

The reviewer is correct that we did not see a therapeutic effect of the combination therapy. In contrast, mice treated with LXH-254 and erlotinib exhibited significantly reduced survival compared with vehicle-treated controls. This observation could suggest a toxic effect of the therapy. However, we did not detect evidence of a direct systemic toxicity attributable to the treatment.

Throughout the experiment, we performed a comprehensive distress assessment, including measurements of body weight change, perianal temperature, distress score, mouse grimace scale, corticosterone concentration in the plasma and its metabolites in the feces. No significant differences between treatment groups were detected in these parameters (**SFig1 A-F**). Therefore, we were unable to detect any significant adverse health effects on the animals as a result of the therapy.

In addition, we analyzed complete blood counts during therapy and included these data as new **Supplementary Figure 3**. No significant differences were observed between treatment groups. The lymphocyte counts were reduced in all groups; however, lymphocytopenia is frequently observed during the progression of solid tumors and represents an adverse prognostic factor (Lee et al. 2019; Joseph et al. 2016). Accordingly, the hematological analysis did not reveal clear evidence of treatment-induced toxicity.

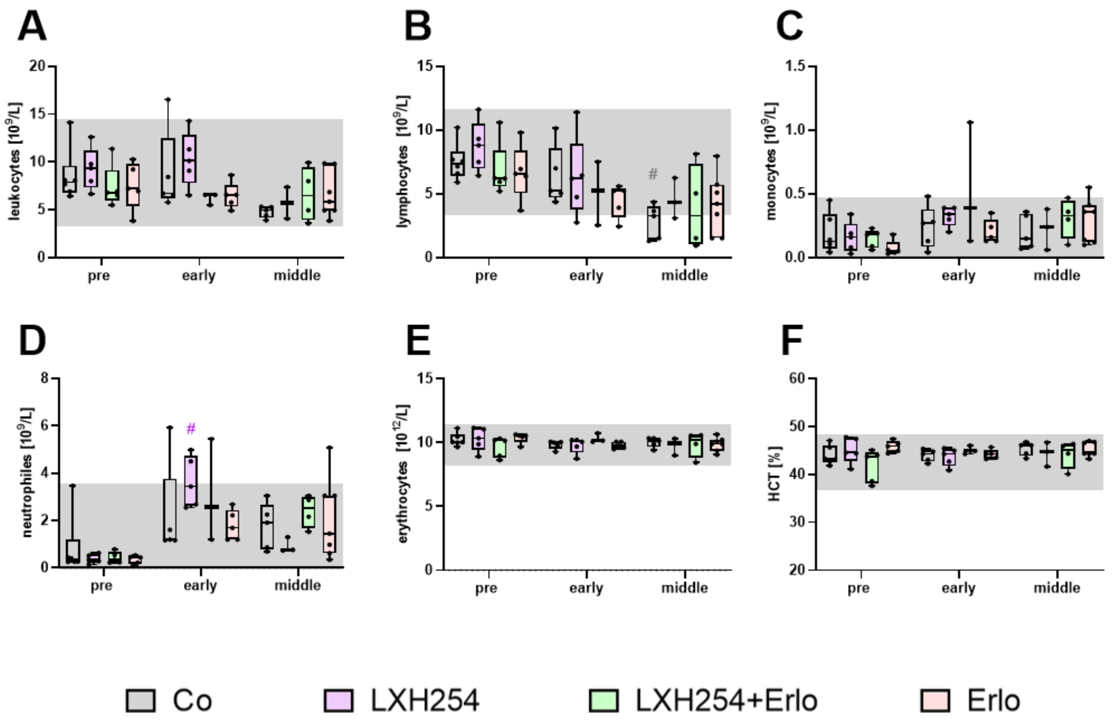


**S3 Fig. Blood count before and during the therapy of all treatment groups.** Counts of leukocytes (A), lymphocytes (B), monocytes (C), neutrophils (D), erythrocytes (E) and hematocrit (HCT; F) from healthy mice (pre) and during the early and middle phase of tumor progression and therapeutic intervention. Data were tested for differences between the groups (*p<0.05) as well as longitudinally compared to the baseline value (pre, #p<0.05). The grey area represents the base line measurements on healthy mice. Statistics were carried out using mixed-effects model. Control: n = 6; LXH-254: n = 6; LXH 254+erlotinib: n = 4-5; erlotinib: n = 6.

Nevertheless, we cannot exclude subtle or organ-specific toxic effects, as we did not perform extensive clinical chemistry or histopathological analyses for toxicological purpose. Importantly, no mouse required euthanasia due to suspected treatment-related toxicity. The endpoint criteria were >15% body weight loss and hunched posture, which occurred as consequence of tumor progression, specifically intestinal invasion. Based on these observations, we refrain from classifying the combinatorial treatment as directly toxic. However, the treatment was associated with accelerated tumor progression and growth into the intestinal lumen and should therefore be considered harmful in this model and under the dosage used. In addition to the new figure (S3 Fig**.**) we also changed the text in the discussion accordingly in line 454- 458 of the revised manuscript: “Therefore, we did not quantify anti-cancer effects of the combinatorial treatment. In contrast, mice treated with LXH-254 and erlotinib exhibited significantly reduced survival compared with vehicle-treated controls (Fig 3D). Which might indicate a possible toxic effect of the combinatorial treatment. We cannot exclude subtle or organ-specific toxic effects, as we did not perform extensive clinical chemistry or histopathological toxicology analyses.” Additional discussion of this aspect was addressed in line 474-786:” In addition to the low side effect profile quantified by the welfare parameters, we did not quantify severe changes of the blood counts except for downregulation of lymphocytes during the therapeutic intervention, especially in control-treated mice (S3 Fig). However, lymphocytopenia is frequently observed during the progression of solid tumors and represents an adverse prognostic factor (Lee et al. 2019; Joseph et al. 2016). In addition, the neutrophils were significantly upregulated in LXH-254-treated animals (S3 Fig). Importantly, no mouse required euthanasia due to suspected treatment related toxicity. The endpoint criteria were body weight loss (>15%) and hunched body posture which occurred as consequence of tumor progression since most animals (17 out of 25; 68%) exhibited invasive tumor growth into

the intestinal lumen. Based on these observations, we refrain from classifying the combinatorial treatment as directly toxic. However, the treatment was associated with accelerated tumor progression and increased intestinal invasion and should therefore be considered harmful in this model and under the used dosing conditions."

**Comment 2:**

Studies in Barbacid´s lab using different RAF kinase inhibitors (panRAF inhibitors MLN2480 and LSN3074753, a RAF1 selective inhibitors GW5074, and the paradox breaker PLX8394), have failed to show significant therapeutic activity on Kras/Trp53 mutant lung and PDAC tumors. The reason for this lack of therapeutic activity has been unveiled by the studies that have demonstrated that expression of two independent kinase dead isoforms of RAF1 (RAF1K375M and RAF1D468A) failed to show any relevant anti-tumor activity, thus indicating that the therapeutic activity of RAF1 observed in GEM tumor models of lung and pancreatic cancer is not mediated by its kinase activity, a truly unexpected result (Esteban-Burgos L, et al. Proc Natl Acad Sci U S A. 2020;117(39):24415-26). Instead, the results suggest that the anti-tumor activity observed upon RAF1 ablation is due to the elimination of its anti-apoptotic activity (Sanclemente M, et al. Cancer Cell. 2021;39(3):294-6.). This information should be included in the discussion or introduction.

**Answer to Comment 2:**

We thank the reviewer for this important aspect on the RAF1 inhibition. We added this aspect and cited the specific literature in the discussion line 549-558 and line 568-69 of the revised manuscript: "Recently Sanclemente et al. used mouse strains that expressed knock-in alleles of two kineases-dead RAF1 variants (RAF1D468A, RAF1K375M) to demonstrate that the therapeutic benefit of RAF-1 ablation in KRAS/TRP53 mutant lung tumors is not mediated by inhibition of its kinase catalytic activity, but rather by the loss of its anti-apoptotic functions (Sanclemente et al. 2021). RAF-1 has been shown to interact with pro-apoptotic the kinases ASK1 and MST2 (Chen et al. 2001; O'Neill et al. 2004), thereby suppressing apoptotic signaling. Consequently, instead of directly targeting of RAF1 kinase activity, future therapeutic strategies should focus on disrupting its anti-apoptotic, either by blocking its interaction with ASK1 and MST2 or by suppressing RAF-1 expression using selective RAF-1 degraders (Sanclemente et al. 2021).

Increased PI3K activity and hypermethylation of several tumor suppressors were also described as mechanisms in CDK4/RAF1 resistant lung adenocarcinomas (Esteban-Burgos et al. 2020)."

**Comment 3:**

One of the resistant mechanisms described in Blasco et al. and recently in https://doi.org/10.1101/2025.08.04.668325 is the activation of STAT3. The authors should compare the levels of pSTAT3 in the tumors treated in vivo with vehicle or with erlotinib and LXH-254.

## Answer to Comment 3:

According to the reviewer's suggestions we analyzed the pSTAT3 expression in all of our tumors. In addition to pSTAT3 we also quantified the necrotic area, proliferating cells and apoptotic cells in the tumors. These histological analyses are indicated in the new supplementary Figures S5 and S6.

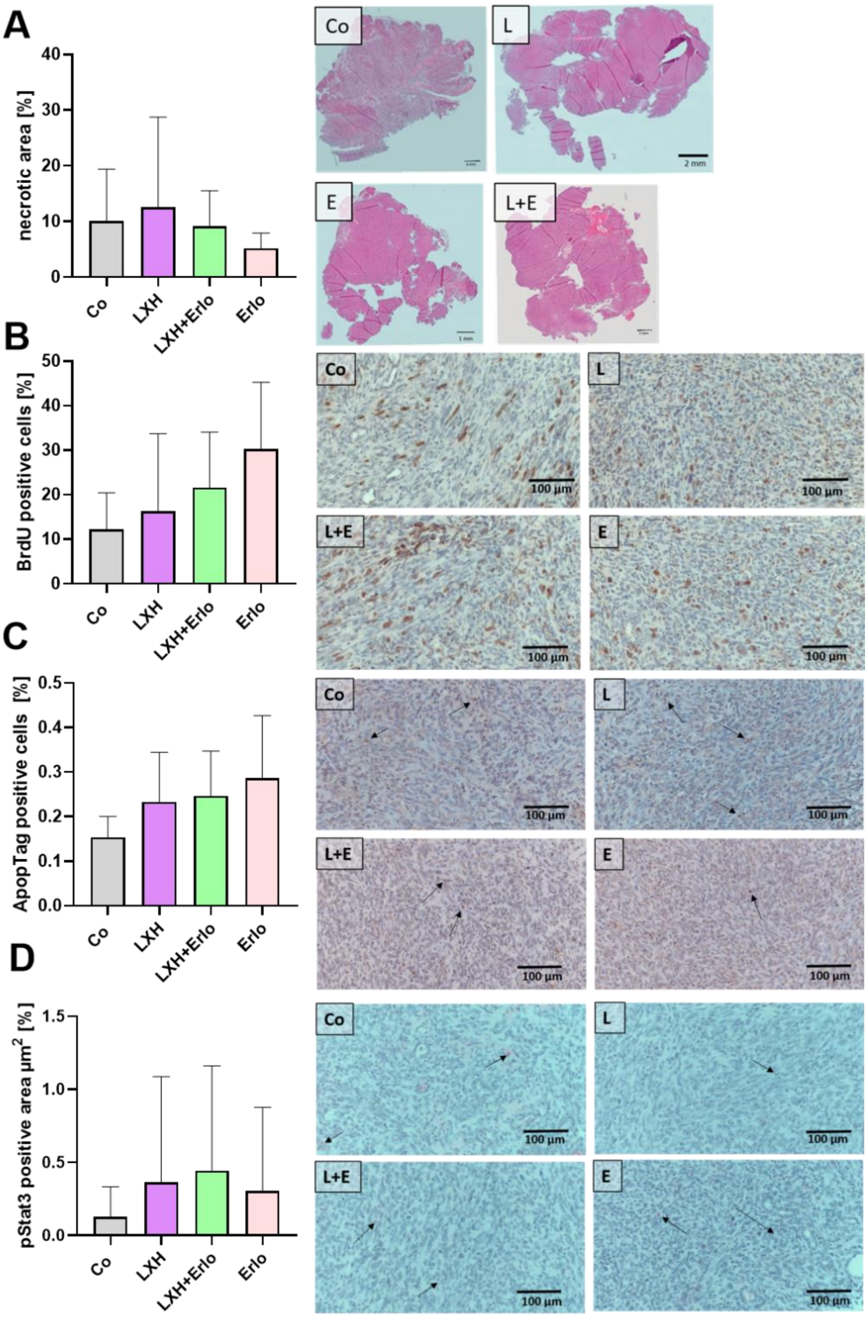


**S5 Fig. Immunohistological analysis of tumors from treated C57BL/6J mice.** Quantification of percentages of necrotic area via H/E staining (**A**), proliferating cells by BrdU incorporation (**B**), apoptotic cells via ApopTag® Peroxidase (**C**) and pSTAT3 positive area (**D**), respectively for control, LXH-254, LXH-254 plus erlotinib, or erlotinib treated mice. All staining's are illustrated in one exemplary picture for each treatment group. Arrows marking the specific stained cells, or area. Statistics were carried out using one-way ANOVA with Tukey-test for multiple comparisons. Co: n=5-6, LXH: n=3-6, LXH+Erlo n=3-4, Erlo n=4-7.

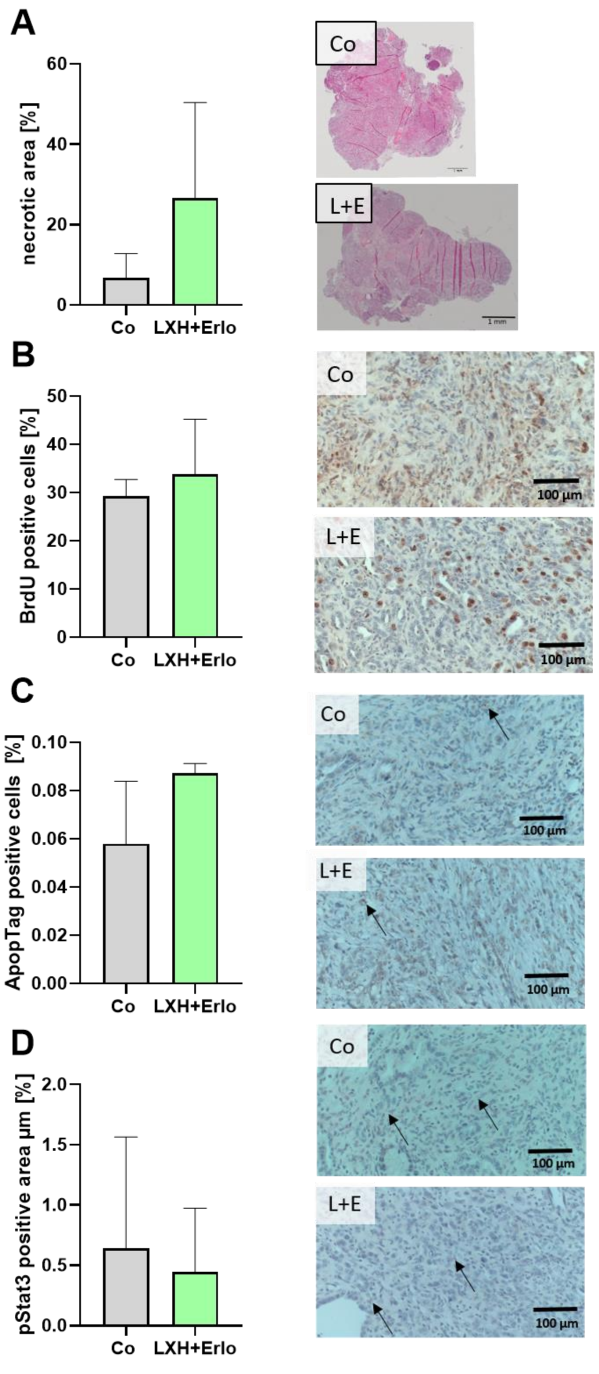


**S6 Fig. Immunohistological analysis of tumors from treated KPC mice.** Quantification of percentages of necrotic area via H/E staining (**A**), proliferating cells by BrdU incorporation (**B**), apoptotic cells via ApopTag® Peroxidase (**C**) and pSTAT3 positive area (**D**), respectively for control or LXH-254 plus erlotinib treated KPC mice. Arrows are marking the positive cells or area. Statistics were carried out using Mann-Whitney test; Co: n=3, LXH+Erlo n=2-3.

No significant differences were observed among the treatment groups for any of these parameters, including necrotic area, cell proliferation, apoptosis, or pSTAT3 positivity in either pancreatic cancer model (**S5D and S6D Figs.**). Although tumors from the treated groups in C57BL/6J mice (**S5D Fig.**) showed a trend toward a greater pSTAT3-positive area compared with control-treated animals, this difference was not statistically significant and the overall stained area was minimal. In addition, for the KPC mice the pSTAT3-positive area was slightly larger in control-mice, when compared to the combinatorial treatment (**S6D Fig.**). Therefore, we cannot conclude that increased activation of JAK-STAT pathway accounts for the observed therapeutic resistance. The interpretation of the histological

analyses is also very difficult, as the tumors were preserved at different days after therapy, at the individual humane endpoint of each mouse. We added the aspect in the results part line 408-412 and discussed this observation in line 451-453 of the revised manuscript. ”In addition to tumor weight and survival, histological analysis of the tumors, including assessment of necrotic area as well as proliferating and apoptotic cells also failed to reveal a detectable therapeutic effect of the treatment”. The results for pSTAT3 are discussed in line 532-538 of the revised manuscript. ”One important resistance mechanism after combined RAF and EGFR ablation was introduced from Blasco et al. by an increased activation of JAK-STAT signaling in resistant tumors (Blasco et al. 2019) . We did not quantify a significant increase of pSTAT3-positive area after combinatorial treatment of LXH-254 and erlotinib in both pancreatic cancer models (S5D and S6DFig.). Therefore, we cannot conclude that increased activation of JAK-STAT signaling pathway accounts for the observed therapeutic resistance”.

**References Reviewer 1**


Blasco, María Teresa; Navas, Carolina; Martín-Serrano, Guillermo; Graña-Castro, Osvaldo; Lechuga, Carmen G.; Martín-Díaz, Laura et al. (2019): Complete Regression of Advanced Pancreatic Ductal Adenocarcinomas upon Combined Inhibition of EGFR and C-RAF. In: *Cancer cell* 35 (4), 573-587.e6. DOI: 10.1016/j.ccell.2019.03.002.

Chen, J.; Fujii, K.; Zhang, L.; Roberts, T.; Fu, H. (2001): Raf-1 promotes cell survival by antagonizing apoptosis signal-regulating kinase 1 through a MEK-ERK independent mechanism. In: *Proceedings of the National Academy of Sciences of the United States of America* 98 (14), S. 7783–7788. DOI: 10.1073/pnas.141224398.

Esteban-Burgos, Laura; Wang, Haiyun; Nieto, Patricia; Zheng, Jie; Blanco-Aparicio, Carmen; Varela, Carmen et al. (2020): Tumor regression and resistance mechanisms upon CDK4 and RAF1 inactivation in KRAS/P53 mutant lung adenocarcinomas. In: *Proceedings of the National Academy of Sciences of the United States of America* 117 (39), S. 24415–24426. DOI: 10.1073/pnas.2002520117.

Joseph, N.; Dovedi, S. J.; Thompson, C.; Lyons, J.; Kennedy, J.; Elliott, T. et al. (2016): Pre-treatment lymphocytopaenia is an adverse prognostic biomarker in muscle-invasive and advanced bladder cancer. In: *Annals of oncology : official journal of the European Society for Medical Oncology* 27 (2), S. 294–299. DOI: 10.1093/annonc/mdv546.

Lee, Yong Jae; Chung, Young Shin; Lee, Jung-Yun; Nam, Eun Ji; Kim, Sang Wun; Kim, Sunghoon; Kim, Young Tae (2019): Pretreatment lymphocytopenia is an adverse prognostic biomarker in advanced-stage ovarian cancer. In: *Cancer medicine* 8 (2), S. 564–571. DOI: 10.1002/cam4.1956.

O'Neill, Eric; Rushworth, Linda; Baccarini, Manuela; Kolch, Walter (2004): Role of the kinase MST2 in suppression of apoptosis by the proto-oncogene product Raf-1. In: *Science (New York, N.Y.)* 306 (5705), S. 2267–2270. DOI: 10.1126/science.1103233.

Sanclemente, Manuel; Nieto, Patricia; Garcia-Alonso, Sara; Fernández-García, Fernando; Esteban-Burgos, Laura; Guerra, Carmen et al. (2021): RAF1 kinase activity is dispensable for KRAS/p53 mutant lung tumor progression. In: *Cancer cell* 39 (3), S. 294–296. DOI: 10.1016/j.ccell.2021.01.008.

**Reviewer 2:**

Reviewer #2: Authors Brandsetter et al. have presented findings that demonstrate a lack of therapeutic benefit from the combined inhibition of EGFR and RAF in two robust in vivo models, despite strong in vitro efficacy. In this reviewer's opinion, this "negative" finding makes an important contribution to the field of pancreatic cancer research. The reviewer enthusiastically agrees with the authors' conclusions, which advocate for the development of more effective pathway-targeted inhibitors and more accurate preclinical models. This is a direct, evidence-based recommendation for the research community.

The authors have appropriately selected strong KPC and orthotopic models that are universally accepted for their preclinical study aimed at dual inhibition, which is based on a sound scientific rationale. However, despite the clear evidence of in vitro work and laboratory experiments, there are several aspects that need clarification and further elaboration:

**Comment 1:**

1. The in vivo work presented lacks any morphological assessment, which is not reported in the methodology or results. The reviewer would like to see changes in H&E morphology, as well as in situ molecular analyses, such as immunohistochemistry (IHC) or RNA in situ hybridization. Specifically, this reviewer is interested in IHC work that could demonstrate reduced apoptosis, increased senescence, alterations in key proliferation markers, p-ERK levels, or the presence of immune cells. Such evidence would provide solid, in situ support for the authors' conclusion regarding a lack of tumor regression. This omission is critical, as documenting morphological and IHC evidence is standard practice in the preclinical field. Representative images showcasing vehicle treatment, monotherapies, and combined therapy, alongside relevant protein expression IHC work, would be beneficial.

**Answer to Comment 1:**

According to the reviewer’s suggestion, we performed different histopathological analysis. The necrotic area of each tumor was assessed by H/E staining. We quantified BrdU positive cells in the tumor to analyze difference in proliferation between the treatment groups. Apoptotic tumor cells were quantified by ApopTag® plus peroxidase. Additionally as potential resistance mechanism, we quantified pSTAT3 positive cells in the tumors. Histological analysis of tumors from the C57BL/6J mice are indicated in the new Supplementary Figure **S5** and for KPC mice in **S6 Fig**.

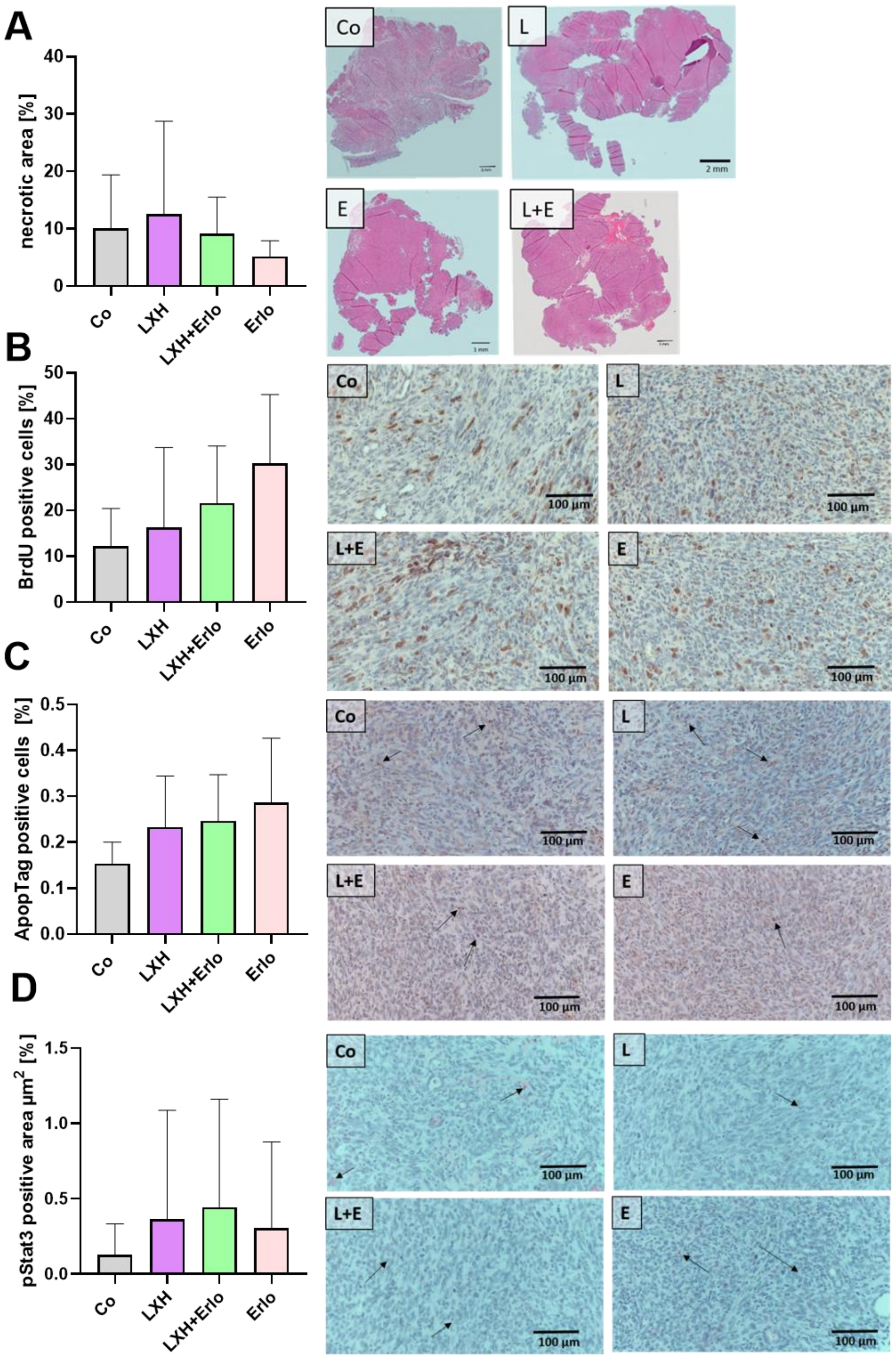


**S5 Fig. Immunohistological analysis of tumors from treated C57BL/6J mice.** Quantification of percentages of necrotic area via H/E staining (**A**), proliferating cells by BrdU incorporation (**B**), apoptotic cells via ApopTag® Peroxidase (**C**) and pSTAT3 positive area (**D**), respectively for control, LXH-254, LXH-254 plus erlotinib, or erlotinib treated mice. All staining's are illustrated in one exemplary picture for each treatment group. Statistics were carried out using one-way ANOVA with Tukey-test for multiple comparisons. Co: n=5-6, LXH: n=3-6, LXH+Erlo n=3-4, Erlo n=4-7.

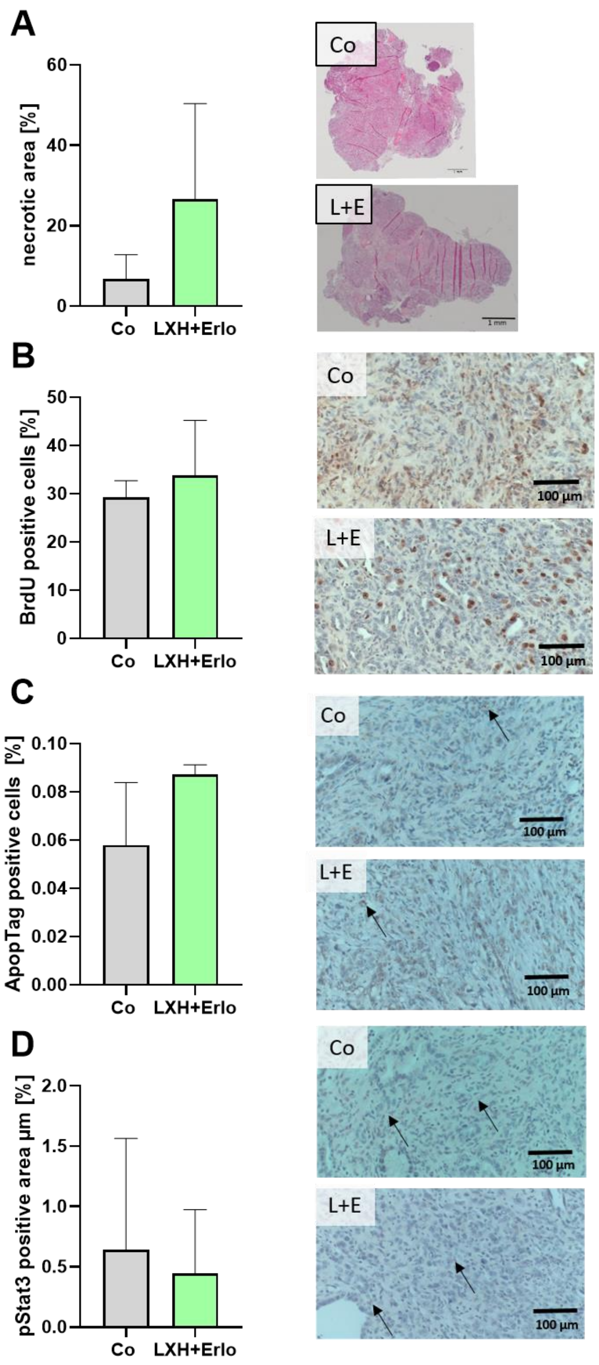


**S6 Fig. Immunohistological analysis of tumors from treated KPC mice.** Quantification of percentages of necrotic area via H/E staining (**A**), proliferating cells by BrdU incorporation (**B**), apoptotic cells via ApopTag® Peroxidase (**C**) and pSTAT3 positive area (**D**), respectively for control or LXH-254 plus erlotinib treated KPC mice. Statistics were carried out using Mann-Whitney test; Co: n=3, LXH+Erlo n=2-3

No significant differences were observed among the treatment groups for any of these parameters, including necrotic area, cell proliferation, apoptosis, or pSTAT3 positivity in either pancreatic cancer model. The interpretation of the histological analyses is also very difficult, as the tumors were preserved at different days after therapy, at the individual humane endpoint of each mouse. We added the aspect in the results part line 408-412 and discussed this observation in line 451-453 of the revised manuscript. In addition to tumor weight and survival, immunohistological analysis of the tumors, including assessment of necrotic area as well as proliferating and apoptotic cells also failed to reveal a detectable therapeutic effect of the treatment". The pSTAT3 detection is further discussed in line 532-538 of the revised manuscript. "One important resistance mechanism after combined RAF and EGFR ablation was

introduced from Blasco et al. by an increased activation of JAK-STAT signaling in resistant tumors (Blasco et al. 2019). We did not quantify a significant increase of pSTAT3-positive area after combinatorial treatment of LXH-254 and erlotinib in both pancreatic cancer models (S5D and S6D Fig.). Therefore, we cannot conclude that increased activation of JAK-STAT signaling pathway accounts for the observed therapeutic resistance".

**Comment 2:**

2. The Methods section should include more detail on how the drug doses were selected (e.g., based on maximum tolerated dose, previous literature, or pharmacokinetics/pharmacodynamics studies). This information is essential for reproducibility.

**Answer to Comment 2:**

We included the rational for the drug dosing in the material and methods in line 168-170 of the revised manuscript: "The dosing and the treatment duration was chosen in accordance with previous studies indicating a moderate inhibition of tumor progression and no toxicity in different rodent cancer models (Ramurthy et al. 2020; Iwai et al. 2012; Furugaki et al. 2011)."

**Comment 3:**

3. The Discussion should be expanded to hypothesize about the exact mechanisms underlying the in vivo failure. Possible factors to consider include whether the in vivo environment (e.g., stroma, immune cells, drug metabolism/pharmacokinetics) interfered with drug efficacy, or if the inhibition was simply insufficient given the complexities of the tumor microenvironment. Again, a morphological assessment conducted by an experienced pathologist, along with IHC evaluations, would provide valuable insight. I strongly recommend that the editorial board of PLOS One consider adding a co-author who is a pathologist to lend further credibility to the negative findings- if the authors request a change in authorship during revision phase.

**Answer to Comment 3:**

We performed extensive histological analysis of the tumors from all treatment groups. However, we did not observe significant differences in necrotic areas, proliferative activity or apoptotic cell numbers among the different treatment groups. Similarly, analysis of pSTAT3-positive cells revealed no significant differences in either animal model (**SFig. 5 and 6**, see **Comment 1**). Unfortunately, we were not able to acquire a pathologist for a clear feedback on the negative study results. Consequently, we were unable to identify or quantify a definitive mechanism of resistance associated with this therapeutic intervention. Consistent with the current literature, we propose that the most likely explanation for the lack of therapeutic efficacy is the limited in vivo activity of pharmacological RAF inhibition. We have expanded the discussion to include additional details on potential mechanism underlying this hypothesis in the discussion see line 549-558." Recently Sanclemente et al. used mouse strains that expressed knock-in alleles of two kinases-dead RAF1 variants (RAF1D468A, RAF1K375M) to demonstrate that the therapeutic benefit of RAF-1 ablation in KRAS/TRP53 mutant lung tumors is not mediated by inhibition of its kinase catalytic activity, but rather by the loss of its anti-apoptotic functions (Sanclemente et al. 2021). RAF-1 has been shown to interact with pro-apoptotic the kinases ASK1 and MST2 (Chen et al. 2001; O'Neill et al. 2004), thereby suppressing

apoptotic signalling. Consequently, instead of directly targeting of RAF1 kinase activity, future therapeutic strategies should focus on disrupting its anti-apoptotic, either by blocking its interaction with ASK1 and MST2 or by suppressing RAF-1 expression using selective RAF-1 degraders (Sanclemente et al. 2021) ."

**Comment 4:**

4. While I commend the authors for conducting a scientifically rigorous study, I recommend that they complete the work, as it currently feels unfinished. I suggest enhancing the figure panels by including gross tumor images that illustrate the sizes of tumors across the various treatment groups in comparison to the vehicle group, as well as in vitro images, rather than relying solely on bar plots and Kaplan-Meier plots. This would greatly improve the overall appeal and meaning of the study.

**Answer to Comment 4:**

We added in **Figure 1** images of the cells treated for 72 h with the specific therapeutics and concentrations. Unfortunately, we did not take comparative images of the tumors when the tissue was taken, since the mice had to be euthanized at different time points.

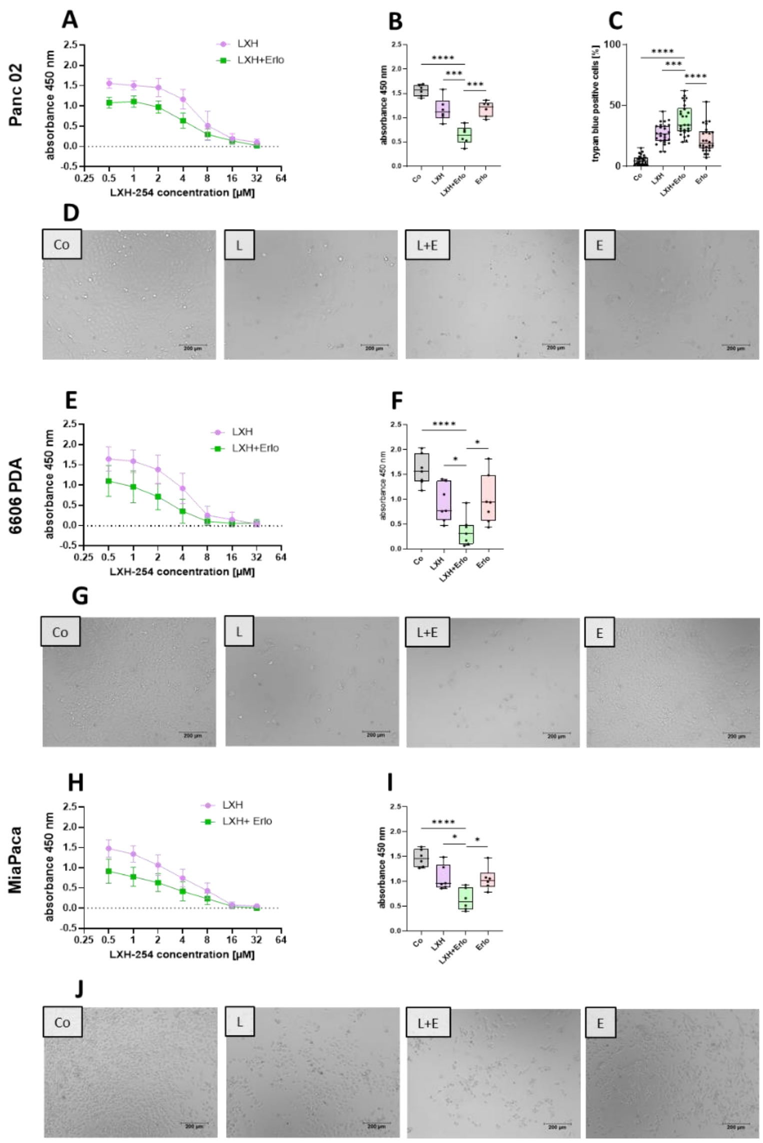


**Figure 1. Anti-cancer effects of the combination therapy on different pancreatic tumor cell lines by quantifying cell proliferation and cell death after 72 h.** Dose-dependent inhibition of murine Panc02 cell proliferation was quantified either after monotherapy with LXH-254 or after combination therapy with 10 µM erlotinib (**A**). Differences in tumor cell proliferation (**B**) and cell death rates (**C**) were investigated in Panc02 cells treated with vehicle (Co), 4 µM LXH-254 (LXH), 10 µM erlotinib (Erlo), or combination therapy (LXH+Erlo; **B**). Exemplary images of Panc02 cells treated for 72 h with either vehicle (Co), 4 µM LXH (L), in combination with 10 µM erlotinib (E+L) or monotherapy of 10 µM erotinib (E; **D**). Dose-response curve (**E**) and comparison of treatment groups (**F**) for 4 µM LXH-254 and combinatorial treatment with 10 µM erlotinib were quantified by proliferation assays in murine 6606PDA cells. Exemplary images of 6606 PDA cells with the different treatments after 72 h incubation time (**G**). Dose-dependent inhibition of proliferation of human Mia PaCa-2 cells by LXH-254 and the combination with 15 µM erlotinib (**H**). Differences in Mia PaCa-2 cell proliferation in the vehicle (Co), 2 µM LXH-254 (LXH), 15 µM erlotinib (Erlo) and the combinatorial treatment (LXH+Erlo) (**I**). Images of MiaPaca cells after treatment for 72 h hours with the mentioned concentrations and therapeutics (**J**). Statistics were carried out using one-way ANOVA with Tukey-test for multiple comparisons. * p≤0.05, *** p≤0.001, **** p≤0.0001; A, B: n = 6; C: n = 26; D, E: n = 7; F: n = 7; G: n = 6.

## References Reviewer 2


Blasco, María Teresa; Navas, Carolina; Martín-Serrano, Guillermo; Graña-Castro, Osvaldo; Lechuga, Carmen G.; Martín-Díaz, Laura et al. (2019): Complete Regression of Advanced Pancreatic Ductal Adenocarcinomas upon Combined Inhibition of EGFR and C-RAF. In: *Cancer cell* 35 (4), 573-587.e6. DOI: 10.1016/j.ccell.2019.03.002.

Chen, J.; Fujii, K.; Zhang, L.; Roberts, T.; Fu, H. (2001): Raf-1 promotes cell survival by antagonizing apoptosis signal-regulating kinase 1 through a MEK-ERK independent mechanism. In: *Proceedings of the National Academy of Sciences of the United States of America* 98 (14), S. 7783–7788. DOI: 10.1073/pnas.141224398.

Furugaki, Koh; Moriya, Yoichiro; Iwai, Toshiki; Yorozu, Keigo; Yanagisawa, Mieko; Kondoh, Kumiko et al. (2011): Erlotinib inhibits osteolytic bone invasion of human non-small-cell lung cancer cell line NCI-H292. In: *Clinical & experimental metastasis* 28 (7), S. 649–659. DOI: 10.1007/s10585-011-9398-4.

Iwai, Toshiki; Moriya, Yoichiro; Shirane, Masatoshi; Fujimoto-Ouchi, Kaori; Mori, Kazushige (2012): Continuous inhibition of epidermal growth factor receptor phosphorylation by erlotinib enhances antitumor activity of chemotherapy in erlotinib-resistant tumor xenografts. In: *Oncology reports* 27 (4), S. 923–928. DOI: 10.3892/or.2011.1614.

O'Neill, Eric; Rushworth, Linda; Baccarini, Manuela; Kolch, Walter (2004): Role of the kinase MST2 in suppression of apoptosis by the proto-oncogene product Raf-1. In: *Science (New York, N.Y.)* 306 (5705), S. 2267–2270. DOI: 10.1126/science.1103233.

Ramurthy, Savithri; Taft, Benjamin R.; Aversa, Robert J.; Barsanti, Paul A.; Burger, Matthew T.; Lou, Yan et al. (2020): Design and Discovery of N-(3-(2-(2-Hydroxyethoxy)-6-morpholinopyridin-4-yl)-4-methylphenyl)-2-(trifluoromethyl)isonicotinamide, a Selective, Efficacious, and Well-Tolerated RAF Inhibitor Targeting RAS Mutant Cancers: The Path to the Clinic. In: *Journal of medicinal chemistry* 63 (5), S. 2013–2027. DOI: 10.1021/acs.jmedchem.9b00161.

Sanclemente, Manuel; Nieto, Patricia; Garcia-Alonso, Sara; Fernández-García, Fernando; Esteban-Burgos, Laura; Guerra, Carmen et al. (2021): RAF1 kinase activity is dispensable for KRAS/p53 mutant lung tumor progression. In: *Cancer cell* 39 (3), S. 294–296. DOI: 10.1016/j.ccell.2021.01.008.
