# Supplementary material for: Targeting pancreatic cancer with combined inhibition of EGFR and RAF
Source: PLoS One. 2026 Apr 24;21(4):e0347843. doi: 10.1371/journal.pone.0347843 (PMC13108728; doi:10.1371/journal.pone.0347843)
Supplement: Attachment — Submitted filename: Response_to_Reviewer_auresp_2.pdf [file pone.0347843.s011.pdf]

Point to point Reply

Reviewer 1

Comment 1:

Reviewer #1: The authors have addressed all the comments thoroughly and have provided clear responses to every point raised

Answer to Comment 1:

We thank the reviewer for his assessment of the manuscript.

Reviewer 2:

Comment 1:

Reviewer #2: The histology images and immunohistochemistry (IHC) data, as in-situ analyses, could be incorporated into the main figure. Several of the cell line proliferation images appear redundant and lack sufficient resolution. I believe the authors have not sufficiently emphasized the significance of their findings. The manuscript and the evidence derived from negative research findings should be presented with the same rigor as positive results. This principle has been highlighted by other authors and serves to encourage researchers to report scientifically sound negative findings. The final image panels, as currently constructed, lack visual impact. I recommend reorganizing these panels by incorporating the new data presented in the supplementary materials. I leave the final decision to editorial board on this.

Answer to Comment 1:

According to the reviewer's suggestions, we have incorporated the histological analysis of the tumors into the revised manuscript as main **Figure 4**.

In addition, we adjusted the contrast of the cell line images to improve clarity. The resolution of these images in the TIFF- files is high to ensure optimal image quality.

To improve the overall structure and the readability of the manuscript, **Figure.1** now presents only the proliferation and cell death analysis from the Panc02 cells, which were subsequently used for the western blot analysis (Fig. 2) and the orthotopic murine cancer model (Fig. 3 and 4).

The proliferation data from the other murine and humane cell lines are now provided in **Supplementary Figure 1**.

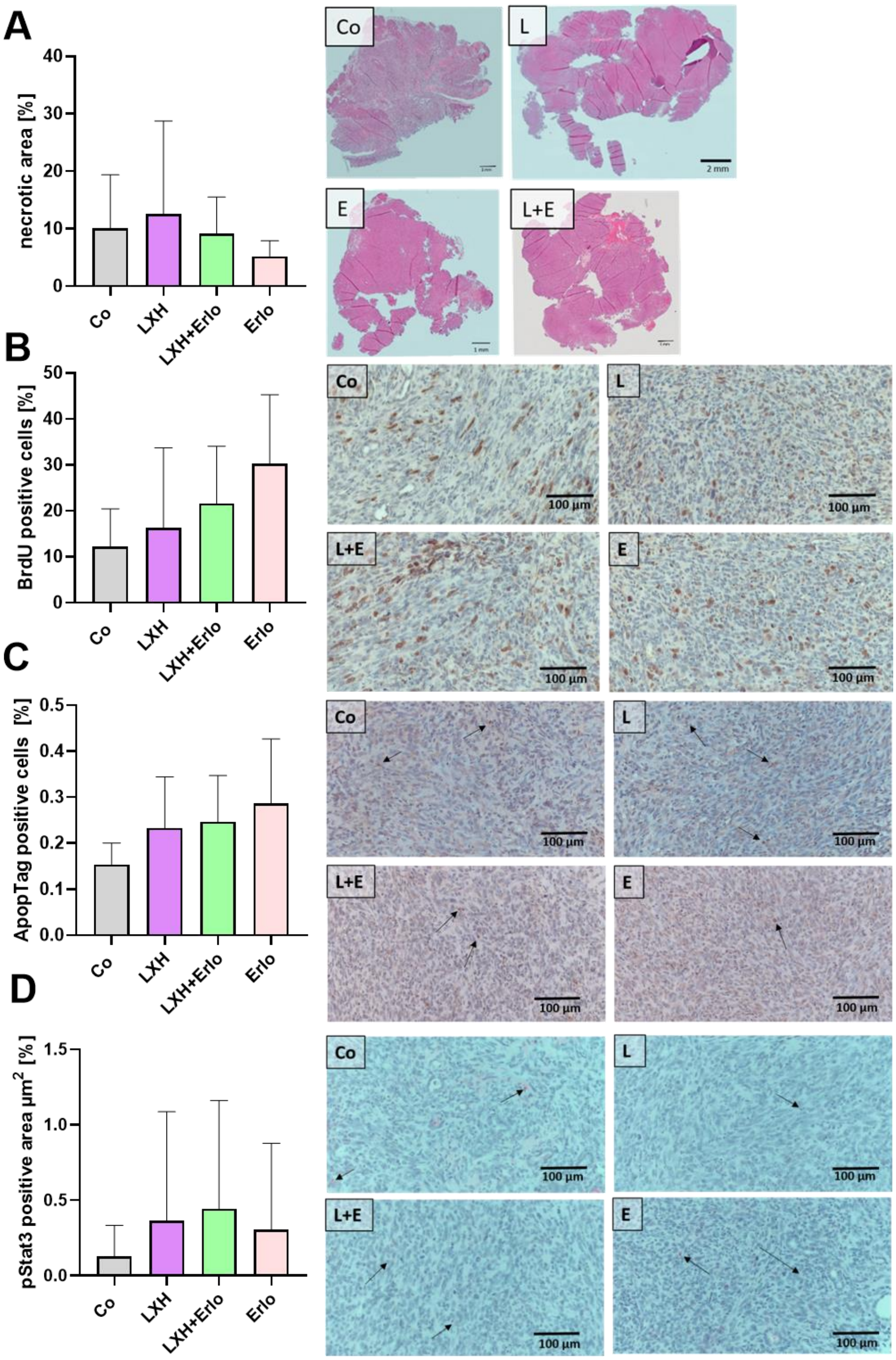

Figure 4. Immunohistological analysis of tumors from treated C57BL/6J mice. Quantification of percentages of necrotic area via H/E staining (**A**), proliferating cells by BrdU incorporation (**B**), apoptotic cells via ApopTag® Peroxidase (**C**) and pSTAT3 positive area (**D**), respectively for control, LXH-254, LXH-254 plus erlotinib, or erlotinib treated mice. All staining's are illustrated in one exemplary picture for each treatment group. Arrows marking the specific stained cells, or area. Statistics were carried out using one-way ANOVA with Tukey-test for multiple comparisons. Co: n=5-6, LXH: n=3-6, LXH+Erlo n=3-4, Erlo n=4-7.

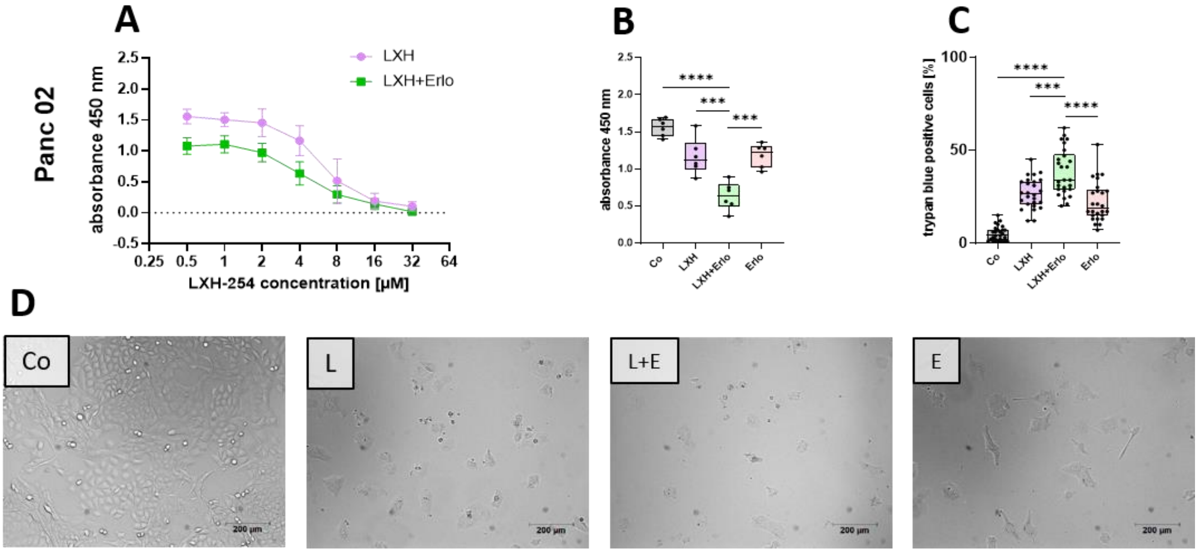

Figure 1. Anti-cancer effects of the combination therapy on the murine pancreatic tumor cell line Panc02 by quantifying cell proliferation and cell death after 72 h. Dose-dependent inhibition of murine Panc02 cell proliferation was quantified either after monotherapy with LXH-254 or after combination therapy with 10 µM erlotinib (**A**). Differences in tumor cell proliferation (**B**) and cell death rates (**C**) were investigated in Panc02 cells treated with vehicle (Co), 4 µM LXH-254 (LXH), 10 µM erlotinib (Erlo), or combination therapy (LXH+Erlo; **B**). Exemplary images of Panc02 cells treated for 72 h with either vehicle (Co), 4 µM LXH (L), in combination with 10 µM erlotinib (E+L) or monotherapy of 10 µM erotinib (E; **D**). Statistics were carried out using one-way ANOVA with Tukey-test for multiple comparisons. *** p≤0.001, **** p≤0.0001; A, B: n = 6; C: n = 26.

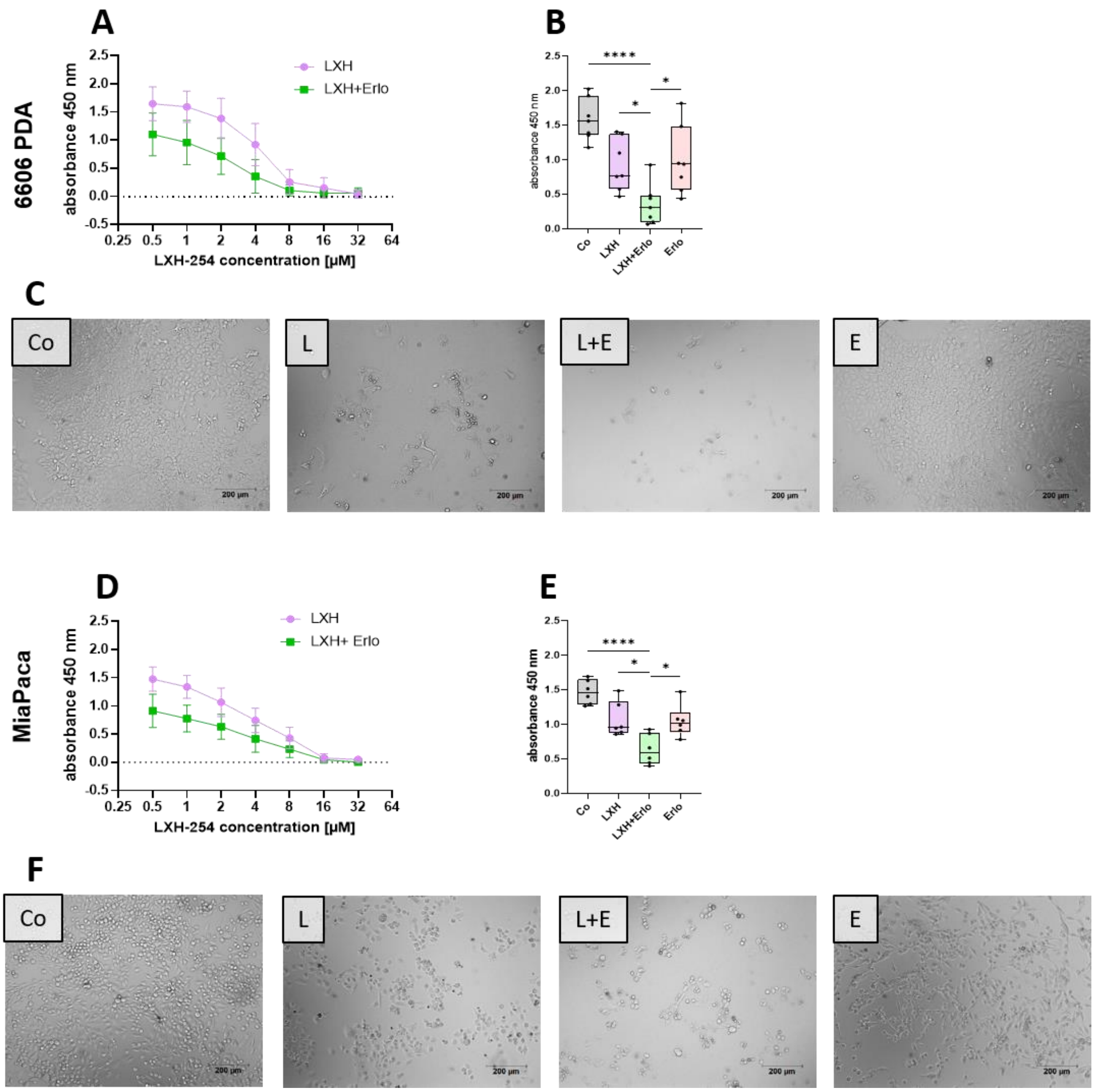

S1 Fig. Anti-cancer effects of the combination therapy on the murine and human pancreatic tumor cell lines by quantifying cell proliferation and cell death after 72 h. Dose-response curve (**A**) and comparison of treatment groups (**B**) for 4 µM LXH-254 and combinatorial treatment with 10 µM erlotinib were quantified by proliferation assays in murine 6606PDA cells. Exemplary images of 6606 PDA cells with the different treatments after 72 h incubation time (**C**). Dose-dependent inhibition of proliferation of human Mia PaCa-2 cells by LXH-254 and the combination with 15 µM erlotinib (**D**). Differences in Mia PaCa-2 cell proliferation in the vehicle (Co), 2 µM LXH-254 (LXH), 15 µM erlotinib (Erlo) and the combinatorial treatment (LXH+Erlo) (**E**). Images of MiaPaca cells after treatment for 72 h hours with the mentioned concentrations and therapeutics (**F**). Statistics were carried out using one-way ANOVA with Tukey-test for multiple comparisons. * $p \leq 0.05$, *** $p \leq 0.001$, **** $p \leq 0.0001$; A, B: n = 7; D: n = 7; E : n = 6.
